# Identification of a potent small-molecule inhibitor of bacterial DNA repair that potentiates quinolone antibiotic activity in methicillin-resistant *Staphylococcus aureus*^☆^

**DOI:** 10.1016/j.bmc.2019.06.025

**Published:** 2019-10-15

**Authors:** Carine S.Q. Lim, Kam Pou Ha, Rebecca S. Clarke, Leigh-Anne Gavin, Declan T. Cook, Jennie A. Hutton, Charlotte L. Sutherell, Andrew M. Edwards, Lindsay E. Evans, Edward W. Tate, Thomas Lanyon-Hogg

**Affiliations:** a Department of Chemistry, Molecular Sciences Research Hub, Imperial College London, London W12 0BZ, UK; b MRC Centre for Molecular Bacteriology and Infection, Department of Medicine, Imperial College London, London SW7 2AZ, UK

**Keywords:** Antibiotic resistance, Target validation, DNA repair, Drug synergy, IMP-1700

## Abstract

The global emergence of antibiotic resistance is one of the most serious challenges facing modern medicine. There is an urgent need for validation of new drug targets and the development of small molecules with novel mechanisms of action. We therefore sought to inhibit bacterial DNA repair mediated by the AddAB/RecBCD protein complexes as a means to sensitize bacteria to DNA damage caused by the host immune system or quinolone antibiotics. A rational, hypothesis-driven compound optimization identified **IMP-1700** as a cell-active, nanomolar potency compound. **IMP-1700** sensitized multidrug-resistant *Staphylococcus aureus* to the fluoroquinolone antibiotic ciprofloxacin, where resistance results from a point mutation in the fluoroquinolone target, DNA gyrase. Cellular reporter assays indicated **IMP-1700** inhibited the bacterial SOS-response to DNA damage, and compound-functionalized Sepharose successfully pulled-down the AddAB repair complex. This work provides validation of bacterial DNA repair as a novel therapeutic target and delivers **IMP-1700** as a tool molecule and starting point for therapeutic development to address the pressing challenge of antibiotic resistance.

## Graphical abstract

**Figure:**
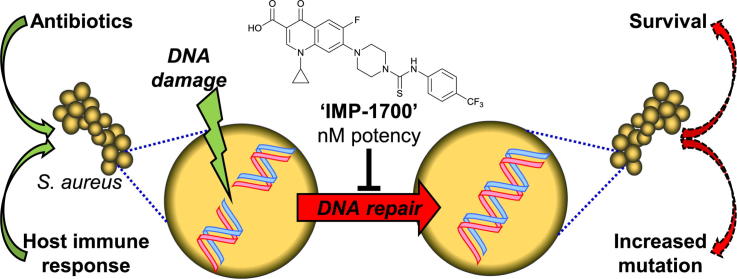

## 1 Introduction

The increase in multi-drug resistant pathogenic bacteria presents one of the most serious threats to human health globally, threatening to render application of numerous medical advances such as surgery and chemotherapy so life-threatening as to be impractical. Resistance has emerged to all clinical antibiotics, and the perceived low profitability of antibiotic development has resulted in an insufficient pipeline of new therapeutics.^1^, ^2^ Many antibiotics in late-stage clinical development function through established pathways or targets rather than novel mechanisms, resulting in short-lived efficacy improvements.^3^ There is therefore a pressing unmet need for identification of new compounds with novel mechanisms of action (MOA).

Antibiotics that function through disruption of essential cellular functions for growth and survival present several drawbacks. Inherently, such compounds provide a strong driving force for resistance selection, both *in vivo* and when compounds are excreted or released into the environment. Such antibiotics also target the normal host microbiota as well as pathogenic bacteria, resulting in a range of side effects and providing opportunities for secondary infections to occur.^4^ Novel modes of action for antibiotics are required to circumvent these limitations. One approach is to develop compounds that are conditionally lethal, and target functions essential for the *in vivo* viability of pathogens during infection.^5^

During infection the host innate immune response generates an oxidative burst which damages pathogen DNA. A single DNA double-strand break (DSB) is lethal to bacteria if left unrepaired before cell division.^6^, ^7^ The process of homologous recombination to repair DSBs is initiated by the AddAB or RecBCD helicase-nuclease complexes. The complex binds blunt end double-strand (ds) DNA and unwinds the duplex in an ATP-dependent manner, with simultaneous degradation of the resultant single-strand (ss) DNA.^8^, ^9^ When the complex encounters a crossover hot-spot instigator (Chi) sequence in the 3′-terminated strand, degradation of the 5′-terminated strand increases, resulting in a 3′ overhang.^9^, ^10^ The RecA protein binds the 3′ overhang and initiates recombination and repair processes.^8^, ^11^ As DSB repair is critical in protecting pathogenic bacteria from the host immune response,^12^, ^13^ the enzymes involved in this process represent novel therapeutic targets to promote immune clearance of infections. The narrow window for resistance selection, occurring only in the context of an immune response, may limit the rate of emergence of resistance. Furthermore, such an approach is not expected to disturb the commensal microbiome as it is not targeted by the immune system, thus reducing susceptibility to opportunistic secondary infections. The quinolone class of antibiotics act through generation of DSBs by inhibition of DNA gyrase, and the fluoroquinolone ciprofloxacin (CFX) is among the most commonly used antibiotics worldwide.^14^ The DSB repair process offers protection from quinolones,^12^, ^15^, ^16^ with *recB* or *recC* gene knockouts in *Escherichia coli* increasing susceptibility to CFX. Further, *recC* mutants display partial sensitivity restoration in *E. coli* strains possessing the Ser83Leu DNA gyrase mutation, which typically confers a high level of CFX resistance.^17^ Small-molecule inhibition of DNA repair may therefore synergistically increase the efficacy of DNA damaging antibiotics, or sensitize resistant bacteria to this class of molecules.

AddAB/RecBCD-mediated DSB repair also initiates the bacterial SOS response through binding of RecA to ssDNA and the subsequent degradation of LexA repressor proteins. This leads to the expression of low fidelity polymerases that mediate error-prone DNA replication, resulting in an increased rate of mutagenesis.^18^, ^19^ Inhibition of the DNA repair pathway may therefore reduce resistance acquisition or host adaptation by blocking the mutagenic SOS response. Co-administration of DNA-repair inhibitors alongside DNA-damaging antibiotics such as CFX not only suggests potential for synergistic drug combinations but could also prolong clinical lifespan.

The conservation of AddAB/RecBCD enzyme classes in ∼90% of all bacteria species^20^ suggests that inhibitors of these complexes would be a potentially broad-spectrum therapeutic approach. Various inhibitors of AddAB/RecBCD function have been previously reported in the literature. Adozelesin, ecteinascidin 743, hedamycin, cisplatin, and psoralen inhibit RecBCD function,^21^, ^22^ however, the MOA of these molecules through DNA-alkylation renders them unselective and highly cytotoxic.^23^ The Gam protein of bacteriophage lambda is an inhibitor of RecBCD which functions through competition for the DNA-binding site. Recombinant expression of Gam enhances quinolone sensitivity in *E. coli* and *Klebsiella pneumoniae*,^24^ however, the poor *in vivo* stability, cell uptake and oral bioavailability of proteins and peptides is a significant challenge to developing a successful Gam-based therapeutic. ML328 (**1**) was reported as a small-molecule inhibitor of AddAB/RecBCD in both cellular and biochemical assays,^25^ however, its utility is limited by moderate cellular potency.

We therefore sought to develop inhibitors of the AddAB/RecBCD DNA repair complexes, using **1** as a starting point. Hypothesis-driven optimization generated **IMP-1700**, which was capable of synergistic sensitization of resistant *Staphylococcus aureus* to CFX with single-digit nanomolar potency. This work demonstrates the therapeutic potential of targeting bacterial DNA repair as a novel means to combat resistance and identifies **IMP-1700** as a valuable tool molecule for future studies and development.

## 2 Results and discussion

### 2.1 Design and synthesis of scaffold-hop analogues of ML328

**Scheme 1:**
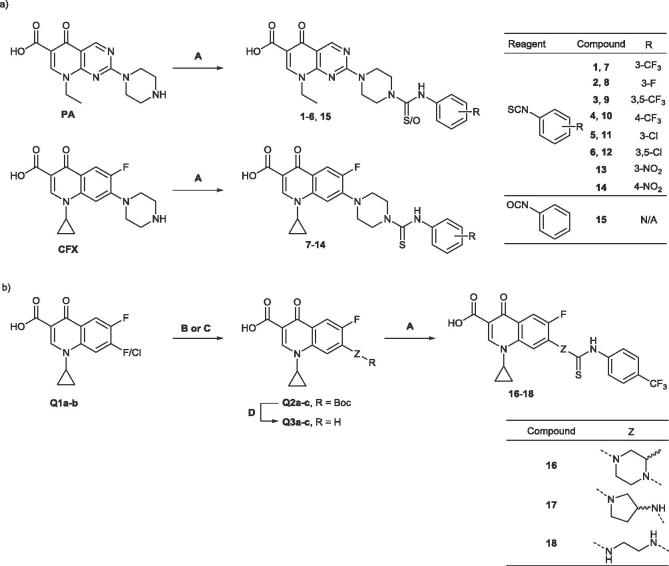
Synthetic routes to analogues and structures. General procedures: (A) NaHCO_3_, DMF, RT, overnight; (B) DMSO, microwave (115 °C, 3 h); (C) pyridine, reflux, overnight; (D) 4 M HCl, dioxane, RT, overnight.

The pipemidic acid arylthiourea ML328 (**1**) was identified through a cellular DNA repair high-throughput screen, and subsequently demonstrated to inhibit purified AddAB and RecBCD.^25^ The pipemidic acid (PA) moiety of **1** is a member of the quinolone family of antibiotics, which inhibit DNA gyrase and topoisomerase IV through stabilization of the enzyme-DNA complex via intercalation into the DNA substrate.^14^ It was therefore postulated that inhibition of AddAB/RecBCD by **1** may occur through a similar mechanism. CFX is an optimized derivative of quinolones such as PA, where the *N*-1-cyclopropyl^26^ and C-6 fluorine^27^ substituents modulate electron density in the aromatic quinolone ring to favor intercalation, resulting in significant improvements in activity.^28^, ^29^ We therefore hypothesized that AddAB/RecBCD inhibition may be improved via a scaffold-hop from the PA to a CFX core through a similar mechanism of improved intercalation. Published SAR demonstrates the necessity of the arylthiourea moiety for AddAB/RecBCD nuclease inhibition, with meta–trifluoromethyl substitution favored over ortho-.^25^ PA derivatives **1**–**6** and scaffold-hop CFX derivatives **7**–**14** were prepared from the required core and corresponding isothiocyanates in good yield (Scheme 1), with substitution of the aryl moiety guided by the Topliss decision tree.^30^ Negative control compound **15**, which has been shown not to inhibit the purified enzymes, was prepared from PA and phenyl isocyanate.

Substitution of the piperazine moiety of CFX derivatives was achieved through reaction of mono-Boc protected diamines with halofluoroquinolones (**Q1a-b**). Microwave-assisted nucleophilic aromatic substitution of the C-7 chlorine of 7-chloro-quinoline acid (**Q1a**), allowed shorter reaction times and prevented side-product formation arising from C-6 substitution.^31^ This strategy was effective for reaction of **Q1a**, with secondary amines 3-(Boc-amino)pyrrolidine and 1-*N*-Boc-2-methylpiperazine, yielding the corresponding C-7 displacement products **Q2a-b**. Reaction of **Q1a** with *N*-Boc-ethylenediamine resulted in formation of both C-6 and C-7 displacement products, which could not be separated. 6,7-Difluoroquinoline carboxylic acid **Q1b** was reacted with *N*-Boc-ethylenediamine in a 1:1 ratio, allowing formation of the required C-7 displacement product **Q2c** in good yield. Boc-deprotection of the aminated quinolones using HCl/dioxane afforded the corresponding chloride salts **Q3a-c**. These were used directly for coupling with 4-(trifluoromethyl)phenyl isothiocyanate to form **16**–**18**.

### 2.2 Biological evaluation of potentiation of DNA damage

Minimum inhibitory concentration (MIC) assays were used to evaluate the antimicrobial activity of compounds against Gram-negative (*E. coli*) and Gram-positive (*S. aureus*) bacteria. The MIC is defined as the lowest concentration of a compound generating no visible bacterial growth following static incubation at 37 °C in air for 18 h. To allow quantitative efficacy comparison across compounds, half-maximal effect concentration (EC_50_) values were obtained from non-linear regression analysis of optical density measurements at 600 nm (OD_600_) after 18 h in response to two-fold serial dilutions of test compounds. Compounds were tested against *E. coli* K-12 BW25113, which is the wild-type strain used in the Keio Knockout Collection (Table S1) and is sensitive to CFX (EC_50_ = 22 ± 2 nM, Table 1).^32^ PA derivatives **1**–**6** exhibited EC_50_ values > 100 µM; whereas CFX derivatives **7**–**12** generated EC_50_ values 0.2–0.5 µM, with compound **8** (3-F) showing approximately 2-fold higher potency than other CFX derivatives (Table 1). Gram-positive bacteria are more susceptible to small-molecule penetration, and compounds were therefore assessed for growth inhibition against the *S. aureus* SH1000 strain. SH1000 is derived from the *S. aureus* 8325–4 strain with a repaired *rsbU* gene (Table S1) and is sensitive to ciprofloxacin (EC_50_ = 0.35 ± 0.07 µM, Table 1).^33^ **1**–**6** exhibited EC_50_ values from 2 to 8 µM, indicating a >10-fold increase in potency against SH1000 compared to *E. coli*. **6** (3,5-Cl) showed the highest potency (EC_50_ = 2.3 ± 0.3 µM), representing > 50-fold increase in potency against SH1000 compared to *E. coli* (Table 1)*.* CFX derivatives **7**–**12** again demonstrated higher potency than the corresponding PA derivatives with EC_50_ values between 0.05 and 1.5 µM (Table 1), corresponding to 2–5-fold increase in potency compared to CFX derivatives in *E. coli*. Interestingly, **9** (3,5-CF_3_) was the only compound to display a decrease in potency against SH1000, being ∼5-fold more potent against *E. coli*.

**Table 1:** Biological activity of 1–15. Compounds were tested as single agents against *E. coli* (K-12 BW25113), *S. aureus* (SH1000), methicillin-resistant *S. aureus* (MRSA, USA300 JE2), and for Ciprofloxacin (CFX) potentiation in MRSA. EC_50_ values determined by dose–response non-linear regression. ^a^Urea instead of thiourea. ^b^EC_50_ not determined due to solubility limitations. (n/a = not applicable). Data represent mean ± SEM (n = 3 biological replicates).

			EC_50_ (μM)			
Compound	Core	R	*E. coli* (K12 BW25113) Compound only	*S. aureus* (SH1000) Compound only	*S. aureus* (USA300 JE2) Compound only	*S. aureus* (USA300 JE2) Compound + CFX (9.4 μM)
1 (ML328)	PA	3-CF_3_	>100	8.2 ± 1.7	6.9 ± 0.6	1.1 ± 0.1
2	PA	3-F	>100	6.9 ± 0.4	27 ± 0.4	0.73 ± 0.12
3	PA	3,5-CF_3_	>100	3.6 ± 0.4	2.7 ± 0.6	0.62 ± 0.09
4	PA	4-CF_3_	>100	5.2 ± 1.1	4.0 ± 0.3	0.24 ± 0.03
5	PA	3-Cl	>100	6.0 ± 1.5	6.9 ± 0.1	0.31 ± 0.02
6	PA	3,5-Cl	>100	2.3 ± 0.3	1.8 ± 0.1	0.12 ± 0.04
7	CFX	3-CF_3_	0.41 ± 0.00	0.19 ± 0.06	3.7 ± 0.5	0.14 ± 0.00
8	CFX	3-F	0.24 ± 0.03	0.08 ± 0.01	4.8 ± 0.9	0.22 ± 0.05
9	CFX	3,5-CF_3_	0.39 ± 0.01	1.5 ± 0.3	3.5 ± 0.4	0.47 ± 0.07
10 (IMP1700)	CFX	4-CF_3_	0.50 ± 0.05	0.21 ± 0.03	3.8 ± 0.5	0.0059 ± 0.0006
11	CFX	3-Cl	0.46 ± 0.10	0.12 ± 0.04	2.8 ± 0.1	0.084 ± 0.007
12	CFX	3,5-Cl	0.42 ± 0.02	0.084 ± 0.007	3.6 ± 0.4	0.043 ± 0.011
13	CFX	3-NO_2_	0.27 ± 0.02	0.11 ± 0.03	>25^b^	0.43 ± 0.09
14	CFX	4-NO_2_	0.26 ± 0.04	0.050 ± 0.008	>12.5^b^	0.049 ± 0.006
15^a^	PA	H	>12.5^b^	>12.5^b^	>12.5^b^	>12.5^b^
CFX	n/a	n/a	0.022 ± 0.002	0.35 ± 0.07	8.3 ± 1.9	n/a

Methicillin-resistant *S. aureus* (MRSA) is a multi-drug resistant bacterium responsible for a large number of nosocomial infections.^34^ The emergence of community associated (CA)-MRSA capable of infection of healthy individuals highlights its significance and clinical relevance.^35^ The MRSA strain USA300 JE2 is a plasmid-cured derivative of strain LAC, which is chromosomally encoded for resistance to β-lactams and CFX (Table S1). β-lactam resistance is conferred through the *mecA* gene; CFX resistance is conferred through a Ser84Leu point mutation in DNA gyrase.^36^ JE2 exhibited > 20-fold higher tolerance of CFX (EC_50_ = 8.3 ± 1.9 µM, Table 1) than the CFX-sensitive *S. aureus* strain SH1000 (EC_50_ = 0.35 ± 0.07 µM). The compound library was therefore assessed for growth inhibition of JE2, which demonstrated a general trend for decreased potency against JE2 compared to SH1000 (Table 1). Interestingly, CFX derivatives **7**, **8**, **10**, **11**, and **12** appeared > 20-fold less potent, suggesting a MOA as a single-agent that may, in part, involve DNA gyrase.

The postulated inhibition of AddAB in *S. aureus* may not be expected to result in growth inhibition of bacteria not subjected to DNA-damaging stress, therefore the observed antibiotic activity may result from DNA gyrase inhibition, some as-yet unidentified off-target, or indicate a polypharmacological MOA at higher concentrations. However, inhibition of the DNA repair process was proposed to allow sensitization of bacteria to DNA-damaging antibiotics to which they have acquired resistance.^24^. The compound library was therefore assessed for potentiation of JE2 killing by half-MIC CFX (9.4 µM). All compounds showed > 4-fold potentiation by CFX compared to use as single agents, with CFX analogues (**7**, **8**, **10**–**12**) showing > 3-fold increased potentiation compared to their PA counterparts. 3,5-CF_3_ derivatives (**3** and **9)** demonstrated approximately equal potency with PA and CFX cores. PA derivative **5** (3-Cl) possessed approximately equal potency as a single agent against SH1000 and JE2 (EC_50_ = ∼6 µM), with ∼20-fold increase in potency in the presence of CFX, suggesting on-target activity.

Collectively, these data demonstrated that CFX derivatives were more active than corresponding PA derivatives as single agents or when potentiated by CFX (with the noted exception of **9**), consistent with the hypothesis of improved intercalation increasing potency. Analysis of available literature SAR data for the PA series^25^ with Topliss-guided analysis indicated improved activity correlated with increased electron-withdrawing ability of the substituent on the thiourea-linked aryl moiety. Potentiation by CFX demonstrated favorability of para- (**4** and **10**) over meta- (**1** and **7**) trifluoromethyl substituent in both the PA (∼5-fold increase) and CFX (∼25-fold increase) series (Table 1). Compound **10** comprising a CFX core and a *para*-substituted trifluoromethyl arylthiourea moiety was the most potent analogue, and exhibited a ∼650 increase in potency against JE2 in the presence of CFX (EC_50_ = 5.9 ± 0.6 nM). This represents ∼160-fold increase in potency compared to **1** (EC_50_ = 1.1 ± 0.1 μM) for potentiation against JE2 by CFX.

The increased activity of electron-withdrawing arylthiourea substituents in the CFX series led to the synthesis of nitro derivatives at the meta- (**13**) and para- (**14**) positions, following the Topliss decision tree and empirical antibacterial activities linked to the nitroaromatic group.^30^, ^37^ Para-substitution resulted in a ∼10-fold increase in potency compared to meta. However, metabolic toxicities, particularly hepatotoxicity, are associated with nitroaromatic groups, as a result of their abilities to interfere with cellular redox via nitro reduction,^38^ and the lack of improved bioactivity for **13** and **14** compared to **10** (Table 1) implied no justification for their continued investigation.

To support the proposal that compound potentiation by CFX occurred by inhibition of AddAB-mediated DNA repair, PA urea derivative **15** was prepared as this compound does not inhibit the activity of purified AddAB/RecBCD.^25^ Consistent with the postulated mechanism, the potency of **15** was unaffected by the presence of CFX (Table 1), suggesting potentiation by CFX may be mediated by AddAB inhibition. The analogue panel was further tested for potentiation of alternative DNA-damaging agents Mitomycin-C (MMC) and hydrogen peroxide (Table S2), which function via alkylation and oxidization DNA bases, respectively. No potentiation of MMC or hydrogen peroxide was observed, consistent with repair of these forms of DNA damage typically occurring through base excision or mismatch repair.

As a result of the successful scaffold hop to the CFX core, further SAR optimization strategies that improve quinolone substituent antibiotic activity were investigated. The quinolone C-7 modulates factors such as antibacterial spectrum, bioavailability and side-effects.^39^ Derivatives of the most potent analogue **10**, were prepared replacing the central piperazine moiety with 3-methylpiperazine (**16**), 3-aminopyrrolidine (**17**), or ethylenediamine (**18**). **16**–**18** were tested in cellular growth inhibition assay either as single agents against *E. coli*, *S. aureus* SH1000 (Table S3), or as single agents and in combination with CFX against USA300 JE2 (Table 2). None of these derivatives were more effectively potentiated by CFX than **10**, indicating deviation from the piperazine ring of **10** is not favored (Table 2). The substantial decrease in bioactivity of ethylenediamine-derivative **18**, is likely due to increased entropic penalty on binding as a result of increased flexibility.

**Table 2:** Potentiation of Ciprofloxacin by derivatives of 10 in MRSA (USA300 JE2). Compounds were tested as single agents and for Ciprofloxacin (CFX) potentiation in MRSA. Data represent mean ± SEM (n = 3 biological replicates).

Compound	Linker	EC_50_ (μM)	
		Compound only	Compound + CFX (9.4 μM)
**16**	3-methylpiperazine	3.9 ± 0.4	0.018 ± 0.003
**17**	3-aminopyrrolidine	53 ± 13	3.2 ± 0.3
**18**	ethylenediamine	9.7 ± 1.0	2.5 ± 0.3

### 2.3 Demonstration of synergistic effects with Ciprofloxacin

**Figure 1:**
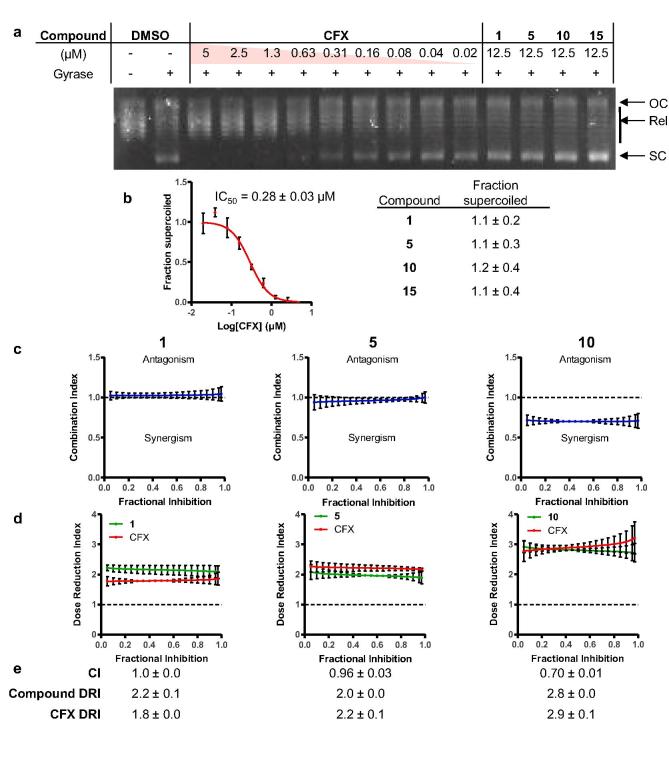
Compounds do not inhibit DNA gyrase to exhibit synergy with ciprofloxacin. a) Effect of compounds on *E. coli* DNA gyrase supercoiling of pBR322. Gyrase is inhibited by Ciprofloxacin (CFX), but not **1**, **5**, **10** or **15** (OC = open-circular DNA; Rel = relaxed DNA; SC = supercoiled DNA). b) Densitometry analysis of DNA supercoiling. Data represent mean ± SEM (n = 3 technical replicates). c) Synergy quantification of **1**, **5** and **10** with CFX using CompuSyn software simulation, displayed as combination index (CI) against fraction affected (Fa). d) Dose-reduction index (DRI) of compound or CFX against Fa. e) Summary of CI, compound and CFX DRI. Only compound **10** (**IMP-1700**) quantitatively synergizes with CFX. Data represent mean ± SEM (n = 3 biological replicates).

The use of SAR optimization strategies based on the development of quinolone antibiotics, and loss of single agent potency against JE2 compared to SH1000, prompted consideration that compounds may be acting through quinolone targets, rather than AddAB. The presented panel of inhibitors contain PA or CFX cores, both known inhibitors of DNA gyrase and topoisomerase IV. DNA gyrase produces supercoiled DNA from relaxed and open-coiled DNA, which can be separated via agarose gel electrophoresis. Inhibition of pBR322 supercoiling by DNA gyrase was assessed for hit compound **1**, best performing compound **10**, alongside the potentially on-target derivative **5**, inactive control **15** and positive control CFX. (Figure 1A). CFX inhibited gyrase (IC_50_ = 0.28 ± 0.03 μM), whereas **1**, **5**, **10**, and **15** showed minimal activity against gyrase at 12.5 μM (Figure 1B). Similarly, topoisomerase IV was not inhibited by **1**, **10**, or **15** at 12.5 µM (Figure S1). This concentration is higher than the cellular EC_50_ for growth inhibition for all active compounds and provides initial evidence that DNA gyrase and topoisomerase IV are not direct targets of this series of compounds. Indeed, *N*-acylation is a known mechanism for loss of antibiotic activity in fluoroquinolones.^40^

To further probe if compounds functioned through a different mechanism to CFX, synergy between compounds and CFX was analyzed. Drug synergy is the amplification of the effect of two drugs, such that their effect when co-administered is greater than the sum of their individual effects. Combination indexes (CIs) determined using the Chou-Talalay method quantitatively demonstrate synergism (CI < 1), additive effects (CI = 1), and antagonism (CI > 1). Constant combination ratio titrations were prepared as two-fold serial dilutions of CFX with test compounds in the ratio of their EC_50_ values for JE2 growth inhibition, such that both drugs exert approximately equal effects in the combination.^41^ A more precise method to determine EC_50_ values for individual compounds and combinations was used to increase accuracy for CI calculations. The OD_600_ was recorded over a 17 h period at 37 °C and the rate of the exponential growth phase used for dose–response analysis, rather than endpoint OD_600_ measurement, which resulted in decreased error in EC_50_ values (Figure S2). In combination, **10** exhibited synergy with CFX (CI = 0.70) and a dose-reduction index (DRI) of 2.8 (Figure 1C-E), agreeing with the observed compound potentiation by CFX in the JE2 cellular assays (Table 1). By contrast, **1** (CI = 1.0) and **5** (CI = 0.96) demonstrated an apparent additive relationship or weak synergy with CFX, respectively, consistent with their degree of potentiation (Table 1). These findings indicated that **10**, which we have given the synonym **IMP–1700**, is a highly active potentiator of bacterial DNA damage by CFX.

### 2.4 Investigation of the mechanism of action of IMP-1700

**Figure 2:**
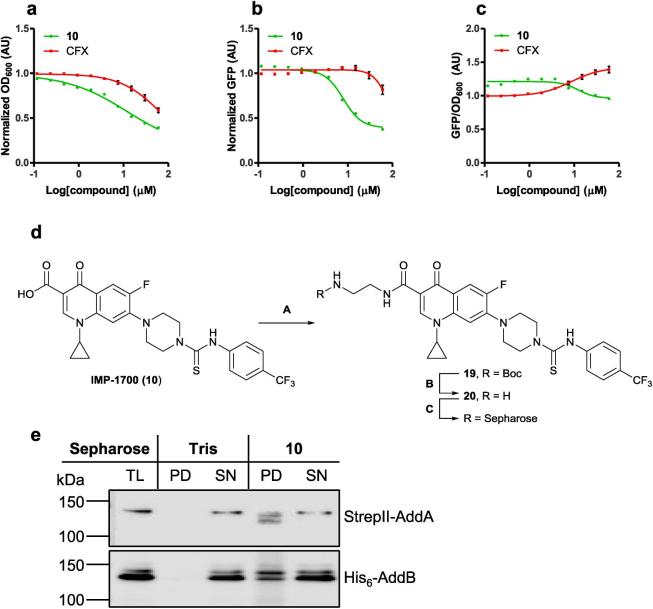
Mechanism of action of IMP-1700 (10). a) Growth inhibition of JE2 transformed to express GFP under control of the *recA* promoter, treated with 10 µM Ciprofloxacin (CFX) to induce DNA damage, plus titrations of 10 or CFX. b) GFP expression in response to titration of 10 or CFX. c) Normalized GFP fluorescence/cell, showing SOS activation increased with increased CFX but decreased with 10. Data represent mean ± SEM (n = 3 biological replicates). d) Synthesis of Sepharose resin functionalized with 10. Conditions (A) PyBOP, DIPEA, DMF, RT, overnight; (B) 4 M HCl in dioxane, RT, overnight; (C) NHS-Sepharose, 1:1 (v/v) DMSO:0.2 M NaHCO_3_, 0.5 M NaCl, pH 8.3, 4 °C, overnight. e) Pulldown from *E. coli* lysates co-expressing StrepII-AddA and His_6_-AddB, demonstrating Sepharose functionalized with 10 allows co-isolation of the complex (n = 2 biological replicates).

Having demonstrated a synergistic effect of **IMP-1700** (**10**) with CFX for killing of MRSA, further evidence was sought to support the conclusion that this occurred through inhibition of AddAB-mediated DNA repair. A SOS reporter system was generated by transformation of JE2 cells with a plasmid containing GFP under control of the *recA* promoter. Activation of the SOS response in these cells results in expression of GFP which can be monitored by fluorescence intensity. The SOS-response was activated via treatment of cells with CFX (10 µM) and the effect on cell number (OD_600_) and SOS activation (GFP fluorescence) recorded in response to titration of either **IMP-1700** (**10**) or CFX. Both compounds caused a decrease in cell number at low micromolar concentrations (Figure 2A); this increase in EC_50_ is postulated to result from the increased number of cells used for inoculation in this assay. Growth inhibition mediated by CFX was accompanied by an increase in normalized GFP fluorescence/cell (Figure 2B-C), consistent with the established MOA of CFX through DNA damage. In contrast, growth inhibition induced by **IMP-1700** (**10)** resulted in decreased normalized GFP fluorescence/cell (Figure 2B). This decrease is indicative of inhibition of the SOS response during cell death, and consistent with the proposed inhibition of AddAB-mediated DNA repair pathways. Finally, to provide additional evidence that inhibition of the bacterial DNA repair pathway by **IMP-1700** (**10**) occurred through inhibition of AddAB, a target pulldown was conducted. **IMP-1700** (**10**) was coupled with *N*-Boc-ethylenediamine to afford amide **19**, followed by removal of the Boc group in 4 M HCl/dioxane and purification using strong cation exchange resin. The resulting free amine **20** was coupled to *N*-hydroxysuccinimide (NHS)-activated Sepharose resin at 4 °C overnight (Figure 2C), and the remaining NHS sites quenched in Tris buffer. SoluBL21 competent *E. coli* were transformed with pET28b^+^ containing StrepII-tagged AddA and hexahistidine-tagged AddB, and expression induced overnight at 16 °C. Cells were lysed via sonication and the total lysate incubated with **IMP-1700** (**10**)**-**functionalized or control Sepharose beads which had been treated with DMSO then Tris-quenched. Beads were washed three times with lysis buffer and bound protein eluted with SDS-PAGE sample buffer, followed by PAGE separation. Western blotting for hexahistidine and StrepII tags indicated that both AddA and AddB subunits were pulled down by **IMP-1700** (**10**)**-**functionalized Sepharose, but not by the control Tris-Sepharose (Figure 2D). To further support the observed interaction between AddAB and **IMP-1700** (**10**), a structurally related negative control compound was prepared through reaction of CFX with phenyl isocyanate (**21**), which showed ∼100-fold weaker potentiation of CFX in MRSA growth assays, and did not inhibit the SOS response (Figure S3). **21** was attached to Sepharose resin as previously described, and pulldown experiments repeated which demonstrated no interaction between the inactive compound and AddAB (Figure S3).

## 3 Conclusions

The threat to global heath presented by the rise in antibiotic resistance means there is a critical need for small-molecule inhibitors of new targets with novel MOAs. The AddAB/RecBCD DNA repair complexes are attractive therapeutic targets with several potential advantages over conventional antibiotics. ML328 (**1**) was among the first small-molecule inhibitors of AddAB/RecBCD,^25^ although the molecule displayed only moderate potency.

Hypothesis-driven optimization of **1** was based on a proposed binding mode of intercalation into the DNA-enzyme complex. This resulted in CFX derivatives **7**–**12** which displayed increased activity compared to their PA analogues (Table 1). The lead inhibitor **IMP-1700** (**10**) sensitized MRSA to CFX with an EC_50_ of 5.9 ± 0.6 nM. This high cellular potency is encouraging for continued development towards a combination therapy to treat serious resistant infections. Whilst intercalation into the DNA-enzyme complex represents one putative means of small-molecule inhibition of AddAB/RecBCD, the protein complexes may present multiple other ligandable sites. For example, binding at the ATPase or Chi-recognition sites, blockage or labelling of the nucleophilic active site residue, or binding to allosteric sites. The availability of structures for AddAB (PDB 3U4Q)^42^ and RecBCD (PDB 1 W36)^43^ means direct binding site identification through co-crystallization may be possible with high affinity binders. Such structural information would greatly accelerate ligand development and significantly de-risk medicinal chemistry programs. Whilst MIC assays are highly relevant to clinical practice, the development of high-throughput biochemical assays for target functions will be required to support identification of clear SAR trends for target activity in the future. Such well-designed biochemical assays may also help decipher the mechanism of inhibition by **IMP-1700** (**10**).

The use of cellular assays for ligand development requires confirmation of a molecule’s MOA and demonstration of target engagement. The initial evidence presented here support **IMP-1700** (**10**) targeting of *S. aureus* DNA repair via the AddAB complex. Despite the presence of quinolone antibiotic motifs in **IMP-1700** and analogues, minimal activity was observed against purified DNA gyrase and topoisomerase *in vitro* (Figure 1A-B and Figure S1).

**IMP-1700** quantitatively synergized with CFX indicating a MOA that amplifies the effect of each compound (Figure 1C-E). In contrast, AddAB engagement by **1** may be weaker, resulting in a lack of quantitative synergy with CFX. The full analogue panel, including **IMP-1700** (**10**), did not potentiate alternative DNA-damaging agents MMC and hydrogen peroxide (Table S2). MMC and hydrogen peroxide result in alkylated and oxidized DNA bases, respectively, which are typically repaired by base excision or mismatch repair. Lack of potentiation of these mechanisms of DNA damage is therefore consistent with inhibitors targeting DSB repair. Investigation of **IMP-1700** (**10**) potentiation of other antibiotics is currently on-going in our laboratories. Growth inhibition by **IMP-1700** (**10**) also resulted in decreased SOS response, consistent with the proposed inhibition of AddAB-mediated SOS activation (Figure 2B). Cell growth was affected at lower **IMP-1700 (10)** concentrations than those required for SOS response inhibition (Figure 2A-B). A single DSB is lethal if not repaired by AddAB, whereas SOS response may be proportional to total cellular AddAB activity. Stabilization and arrest of the DNA-AddAB complex may render a single DSB irreparable if the complex cannot dissociate, thus accounting for the observation of lower growth inhibition EC_50_ than for SOS response inhibition. Interestingly, at low concentrations **IMP-1700 (10)** appears to increase the SOS response/cell, which is then decreased at higher concentrations (Figure 2C). This may indicate some degree of quinolone-like activity in a cellular context, potentially through hydrolysis or metabolism of the compound to CFX and may account for the observed single-agent toxicities in the absence of DNA damaging agents (Table 1). **IMP-1700** (**10**)-functionalized Sepharose successfully pulled down recombinant AddAB from lysates, whereas inactive control **21** did not (Figure 2D and Figure S3). This provides initial evidence supporting the proposed target, however, confirmation of target engagement in live cells along with profiling of the full scope of cellular targets is still required. The high potency of **IMP-1700** (**10**) serves as an optimal start point for the development of chemical probes containing biorthogonal handles for crosslinking to binding partners and ‘click chemistry’ functionalization for analysis. Such tools will be essential to aid further progress towards the clinic.

In summary, we present development of **IMP-1700** (**10**), a high potency potentiator of DNA damage in bacterial cells which is ∼160-fold more active than the best performing literature inhibitor. MRSA was sensitized to CFX by **IMP-1700** (**10**) at nanomolar concentrations, demonstrating potential as a combinational therapy to combat serious drug-resistant infections. **IMP-1700 (10)** will therefore serve as a useful tool molecule for continued development towards a clinically applicable compound. Collectively, this work supports validation of bacterial DNA repair as a promising drug target to address the pressing global threat of antibiotic resistance.
