# Supplementary material for: Identification of a potent small-molecule inhibitor of bacterial DNA repair that potentiates quinolone antibiotic activity in methicillin-resistant Staphylococcus aureus
Source: Bioorg Med Chem. 2019 Oct 15;27(20):114962. doi: 10.1016/j.bmc.2019.06.025 (PMC6892255; doi:10.1016/j.bmc.2019.06.025)
Supplement: Supplementary data 1 [file mmc1.pdf]

Supplementary information for

Identification of a potent small-molecule inhibitor of bacterial DNA repair that potentiates quinolone antibiotic activity in methicillin-resistant *Staphylococcus aureus*

Carine S. Q. Lim[a], Kam Pou Ha[b], Rebecca S. Clarke[b], Leigh-Anne Gavin[a], Declan T. Cook[a], Jennie A. Hutton[a], Charlotte L. Sutherell[a], Andrew M. Edwards[b], Lindsay E. Evans[a,b,*], Edward W. Tate[a,*] and Thomas Lanyon-Hogg[a,*]

[a] *Department of Chemistry, Molecular Sciences Research Hub, Imperial College London, London, UK, W12 0BZ*

[b] *MRC Centre for Molecular Bacteriology and Infection, Department of Medicine, Imperial College London, London, UK, SW7 2AZ*

* Corresponding authors: l.evans@imperial.ac.uk (L. E. Evans), e.tate@imperial.ac.uk (E. W. Tate) and t.lanyon-hogg@imperial.ac.uk (T. Lanyon-Hogg)

Table of Contents

1. **Supplementary data** 2

2. **Materials and methods** 8

2.1. **Biological and biochemical methods** 8

2.2. **Chemical synthesis** 11

2.3. **General information** 11

2.4. **General Procedures** 11

2.5. **NMR spectra of final compounds** 24

2.6. **NMR spectra of intermediate compounds** 47

3. **References** 50

1. Supplementary data

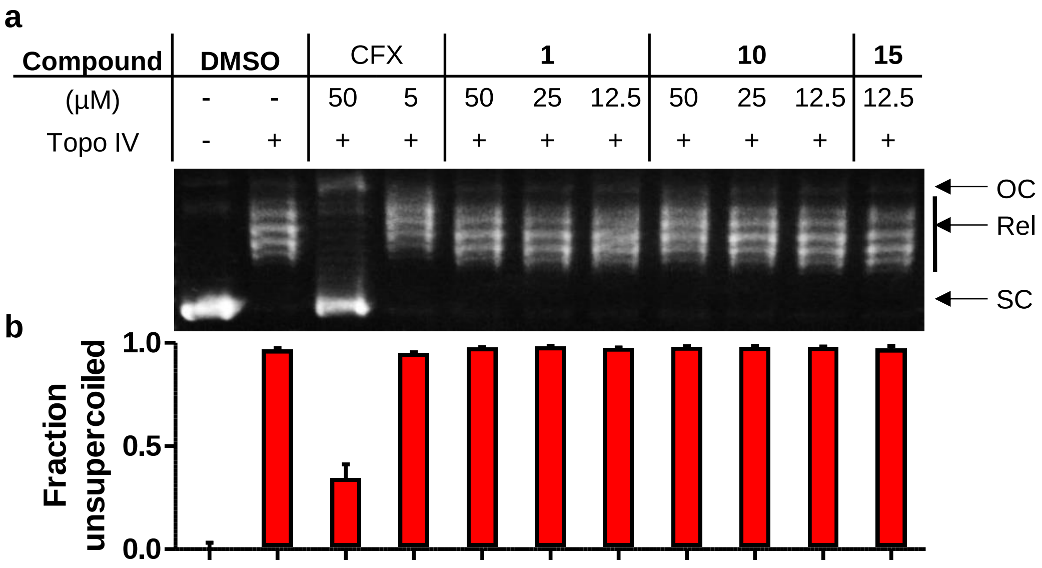

Figure S1. Compounds do not inhibit topoisomerase IV. a) Effect of compounds on *E. coli* topoisomerase IV relaxation of supercoiled pQE80L. Gyrase is inhibited by Ciprofloxacin (CFX), but not **1**, **5**, **10** or **15** (OC = open-circular DNA; Rel = relaxed DNA; SC = supercoiled DNA). b) Densitometry analysis of DNA supercoiling. Data represent mean ± SEM (n = 3 technical replicates).

CPD	End-point method		Growth rate method	
	EC50 (μM)	Dose-response curve	EC50 (μM)	Dose-response curve
**1**	6.9 ± 0.6 (8.7%)	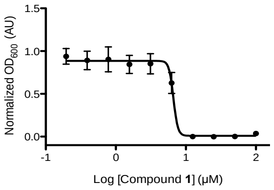	5.9 ± 0.2 (3.6%)	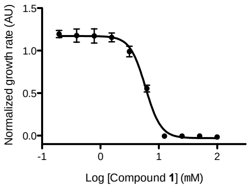
**5**	6.9 ± 0.1 (2.0%)	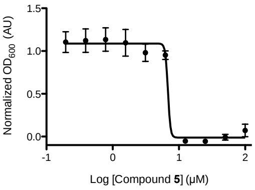	6.2 ± 0.1 (1.6%)	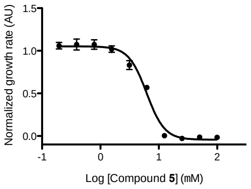
**10**	3.8 ± 0.5 (13%)	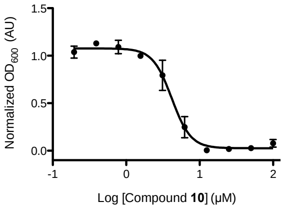	2.8 ± 0.2 (7.3%)	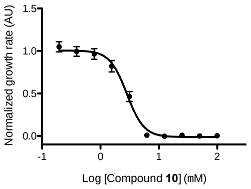
CFX	8.3 ± 1.9 (20%)	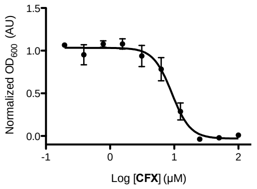	5.3 ± 0.3 (6.3%)	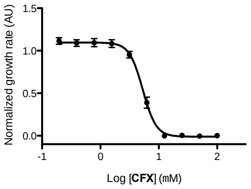

Figure S2. Comparison of quality of fit of dose-response curves for compounds 1, 5, 10 and CFX from end-point or growth rate cellular assays. Data represent mean ± SEM (n = 3 biological replicates). EC_{50} values determined by dose-response non-linear regression. Percentage error of mean in parentheses.

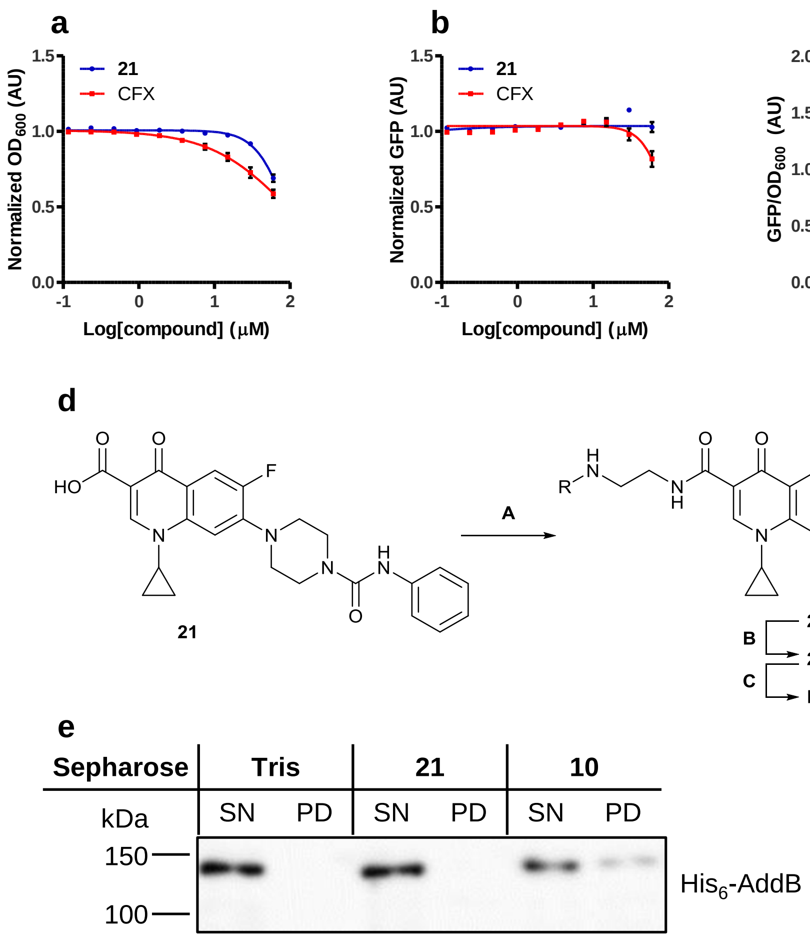

Figure S3. Inactive control derivitive 21 increases SOS response and does not pulldown AddAB. a) Growth inhibition of JE2 transformed to express GFP under control of the *recA* promoter, treated with 10 µM ciprofloxacin (CFX) to induce DNA damage, plus titrations of **21** or CFX. b) GFP expression in response to titration of **21** or CFX. c) Normalized GFP fluorescence/cell, showing SOS activation increased with increased CFX or **21**, in contrast to the response to **IMP-1700** (**10**). Data represent mean ± SEM (n = 3 biological replicates). d) Synthesis of Sepharose resin functionalized with **21**. Conditions (A) PyBOP, DIPEA, DMF, RT, overnight; (B) 4 M HCl in dioxane, RT, overnight; (C) NHS-Sepharose, 1:1 (v/v) DMSO:0.2 M $NaHCO_3$, 0.5 M NaCl, pH 8.3, 4 °C, overnight. e) Pulldown from *E. coli* lysate co-expressing StrepII-AddA and His_6-AddB, demonstrating Sepharose functionalized with **IMP-1700** (**10**) allows co-isolation of the complex whereas Sepharose functionaized with **21** does not.

Bacterial Strain		Description	Source or reference
E. coli	K-12 BW25113	Background strain used in the Keio Knockout Collection. Derivative of the F-, λ-, *E. coli* K-12 strain BD792. *ΔlacZ hsdR araBAD rhaBAD* (cannot metabolise arabinose or rhamnose). Sensitive to CFX.	Baba *et al.*, 2006[1]
E. coli	SoluBL21 (DE3)	Derivative of BL21 (DE3) for expression of challenging proteins.	Genlantis
S. aureus	SH1000	Functional *rsbU*$^{+}$ derivative of NCTC8325-4. Sensitive to CFX.	Horsburgh *et al.*, 2002[2]
S. aureus	USA300 JE2	A derivative of CA-MRSA USA300 LAC, cured of plasmids, sensitive to Erm. Resistant to CFX (*gyrA* Ser84Leu). *mecA arc* cluster *opp-3* cluster *seq2 sek2 lukS-PV lukF-PV sak chp*	Diep *et al.*, 2006[3]

Table S1. Bacterial strains used in this study.

Compound	EC$_{50}$ (μM)		
	Compound only	Compound + MMC (0.40 μM)	Compound + H_2O_2 (200 μM)
**1** (**ML328**)	6.9 ± 0.6	3.3 ± 0.6	6.5 ± 0.1
**2**	27 ± 0.4	21 ± 2.2	24 ± 3
**3**	2.7 ± 0.6	1.5 ± 0.1	2.4 ± 0.2
**4**	4.0 ± 0.3	2.9 ± 0.2	4.7 ± 0.9
**5**	6.9 ± 0.1	4.5 ± 1.2	8.4 ± 0.2
**6**	1.8 ± 0.1	1.5 ± 0.0	2.5 ± 0.4
**7**	3.7 ± 0.5	2.8 ± 0.4	5.5 ± 0.6
**8**	4.8 ± 0.9	3.4 ± 0.9	4.6 ± 0.4
**9**	3.5 ± 0.4	1.8 ± 0.3	2.6 ± 0.3
**10** (**IMP-1700**)	3.8 ± 0.5	1.4 ± 0.3	4.1 ± 0.6
**11**	2.8 ± 0.1	2.2 ± 0.6	3.2 ± 0.0
**12**	3.6 ± 0.4	0.8 ± 0.0	2.9 ± 0.8
**13**	>25[a]	>25[a]	>25[a]
**14**	>12.5[a]	>12.5[a]	>12.5[a]
**15**[b]	>12.5[a]	>12.5[a]	>12.5[a]

Table S2. Potency of compounds 1-15 as single-agents and in the presence of mitomycin-C (MMC, 0.40 μM) or H_2O_2 (200 μM) in MRSA (USA300 JE2). EC$_{50}$ values determined by dose-response non-linear regression. Data represent mean ± SEM (n = 3 biological replicates). [a]EC$_{50}$ not determined due to solubility limitations. [b]Urea instead of thiourea. Analogues highlighted possess the CFX core.

Compound	Compound EC_{50} (µM)		
	E. coli (K-12 BW25113)	*S. aureus* (SH1000)	*S. aureus* (USA 300 JE2)
**16**	0.30 ± 0.06	0.26 ± 0.04	3.9 ± 0.4
**17**	2.8 ± 0.4	1.6 ± 0.4	53 ± 13
**18**	>100	7.7 ± 1.2	9.7 ± 1.0
**19**	>100	>100	>100
**20**	47 ± 1	76 ± 5	78 ± 8
**MMC**	Not determined	Not determined	0.33 ± 0.01
**H_2O_2**	Not determined	Not determined	147 ± 11

Table S3. Potencies of compounds against *E. coli* (wild-type), *S. aureus* (SH1000) and methicillin-resistant *S. aureus* (MRSA, USA300 JE2). Potencies of compounds **16-20**, mitomycin-C (MMC) and H_2O_2 determined by dose-response non-linear regression of end-point OD_{600} measurements. Data represent mean ± SEM (n = 3 biological replicates) Analogues highlighted in grey are derivatives of **IMP-1700** (**10**).

2. Materials and methods

2.1. Biological and biochemical methods

Bacterial strains and culture conditions

Strains utilized in the present study are detailed in Table S1. All strains used were cultivated on Mueller Hinton Agar (MHA) and grown to stationary-phase in Mueller Hinton Broth (MHB) at 37 °C for 16 h, with shaking at 180 r.p.m.

Minimum inhibitory concentration (MIC) assays

MIC assays were performed in 96 well plates and MICs were determined in accordance with the broth microdilution protocol.[4] Strains grown to stationary-phase in MHB were adjusted to 5 x 10^5 colony-forming units (CFU)/mL in fresh MHB (100 µL/well) supplemented with DMSO to ensure a constant concentration of 0.5% (v/v) DMSO across all experiments. Where stated, assays were performed using MHB supplemented with CFX (9.4 µM), MMC (0.4 µM) or H_2O_2 (200 µM). Test compounds were prepared as two-fold serial dilutions. Cells were grown by static incubation at 37 °C in air for 18 h.[4] Following incubation, the MIC was taken as the lowest concentration with no visible bacterial growth, and the optical density at 600 nm (OD_{600}) was recorded. OD_{600} measurements were background corrected against no-inocula control and normalized to DMSO control for dose-response analysis. The EC_{50} value mean and SEM were calculated from individual EC_{50} values from three biological replicates.

DNA gyrase assay

E. coli DNA gyrase supercoiling assay (Inspiralis) was performed according to the manufacturer's instructions, ensuring a constant concentration of 1% (v/v) DMSO. Samples were separated on a 1% (w/v) agarose gel prepared with Tris/Borate/EDTA (TBE) buffer (1 M Tris-borate, 20 mM EDTA, pH 8.0). Electrophoresis was performed at 100 V until the bromophenol blue dye had run >6 cm to ensure resolution of relaxed and supercoiled DNA. The gel was stained with SYBR Safe DNA gel stain (Invitrogen, 1:10,000 dilution in TBE) for 30 min, and visualized on a Syngene Gel Doc system. Band intensity was quantified using ImageJ software and the fraction of supercoiled plasmid was determined as the ratio of the density of the supercoiled band to the total density. Values were background corrected against the no-gyrase control and normalized to DMSO control for dose-response analysis.

Topoisomerase IV assay

E. coli topoisomerase IV supercoiling relaxation assay (Inspiralis) was performed according to the manufacturer's instructions, ensuring a constant concentration of 1% (v/v) DMSO. Supercoiled pQE80L was prepared via plasmid purification and used as the substrate for the reaction. Reactions were analyzed as described for DNA gyrase assays.

Synergy experiments

Synergy experiments were performed as described for MIC assays. Two-fold serial dilutions of CFX, **1**, **5,** and **10** alone and in constant-ratio combinations (1:1, 1:2 and 1:1 (M/M) compound:CFX) were prepared, keeping the top final concentration at 100 μM. Incubation was carried out at 37 °C, with shaking at 220 r.p.m. OD_{600} was recorded every 30 min over a 17 h period. Measurements were background corrected against the no-inocula control before a growth curve of OD_{600} against time was constructed. The gradient of the exponential phase was obtained by linear regression. Gradients were normalized against the smallest and largest value within the respective data set prior to analysis with the CompuSyn software (http://www.combosyn.com/) to calculate combination index (CI) and dose-reduction index (DRI) values for each drug combination.

SOS reporter assay

JE2 transformed with SOS-inducible pCN34 *PrecA-gfp* reporter plasmid expressing GFP under the control of the *recA* promoter were grown to late stationary phase at 37 °C in MHB containing kanamycin (90 μg/mL). Stationary phase JE2 culture (1 mL) was centrifuged (17,000 × *g*, 2 min), the supernatant removed and cells resuspended in assay media (12.5 μM CFX in MHB, 1.5% (v/v) DMSO, 1 mL). Test inhibitors (**IMP-1700** (**10**), CFX, **21**, 66 μM) in assay media (360 μL) were two-fold serially diluted in assay media (180 μL), such that CFX concentration was maintained >12.5 μM and DMSO concentration maintained at 1.5% (v/v). JE2 culture (20 uL) was added to inhibitor solutions (180 μL) to a final cell density of 4 x 10^7 CFU/mL and samples incubated in a M200-PRO microplate reader (Tecan) at 37 °C with 700 rpm agitation for 17 h. GFP fluorescence intensity and OD_{600} were measured at 15 min intervals. Measurements were background corrected against no-inocula control and normalized to no-test inhibitor control at mid-exponential growth phase (4.5 h) for dose-response analysis.

Recombinant AddAB expression

SoluBL21 transformed with pET28b$^+$ AddAB expression plasmid coding for N-terminally StrepII-tagged AddA and N-terminally His_6-tagged AddB were selected by growth on Luria

agar (LA) supplemented with kanamycin (50 μg/mL). Liquid cultures were grown at 37 °C with shaking at 180 r.p.m until an OD_{600} of 0.5 was reached, at which point cultures were cooled to 16 °C. Protein expression was induced by addition of isopropyl β-D-thiogalactoside (1 mM), followed by incubation for 20 h at 16 °C with shaking. Cells were collected by centrifugation (4,000 × *g*, 5 min) and stored at -20 °C.

Cells were re-suspended in lysis buffer (50 mM Tris-HCl, 150 mM NaCl, pH 7.5) and lysed on ice by sonication (2 min, 5 s on-off cycle, 40% amplitude) using a Vibra-cell sonicator (Sonics). The lysate was cleared by centrifugation (17,000 × *g*, 30 min, 4 °C) prior to use.

Compound immobilization on Sepharose

Compounds **20** and **23** were coupled covalently to NHS-activated Sepharose 4 Fast Flow (GE Healthcare) according to the manufacturer's instructions. A final bead volume of 0.5 mL was suspended in a solution of **20** or **23** (10 μmol) in 1:1 (v/v) DMSO:coupling buffer (0.2 M $NaHCO_3$, 0.5 M NaCl, pH 8.3) (2 mL). For negative control beads, packed beads (0.5 mL) was suspended in 1:1 (v/v) DMSO:coupling buffer (2 mL). The reaction was incubated at 4 °C overnight. Afterward, any remaining free sites were blocked with three cycles of washing with buffer A (0.1 M Tris-HCl, pH 8.5) and buffer B (0.1 M sodium acetate, 0.5 M NaCl pH 4.5). Beads were re-suspended in water and stored at 4 °C prior to use.

Recombinant AddAB pulldown

Speharose beads (20 μL) were washed once with lysis buffer and incubated with cell lysate (200 μL) for 30 min at 4 °C. Following incubation, mixtures were centrifuged (1,000 × *g*, 2 min, 4 °C) and the supernatant was removed, then beads were washed twice with lysis buffer (200 μL). 2 × Laemmli sample buffer (25 μL, Bio-Rad) was added to the beads and samples incubated at 95 °C for 5 min, centrifuged (1,000 × *g*, 2 min), then separated by SDS-PAGE on a 12%, 14% or 4-20% gel (SERVA*Gel*). Proteins were then transferred onto polyvinylidene difluoride (PVDF) membranes using an iBlot transfer device (Invitrogen) following the manufacturer's instructions. Membranes were blocked with 3% (w/v) bovine serum albumin (BSA) in Tris Buffer Saline supplemented with 0.1% (v/v) Tween-20 (TBS-T) for 1 h at RT, incubated with anti-His-HRP monoclonal antibody (Invitrogen, 1: 2,000 in 3% (w/v) BSA in TBS-T) or StrepTactin-HRP conjugate (Bio-Rad, 1: 5,000 in 3% (w/v) BSA in TBS-T) for 1 h at 4 °C, then washed three times with TBS-T. Membranes were visualized with Luminata Crescendo Western HRP substrate (Millipore) for chemiluminescence imaging.

2.2. Chemical synthesis

2.3. General information

Unless otherwise stated, materials were purchased from commercial suppliers (Fisher Scientific, Sigma Aldrich, Fluorochem) and used as received. Analytical thin-layer chromatography (TLC) was performed using E. Merck silica gel 60 F254 pre-coated plates (0.25 mm) and visualized under ultraviolet light (254 nm) or by ninhydrin staining for amine intermediates. Purification by column chromatography was carried out using Merck Flash Silica Gel 60 (230-400 mesh). Silica-bound cation exchanger absorbant propylsulfonic acid (SCX-2) was obtained from Biotage.

^{1}H NMR and ^{13}C NMR spectra were recorded at room temperature at 500 MHz and 125 MHz respectively, or 400 MHz and 100 MHz respectively. Chemical shifts are reported in the standard δ notation of parts per million (ppm) using the peak of the residual solvent proton signals as internal reference. Coupling constants (*J*) are reported in hertz (Hz), averaged for interacting protons. A Biotage Initiator was used for the microwave-assisted reactions. LCMS analyses of compounds were carried out by the Imperial Mass Spectrometry Service. Purity of all test compounds determined to be ≥ 95% by UV analysis (5-98% MeCN gradient in H_2O, supplemented with 0.1% (v/v) formic acid).

Ultrapure water was obtained using a MilliQ® Millipore purification system. Chemiluminescence was recorded with the use of a LAS-4000 Imaging System (GE Healthcare). OD_{600} was recorded on a Tecan Infinite M200 Pro plate reader. Compounds were prepared as DMSO stocks for biological experiments. Ciprofloxacin HCl was dissolved in water before filter sterilization. All compounds were stored at -20 ^{o}C and thawed on the day of use. Quantification of band intensities by densitometry was performed using ImageJ. Regression analysis was performed with GraphPad Prism 5, a four-parameter fit was used to carry out all dose-response nonlinear regressions.

2.4. General Procedures

General Procedure A[5]: Thiourea/urea formation from quinolone analogues. To an oven-dried round bottom flask flushed with argon for 10 min was added the respective quinolone analogue (250 mg, 1 eq), dry DMF (40 mL), $NaHCO_3$ (1.2 eq) and the corresponding isothiocyanate or isocyanate (1 eq). The reaction flask was once again flushed with Argon then

allowed to stir at RT overnight. Afterward, the reaction was quenched by addition of saturated NH_4Cl solution (25 mL) then extracted with EtOAc. The organic phase was washed with 5% w/v LiCl (aq.), water and brine. The solvent was removed by vacuum and the residue was washed with water, followed by MeOH, and isolated by suction filtration. If precipitation occurred in the organic phase, the solid was collected by vacuum filtration, then washed with water and MeOH.

General Procedure B[6]: Microwave-assisted amination of 7-chloro-1-cyclopropyl-6-fluoro-4-oxo-1,4-dihydroquinoline-3-carboxylic acid (Q1a). A mixture of 7-chloro-1-cyclopropyl-6-fluoro-4-oxo-1,4-dihydroquinoline-3-carboxylic acid (**Q1a**) (500 mg, 1.8 mmol, 1 eq), the corresponding cyclic amine (8.9 mmol, 5 eq) and DMSO (3.6 mL) was heated by microwave irradiation at 115 °C for 3 h. The resultant mixture was concentrated by freeze drying, and to the residue was added EtOAc (30 mL). The precipitate was isolated by vacuum filtration and washed with EtOAc, water and Et_2O to afford the Boc-protected aminated quinolone.

General Procedure C: Amination of 1-cyclopropyl-6,7-difluoro-4-oxo-1,4-dihydroquinoline-3-carboxylic acid (Q1b). The corresponding amine (2.07 mmol, 1.1 eq) was added to a suspension of 1-cyclopropyl-6,7-difluoro-4-oxo-1,4-dihydroquinoline-3-carboxylic acid (**Q1b**) (500 mg, 1.89 mmol, 1 eq) in pyridine (10 mL). The resulting mixture was heated at reflux at 120 °C for 16 h. The resultant mixture was concentrated under reduced pressure. The crude product was isolated by vacuum filtration and washed with EtOAc, water and Et_2O to afford the Boc-protected aminated quinolone.

General Procedure D: Boc-deprotection by HCl in dioxane. The amine obtained from general procedure B or C (1 eq) was suspended in 4 M HCl in dioxane (40 eq) then allowed to stir at RT overnight. The solvent was removed under reduced pressure to afford the product.

8-Ethyl-5-oxo-2-(4-((3-(trifluoromethyl)phenyl)carbamothioyl)piperazin-1-yl)-5,8-dihydropyrido[2,3-*d*]pyrimidine-6-carboxylic acid (1). Thiourea (**1**) was obtained from pipemidic acid (PA) (250 mg, 0.824 mmol) and 3-(trifluoromethyl)phenyl isothiocyanate (167 mg, 0.824 mmol), using general procedure A, as white solid (315 mg, 74%). R_f = 0.50 (SiO_2; DCM:MeOH, 90:10); ^{1}H NMR (400 MHz, DMSO-d_6) δ 14.79 (s, 1H), 10.07 (s, 1H),

9.25 (s, 1H), 8.99 (s, 1H), 7.82 (s, 1H), 7.74 (d, ^3J = 8.0 Hz, 1H), 7.52 (app. t, ^3J = 8.0 Hz, 1H), 7.42 (d, ^3J = 8.0 Hz, 1H), 4.43 (q, ^3J = 7.2 Hz, 2H), 4.11 (m, 8H), 1.38 (t, ^3J = 7.2 Hz, 3H); ^{13}C NMR (101 MHz, DMSO-d_6) δ 181.2, 177.2, 165.2, 160.6, 160.2, 155.0, 150.8, 141.7, 129.1, 128.71, 128.65 (q, $^2J_{C\text{-}F}$ = 31 Hz), 124.1 (q, $^1J_{C\text{-}F}$ = 274 Hz), 121.2, 120.6, 109.6, 108.8, 47.4, 47.0, 46.0, 43.3, 43.1, 14.4; LC-MS rt = 2.6 min; *m/z* 507 ([M+H]$^+$); HRMS found [M+H]$^+$ 507.1417, $C_{22}H_{22}F_3N_6O_3S$ requires 507.1421.

8-Ethyl-2-(4-((3-fluorophenyl)carbamothioyl)piperazin-1-yl)-5-oxo-5,8-dihydropyrido[2,3-*d*]pyrimidine-6-carboxylic acid (2). Thiourea (**2**) was obtained from PA (250 mg, 0.824 mmol) and 3-fluorophenyl isothiocyanate (99 µL, 0.824 mmol), using general procedure A, as a white solid (171 mg, 45%). R_f = 0.55 (SiO_2; DCM:MeOH, 90:10); ^{1}H NMR (400 MHz, DMSO-d_6) δ 14.78 (s, 1H), 9.53 (s, 1H), 9.25 (s, 1H), 8.99 (s, 1H), 7.33 (td, ^3J = 8.0, $^4J_{H\text{-}F}$ = 8.0, 1H), 7.28 (dt, $^2J_{H\text{-}F}$ = 11.2, ^4J = 4.0 Hz, 1H), 7.17 (ddd, ^3J = 8.0, ^4J = 4.0, $^5J_{H\text{-}F}$ = 4.0 Hz, 1H), 6.94 (ddd, $^3J_{H\text{-}F}$ = 8.0, ^3J = 8.0, ^4J = 4.0 Hz, 1H), 4.43 (q, ^3J = 7.1 Hz, 2H), 4.11 – 4.08 (m, 6H), 4.01 (bs, 2H), 1.38 (t, ^3J = 7.1 Hz, 3H); ^{13}C NMR (101 MHz, DMSO-d_6) δ 181.3, 177.2, 165.2, 161.6 (d, $^1J_{C\text{-}F}$ =242 Hz), 160.6, 160.2, 155.0, 150.8, 142.7 (d, $^3J_{C\text{-}F}$ = 11 Hz), 129.5 (d, $^3J_{C\text{-}F}$ = 9 Hz), 120.5, 111.6 (d, $^2J_{C\text{-}F}$ = 24 Hz), 110.7 (d, $^2J_{C\text{-}F}$ = 21 Hz), 109.6, 108.7, 47.5, 47.0, 45.9, 43.3, 43.1, 14.4; LC-MS rt = 2.3 min; *m/z* 457 ([M+H]$^+$); HRMS found [M+H]$^+$ 457.1453, $C_{21}H_{22}FN_6O_3S$ requires 457.1453.

2-(4-((3,5-Bis(trifluoromethyl)phenyl)carbamothioyl)piperazin-1-yl)-8-ethyl-5-oxo-5,8-dihydropyrido[2,3-*d*]pyrimidine-6-carboxylic acid (3). Thiourea (**3**) was obtained from PA (250 mg, 0.824 mmol) and 3,5-bis(trifluoromethyl)phenyl isothiocyanate (151 µL, 0.824 mmol), using general procedure A, as a white solid (400 mg, 84%). R_f = 0.58 (SiO_2; DCM:MeOH, 90:10); ^{1}H NMR (400 MHz, DMSO-d_6) δ 14.79 (s, 1H), 9.89 (s, 1H), 9.27 (s, 1H), 9.01 (s, 1H), 8.14 (s, 2H), 7.79 (s, 1H), 4.44 (q, ^3J = 7.1 Hz, 2H), 4.17 (bs, 2H), 4.13 (bs, 4H), 4.05 (bs, 2H), 1.39 (t, ^3J = 7.1 Hz, 3H); ^{13}C NMR (101 MHz, DMSO-d_6) δ 180.8, 177.3, 165.2, 160.7, 160.2, 155.1, 150.9, 143.0, 129.8 (q, $^2J_{C\text{-}F}$ = 30 Hz), 124.5, 123.3 (q, $^1J_{C\text{-}F}$ = 275 Hz), 116.8, 109.7, 108.9, 47.6, 47.1, 46.0, 43.3, 43.1, 14.4; LC-MS rt = 2.9 min; *m/z* 575 ([M+H]$^+$); HRMS found [M+H]$^+$ 575.1328, $C_{23}H_{21}F_6N_6O_3S$ requires 575.1295.

8-Ethyl-5-oxo-2-(4-((4-(trifluoromethyl)phenyl)carbamothioyl)piperazin-1-yl)-5,8-dihydropyrido[2,3-*d*]pyrimidine-6-carboxylic acid (4). Thiourea (**4**) was obtained from PA (250 mg, 0.824 mmol) and 4-(trifluoromethyl)phenyl isothiocyanate (167 mg, 0.824 mmol),

using general procedure A, as white solid (245 mg, 55%). R_f = 0.50 (SiO_2; DCM:MeOH, 90:10); ^{1}H NMR (400 MHz, DMSO-d_6) δ 14.78 (s, 1H), 9.69 (s, 1H), 9.25 (s, 1H), 8.99 (s, 1H), 7.66 (d, ^3J = 8.6 Hz, 2H), 7.58 (d, ^3J = 8.6 Hz, 2H), 4.43 (q, ^3J = 7.1 Hz, 2H), 4.13 (bs, 2H), 4.10 (bs, 4H), 4.04 (bs, 2H), 1.38 (t, ^3J = 7.1 Hz, 3H); ^{13}C NMR (101 MHz, DMSO-d_6) δ 181.3, 177.2, 165.2, 160.6, 160.2, 155.0, 150.8, 144.8, 125.1, 124.2, 109.6, 108.8, 47.6, 47.2, 46.0, 43.3, 43.1, 14.4[I]; LC-MS rt = 2.6 min; *m/z* 507 ($[M+H]^+$); HRMS found $[M+H]^+$ 507.1433, $C_{22}H_{22}F_3N_6O_3S$ requires 507.1421.

2-(4-((3-Chlorophenyl)carbamothioyl)piperazin-1-yl)-8-ethyl-5-oxo-5,8-dihydropyrido[2,3-*d*]pyrimidine-6-carboxylic acid (5). Thiourea (**5**) was obtained from PA (250 mg, 0.824 mmol) and 3-chlorophenyl isothiocyanate (108 µL, 0.824 mmol), using general procedure A, as a white solid (145 mg, 37%). R_f = 0.48 (SiO_2; DCM:MeOH, 90:10); ^{1}H NMR (400 MHz, DMSO-d_6) δ 14.78 (s, 1H), 9.52 (s, 1H), 9.24 (s, 1H), 8.98 (s, 1H), 7.49 – 7.46 (m, 1H), 7.35 – 7.31 (m, 2H), 7.17 (app dt, ^3J = 8.0, ^4J =4.0 Hz, 1H), 4.42 (q, ^3J = 7.0 Hz, 2H), 4.11 (bs, 6H), 4.02 (bs, 2H), 1.38 (t, ^3J = 7.0 Hz, 3H); ^{13}C NMR (126 MHz, DMSO-d_6) δ 181.2, 177.2, 165.3, 160.7, 160.2, 155.1, 150.8, 142.5, 132.1, 129.6, 124.6, 124.0, 123.4, 109.7, 108.8, 47.5, 47.0, 46.0, 43.3, 43.1, 14.4; LC-MS rt = 2.6 min; *m/z* 473 ($[M+H]^+$); HRMS found $[M+H]^+$ 473.1162, $C_{21}H_{22}ClN_6O_3S$ requires 473.1157.

2-(4-((3,5-Dichlorophenyl)carbamothioyl)piperazin-1-yl)-8-ethyl-5-oxo-5,8-dihydropyrido[2,3-*d*]pyrimidine-6-carboxylic acid (6). Thiourea (**6**) was obtained from PA (250 mg, 0.824 mmol) and 3,5-dichlorophenyl isothiocyanate (168 mg, 0.824 mmol), using general procedure A, as a white solid (296 mg, 71%). R_f = 0.52 (SiO_2; DCM:MeOH, 90:10); ^{1}H NMR (400 MHz, DMSO-d_6) δ 14.78 (s, 1H), 9.60 (s, 1H), 9.26 (s, 1H), 8.99 (s, 1H), 7.49 (d, ^4J = 1.9 Hz, 2H), 7.32 (t, ^4J = 1.9 Hz, 1H), 4.43 (q, ^3J = 7.1 Hz, 2H), 4.11 (bs, 6H), 4.02 (bs, 2H), 1.38 (t, ^3J = 7.1 Hz, 3H); ^{13}C NMR (101 MHz, DMSO-d_6) δ 180.9, 177.2, 165.2, 160.6, 160.2, 155.0, 150.8, 143.5, 133.0, 123.2, 122.8, 109.6, 108.8, 47.6, 47.1, 46.0, 43.3, 43.1, 14.4; LC-MS rt = 2.9 min; *m/z* 507 ($[M+H]^+$); HRMS found $[M+H]^+$ 507.0784, $C_{21}H_{21}Cl_2N_6O_3S$ requires 507.0767.

[I] **C**-CF_3 and –**C**F_3 not observed in the C^{13} NMR spectra

1-Cyclopropyl-6-fluoro-4-oxo-7-(4-((3-(trifluoromethyl)phenyl)carbamothioyl)piperazin-1-yl)-1,4-dihydroquinoline-3-carboxylic acid (7). Thiourea (**7**) was obtained from ciprofloxacin (CFX) (250 mg, 0.755 mmol) and 3-(trifluoromethyl)phenyl isothiocyanate (115 µL, 0.755 mmol), using general procedure A, as a white solid (210 mg, 51%). R_f = 0.56 (SiO_2; DCM:MeOH, 90:10); ^{1}H NMR (400 MHz, DMSO-d_6) δ 15.22 (s, 1H), 9.76 (s, 1H), 8.67 (s, 1H), 7.94 (d, $^3J_{H\text{-}F}$ = 13.2 Hz, 1H), 7.75 (s, 1H), 7.69 (d, ^3J = 8.3 Hz, 1H), 7.59 (d, $^4J_{H\text{-}F}$ = 7.5 Hz, 1H), 7.54 (t, ^3J = 7.9 Hz, 1H), 7.45 (d, ^3J = 8.0 Hz, 1H), 4.19 (t, ^3J = 4 0 Hz, 4H), 3.83 (tt, ^3J = 7.2, 4.0 Hz, 1H), 3.51 (t, ^3J = 4 0 Hz, 4H), 1.37 – 1.28 (m, 2H), 1.24 – 1.16 (m, 2H); ^{13}C NMR (101 MHz, DMSO-d_6) δ 181.2, 176.3, 165.9, 152.8 (d, $^1J_{C\text{-}F}$ = 250 Hz), 148.0, 144.6 (d, $^2J_{C\text{-}F}$ = 10 Hz), 141.8, 139.2, 129.1, 128.7 (q, $^2J_{C\text{-}F}$ = 32 Hz), 128.7, 124.1 (q, $^1J_{C\text{-}F}$ = 274 Hz), 121.2 (app d, $^3J_{C\text{-}F}$ = 4 Hz), 120.5 (app d, $^3J_{C\text{-}F}$ = 3 Hz), 118.6 (d, $^3J_{C\text{-}F}$ = 7 Hz), 111.0 (d, $^2J_{C\text{-}F}$ = 23 Hz), 106.7, 106.2, 48.8, 47.6, 35.9, 7.6; LC-MS rt = 2.6 min; *m/z* 535 ($[M+H]^+$); HRMS found $[M+H]^+$ 535.1426, $C_{25}H_{23}F_4N_4O_3S$ requires 535.1422.

1-Cyclopropyl-6-fluoro-7-(4-((3-fluorophenyl)carbamothioyl)piperazin-1-yl)-4-oxo-1,4-dihydroquinoline-3-carboxylic acid (8). Thiourea (**8**) was obtained from CFX (250 mg, 0.755 mmol) and 3-fluorophenyl isothiocyanate (91 µL, 0.755 mmol), using general procedure A, as a white solid (310 mg, 85%). R_f = 0.49 (SiO_2; DCM:MeOH, 90:10); ^{1}H NMR (400 MHz, DMSO-d_6) δ 15.20 (s, 1H), 9.57 (s, 1H), 8.65 (s, 1H), 7.91 (d, $^3J_{H\text{-}F}$ = 13.2 Hz, 1H), 7.57 (d, $^4J_{H\text{-}F}$ = 7.4 Hz, 1H), 7.33 (app td, ^3J = 8.0, ^4J = 8.0, 1H), 7.26 (dt, $^3J_{H\text{-}F}$ = 11.2, ^4J = 4.0 Hz, 1H), 7.16 (ddd, ^3J = 8.0, ^4J =4.0, $^5J_{H\text{-}F}$ =4.0 Hz, 1H), 6.93 (ddd, $^3J_{H\text{-}F}$ = 8.0, ^3J = 8.0, ^4J = 4.0 Hz, 1H), 4.15 (t, ^3J = 4.0 Hz, 4H), 3.81 (tt, ^3J = 4.0, 4.0 Hz, 1H), 3.49 (t, ^3J = 4.0 Hz, 4H), 1.33 – 1.31 (m, 2H), 1.19 – 1.18 (m, 2H); ^{13}C NMR (101 MHz, DMSO-d_6) δ 181.3, 176.3, 165.9, 161.6 (d, $^1J_{C\text{-}F}$ = 242 Hz), 152.7 (d, $^1J_{C\text{-}F}$ = 250 Hz), 148.0, 144.6 (d, $^3J_{C\text{-}F}$ = 9 Hz), 142.8 (d, $^2J_{C\text{-}F}$ = 11 Hz), 139.2, 129.5 (d, $^3J_{C\text{-}F}$ = 10 Hz), 120.5, 118.6 (d, $^3J_{C\text{-}F}$ = 7 Hz), 111.6 (d, $^2J_{C\text{-}F}$ = 25 Hz), 111.0 (d, $^2J_{C\text{-}F}$ = 23 Hz), 110.8 (d, $^2J_{C\text{-}F}$ = 20 Hz), 106.7, 106.2, 48.8, 47.6, 35.9, 7.6; LC-MS rt = 2.5 min; *m/z* 485 ($[M+H]^+$); HRMS found $[M+H]^+$ 485.1470, $C_{24}H_{23}F_2N_4O_3S$ requires 485.1453.

7-(4-((3,5-Bis(trifluoromethyl)phenyl)carbamothioyl)piperazin-1-yl)-1-cyclopropyl-6-fluoro-4-oxo-1,4-dihydroquinoline-3-carboxylic acid (9). Thiourea (**9**) was obtained from CFX (250 mg, 0.755 mmol) and 3,5-bis(trifluoromethyl)phenyl isothiocyanate (138 µL, 0.755 mmol), using general procedure A, as a white solid (367 mg, 79%). R_f = 0.45 (SiO_2;

DCM:MeOH, 90:10); ^{1}H NMR (400 MHz, DMSO-d_6) δ 15.22 (s, 1H), 9.95 (s, 1H), 8.68 (s, 1H), 8.13 (s, 2H), 7.95 (d, $^3J_{H-F}$ = 13.1 Hz, 1H), 7.80 (s, 1H), 7.59 (d, $^4J_{H-F}$ = 7.4 Hz, 1H), 4.21 (bs, 4H), 3.83 (bs, 1H), 3.53 (bs, 4H), 1.35 – 1.30 (m, 2H), 1.20 (bs, 2H); ^{13}C NMR (101 MHz, DMSO-d_6) δ 180.7, 176.4, 165.9, 152.7 (d, $^1J_{C-F}$ = 248 Hz), 148.1, 144.5 (d, $^2J_{C-F}$ = 10 Hz), 139.2, 129.9, 129.6 (app d, $^2J_{C-F}$ = 33 Hz), 124.4, 123.3 (app d, $^1J_{C-F}$ = 273 Hz), 118.6 (d, $^3J_{C-F}$ = 8 Hz), 116.7, 111.1 (d, $^2J_{C-F}$ = 20 Hz), 106.7, 106.2, 48.7, 47.7, 35.9, 7.6; LC-MS rt = 3.0 min; *m/z* 603 ([M+H]$^+$); HRMS found [M+H]$^+$ 603.1297, $C_{26}H_{22}F_7N_4O_3S$ requires 603.1295.

1-Cyclopropyl-6-fluoro-4-oxo-7-(4-((4-(trifluoromethyl)phenyl)carbamothioyl)piperazin-1-yl)-1,4-dihydroquinoline-3-carboxylic acid (10). Thiourea (**10**) was obtained from CFX (250 mg, 0.755 mmol) and 4-(trifluoromethyl)phenyl isothiocyanate (153 mg, 0.755 mmol), using general procedure A, as a white solid (259 mg, 64%). R_f = 0.43 (SiO_2; DCM:MeOH, 90:10); ^{1}H NMR (400 MHz, DMSO-d_6) δ 15.21 (s, 1H), 9.74 (s, 1H), 8.67 (s, 1H), 7.95 (d, $^3J_{H-F}$ = 13.1 Hz, 1H), 7.67 (d, ^3J = 8.1 Hz, 2H), 7.59 (d, ^3J = 8.1 Hz, 2H), 7.57 (s, 1H), 4.17 (t, ^3J = 4.0 Hz, 4H), 3.83 (bs, 1H), 3.51 (t, ^3J = 4.0 Hz, 4H), 1.35 – 1.30 (m, 2H), 1.22 – 1.18 (m, 2H); ^{13}C NMR (101 MHz, DMSO-d_6) δ 181.3, 176.3, 165.9, 152.86 (d, $^1J_{C-F}$ = 249 Hz), 148.1, 144.9, 144.6 (d, $^2J_{C-F}$ = 10 Hz), 139.2, 125.1 (d, $^3J_{C-F}$ = 4 Hz), 124.5 (q, $^1J_{C-F}$ = 273 Hz), 124.2, 123.7 (q, $^2J_{C-F}$ = 31 Hz), 118.6 (d, $^3J_{C-F}$ = 6 Hz), 111.0 (d, $^2J_{C-F}$ = 23 Hz), 106.7, 106.3, 48.8, 47.8, 35.9, 7.6; LC-MS rt = 2.7 min; *m/z* 535 ([M+H]$^+$); HRMS found [M+H]$^+$ 535.1429, $C_{25}H_{23}F_4N_4O_3S$ requires 535.1422.

7-(4-((3-Chlorophenyl)carbamothioyl)piperazin-1-yl)-1-cyclopropyl-6-fluoro-4-oxo-1,4-dihydroquinoline-3-carboxylic acid (11). Thiourea (**11**) was obtained from CFX (250 mg, 0.755 mmol) and 3-chlorophenyl isothiocyanate (99 µL, 0.755 mmol), using general procedure A, as a white solid (297 mg, 79%). R_f = 0.74 (SiO_2; DCM:MeOH, 90:10); ^{1}H NMR (400 MHz, DMSO-d_6) δ 15.21 (s, 1H), 9.57 (s, 1H), 8.67 (s, 1H), 7.94 (d, $^3J_{H-F}$ = 13.2 Hz, 1H), 7.58 (d, $^4J_{H-F}$ = 7.5 Hz, 1H), 7.47 (app t, ^4J = 4.0 Hz, 1H), 7.37 – 7.29 (m, 2H), 7.17 (app dt, ^3J = 8.0, ^4J =4.0 Hz, 1H), 4.16 (t, ^3J = 5.9 Hz, 4H), 3.83 (tt, ^3J = 4.0, ^3J =4.0 Hz, 1H), 3.49 (t, ^3J = 5.9 Hz, 4H), 1.37 – 1.29 (m, 2H), 1.20 (m, 2H); ^{13}C NMR (126 MHz, DMSO-d_6) δ 181.2, 176.4, 165.9, 152.8 (d, $^1J_{C-F}$ = 249 Hz), 148.1, 144.6 (d, $^2J_{C-F}$ = 11 Hz), 142.5, 139.2, 132.1, 129.6, 124.6, 124.0, 123.4, 118.6 (d, $^1J_{C-F}$ = 8 Hz), 111.0 (d, $^2J_{C-F}$ = 23 Hz), 106.7, 106.3, 48.8, 47.6, 35.9, 7.6; LC-MS rt = 2.6 min; *m/z* 501 ([M+H]$^+$); HRMS found [M+H]$^+$ 501.1170, $C_{24}H_{23}ClFN_4O_3S$ requires 501.1158.

1-Cyclopropyl-7-(4-((3,5-dichlorophenyl)carbamothioyl)piperazin-1-yl)-6-fluoro-4-oxo-1,4-dihydroquinoline-3-carboxylic acid (12). Thiourea (**12**) was obtained from CFX (250 mg, 0.755 mmol) and 3,5-dichlorophenyl isothiocyanate (154 mg, 0.755 mmol), using general procedure A, as a white solid (363 mg, 90%). R_f = 0.51 (SiO_2; DCM:MeOH, 90:10); ^{1}H NMR (400 MHz, DMSO-d_6) δ 15.20 (s, 1H), 9.65 (s, 1H), 8.67 (s, 1H), 7.93 (d, $^3J_{H-F}$ = 13.2 Hz, 1H), 7.57 (d, $^4J_{H-F}$ = 7.4 Hz, 1H), 7.48 (d, ^4J = 1.9 Hz, 2H), 7.32 (t, ^4J = 1.9 Hz, 1H), 4.16 (t, ^3J = 4.9 Hz, 4H), 3.82 (bs, 1H), 3.50 (t, ^3J = 4.9 Hz, 4H), 1.38 – 1.26 (m, 2H), 1.26 – 1.13 (m, 2H); ^{13}C NMR (101 MHz, DMSO-d_6) δ 180.8, 176.3, 165.9, 152.7 (d, $^1J_{C-F}$ = 250 Hz), 148.1, 144.5 (d, $^2J_{C-F}$ = 11 Hz), 143.5, 139.2, 133.1, 123.2, 122.8, 118.6 (d, $^3J_{C-F}$ = 7 Hz), 111.0 (d, $^2J_{C-F}$ = 23 Hz) , 106.7, 106.2, 48.7, 47.7, 35.9, 7.6; LC-MS rt = 2.9 min; *m/z* 535 ($[M+H]^+$); HRMS found $[M+H]^+$ 535.0787, $C_{24}H_{22}Cl_2FN_4O_3S$ requires 535.0768.

1-Cyclopropyl-6-fluoro-7-(4-((3-nitrophenyl)carbamothioyl)piperazin-1-yl)-4-oxo-1,4-dihydroquinoline-3-carboxylic acid (13). Thiourea (**13**) was obtained from CFX (250 mg, 0.755 mmol) and 3-nitrophenyl isothiocyanate (136 mg, 0.755 mmol), using general procedure A, as a white solid (331 mg, 86%). R_f = 0.51 (SiO_2; DCM:MeOH, 90:10); ^{1}H NMR (400 MHz, DMSO-d_6) δ 15.21 (s, 1H), 9.83 (s, 1H), 8.68 (s, 1H), 8.31 (t, ^4J = 4.0 Hz, 1H), 7.98 – 7.97 (m, 1H), 7.96 – 7.93 (m, 1H), 7.85 (app dt, ^3J = 8.1, ^4J = 4.0 Hz, 1H), 7.63 – 7.57 (m, 2H), 4.20 (t, ^3J = 4.9 Hz, 4H), 3.83 (tt, ^4J = 4.0, ^4J = 4.0 Hz, 1H), 3.52 (t, ^3J = 4.9 Hz, 4H), 1.34 – 1.30 (m, 2H), 1.22 – 1.18 (m, 2H); ^{13}C NMR (126 MHz, DMSO-d_6) δ 181.1, 176.4, 165.9, 152.8 (d, $^1J_{C-F}$ = 249 Hz), 148.1, 147.3, 144.6 (d, $^2J_{C-F}$ = 9 Hz), 142.3, 139.2, 130.9, 129.2, 118.9, 118.7, 118.6, 111.1 (d, $^2J_{C-F}$ = 24 Hz), 106.7, 106.3, 48.8, 47.6, 35.9, 7.6; LC-MS rt = 2.5 min; *m/z* 512 ($[M+H]^+$); HRMS found $[M+H]^+$ 512.1404, $C_{24}H_{23}FN_5O_5S$ requires 512.1398.

1-Cyclopropyl-6-fluoro-7-(4-((4-nitrophenyl)carbamothioyl)piperazin-1-yl)-4-oxo-1,4-dihydroquinoline-3-carboxylic acid (14). Thiourea (**14**) was obtained from CFX (250 mg, 0.755 mmol) and 4-nitrophenyl isothiocyanate (136 mg, 0.755 mmol), using general procedure A, as a white solid (235 mg, 61%). R_f = 0.51 (SiO_2; DCM:MeOH, 90:10);^{1}H NMR (400 MHz, DMSO-d_6) δ 15.21 (s, 1H), 9.99 (s, 1H), 8.67 (s, 1H), 8.19 (d, ^3J = 9.2 Hz, 2H), 7.94 (d, $^3J_{H-F}$ = 13.2 Hz, 1H), 7.64 (d, ^3J = 9.2 Hz, 2H), 7.58 (d, $^4J_{H-F}$ = 7.5 Hz, 1H), 4.17 (t, ^3J = 5.0 Hz, 4H), 3.83 (tt, ^3J = 4.0, ^3J =4.0 Hz,1H), 3.51 (t, ^3J = 5.0 Hz, 4H), 1.33 – 1.31 (m, 2H), 1.20 – 1.18 (m, 2H); ^{13}C NMR (101 MHz, DMSO-d_6) δ 180.9, 176.3, 165.9, 152.8 (d, $^1J_{C-F}$ = 249 Hz), 148.1, 147.7, 144.5 (d, $^2J_{C-F}$ = 10 Hz), 142.2, 139.2, 124.0, 122.6, 118.6 (d, $^3J_{C-F}$ = 8 Hz), 111.1

(d, $^2J_{C-F}$ = 22 Hz), 106.5, 106.3, 48.8, 48.0, 35.9, 7.6; LC-MS rt = 2.5 min; *m/z* 512 ([M+H]$^+$); HRMS found [M+H]$^+$ 512.1414, $C_{24}H_{23}FN_5O_5S$ requires 512.1398.

8-Ethyl-5-oxo-2-(4-(phenylcarbamoyl)piperazin-1-yl)-5,8-dihydropyrido[2,3-*d*]pyrimidine-6-carboxylic acid (15). Urea (**15**) was obtained from PA (250 mg, 0.824 mmol) and phenyisocyanate (90 µL, 0.824 mmol), using general procedure A, as a white solid (194 mg, 49%). R_f = 0.60 (SiO_2; DCM:MeOH, 90:10); ^{1}H NMR (400 MHz, DMSO-d_6) δ 14.80 (s, 1H), 9.26 (s, 1H), 8.99 (s, 1H), 8.64 (s, 1H), 7.47 (dd, ^3J = 8.6, ^4J = 4.0 Hz, 2H), 7.24 (dd, ^3J = 8.6, ^3J = 7.7 Hz, 2H), 6.95 (tt, ^3J = 7.7, ^4J = 4.0 Hz, 1H), 4.43 (q, ^3J = 7.1 Hz, 2H), 4.03 (bs, 2H), 3.96 (bs, 2H), 3.62 (bs, 4H), 1.38 (t, ^3J = 7.1 Hz, 3H); ^{13}C NMR (101 MHz, DMSO-d_6) δ 177.2, 165.3, 160.7, 160.3, 155.1, 154.9, 150.8, 140.4, 128.3, 121.9, 119.6, 109.6, 108.7, 45.9, 43.8, 43.5, 43.1, 14.4; LC-MS rt = 2.1 min; *m/z* 423 ([M+H]$^+$); HRMS found [M+H]$^+$ 423.1802, $C_{21}H_{23}N_6O_4$ requires 423.1775.

7-(4-(*tert*-Butoxycarbonyl)-3-methylpiperazin-1-yl)-1-cyclopropyl-6-fluoro-4-oxo-1,4-dihydroquinoline-3-carboxylic acid (Q2a). Boc-protected amine (**Q2a**) was obtained from 1-*N*-Boc-2-methylpiperazine (1.78 g, 8.88 mmol) and 7-chloro-1-cyclopropyl-6-fluoro-4-oxo-1,4-dihydroquinoline-3-carboxylic acid (**Q1a**) (500 mg, 1.78 mmol), using general procedure B, as an off-white solid (398 mg, 50%). R_f = 0.34 (SiO_2; EtOAc:Cyclohexane:AcOH, 90:8:2); ^{1}H NMR (500 MHz, DMSO-d_6) δ 15.20 (s, 1H), 8.66 (s, 1H), 7.92 (d, $^3J_{H-F}$ = 13.0 Hz, 1H), 7.56 (d, $^4J_{H-F}$ = 7.4 Hz, 1H), 4.28 (bs, 1H), 3.89 (d, ^2J = 13.2 Hz, 1H), 3.83 (tt, ^3J = 4.0, ^3J =4.0 Hz, 1H), 3.64 (d, ^2J = 12.0 Hz, 1H), 3.58 (d, ^2J = 12.5 Hz, 1H), 3.25 (d, ^2J = 13.2 Hz, 1H), 3.09 (app dd, ^2J = 12.5, ^4J = 3.6 Hz, 1H), 2.98 (app td, ^2J = 12.0, ^4J = 3.7 Hz, 1H), 1.43 (s, 9H), 1.36 – 1.29 (m, 2H), 1.26 (d, ^3J = 6.7 Hz, 3H), 1.19 – 1.17 (m, 2H); ^{13}C NMR (126 MHz, DMSO-d_6) δ 176.4, 165.9, 153.7, 152.9 (d, $^1J_{C-F}$ = 251 Hz), 148.0, 145.6, 139.2, 118.6, 111.0 (d, $^2J_{C-F}$ = 24 Hz), 106.8, 106.4, 79.1, 54.0, 48.9, 46.8, 38.3, 35.9, 28.1, 15.8, 7.6; LC-MS rt = 2.8 min; *m/z* 446 ([M+H]$^+$); HRMS found [M+H]$^+$ 446.2104, $C_{23}H_{29}FN_3O_5$ requires 446.2086.

7-(3-((*tert*-Butoxycarbonyl)amino)pyrrolidin-1-yl)-1-cyclopropyl-6-fluoro-4-oxo-1,4-dihydroquinoline-3-carboxylic acid (Q2b). Boc-protected amine (**Q2b**) was obtained from 3-(Boc-amino)pyrrolidine (1.65 g, 8.88 mmol) and 7-chloro-1-cyclopropyl-6-fluoro-4-oxo-1,4-dihydroquinoline-3-carboxylic acid (**Q1a**) (500 mg, 1.78 mmol), using general procedure B, as an off-white solid (462 mg, 60%) R_f = 0.27 (SiO_2; EtOAc:Cyclohexane:AcOH, 90:8:2); ^{1}H NMR (400 MHz, DMSO-d_6) δ 15.49 (s, 1H), 8.56 (s, 1H), 7.79 (d, $^3J_{H-F}$ = 14.3 Hz, 1H), 7.29 (d, ^3J = 6.6 Hz, 1H), 7.04 (d, $^4J_{H-F}$ = 7.6 Hz, 1H), 4.18 – 4.16 (m, 1H), 3.85 – 3.77 (m,

1H), 3.76 – 3.69 (m, 2H), 3.63 – 3.58 (m, 1H), 3.47 – 3.43 (m, 1H), 2.20 – 2.11 (m, 1H), 1.98 – 1.90 (m, 1H), 1.40 (s, 9H), 1.32 – 1.24 (m, 2H), 1.16 – 1.13 (m, 2H); ^{13}C NMR (101 MHz, DMSO-d_6) δ 175.8, 166.2, 155.2, 149.9 (d, $^1J_{C\text{-}F}$ = 246 Hz), 147.2, 141.5 (d, $^2J_{C\text{-}F}$ = 12 Hz), 139.7, 114.3 (d, $^3J_{C\text{-}F}$ = 7 Hz), 110.6 (d, $^2J_{C\text{-}F}$ = 22 Hz), 106.1, 100.3 (d, $^3J_{C\text{-}F}$ = 5 Hz), 78.0, 55.1, 49.8, 48.1, 35.6, 30.4, 28.2, 7.5; LC-MS rt = 2.4 min; *m/z* 432 ([M+H]$^+$); HRMS found [M+H]$^+$ 432.1951, $C_{22}H_{27}FN_3O_5$ requires 432.1929.

7-((2-((*tert*-Butoxycarbonyl)amino)ethyl)amino)-1-cyclopropyl-6-fluoro-4-oxo-1,4-dihydroquinoline-3-carboxylic acid (Q2c). Boc-protected amine (**Q2c**) was obtained from *N*-Boc-ethylenediamine (328 µL, 2.07 mmol) and 1-cyclopropyl-6,7-difluoro-4-oxo-1,4-dihydroquinoline-3-carboxylic acid (**Q1b**) (500 mg, 1.89 mmol), using general procedure C, as a beige solid (445 mg, 58%). R_f = 0.51 (SiO_2; DCM:MeOH:NEt_3, 90:8:2); ^{1}H NMR (400 MHz, DMSO-d_6) δ 15.64 (s, 1H), 8.57 (s, 1H), 7.79 (d, $^3J_{H\text{-}F}$ = 11.8 Hz, 1H), 7.30 (d, $^4J_{H\text{-}F}$ = 7.4 Hz, 1H), 7.13 – 7.08 (m, 2H), 3.75 (tt, ^3J = 4.0, ^3J = 4.0 Hz, 1H), 3.18 (app q, ^3J = 6.6 Hz, 2H), 1.37 (s, 9H), 1.34 (bs, 2H), 1.16 (bs, 2H)[II]; ^{13}C NMR (101 MHz, DMSO-d_6) δ 176.0, 166.3, 156.0, 151.1 (d, $^1J_{C\text{-}F}$ = 247 Hz), 147.0, 142.6 (d, $^2J_{C\text{-}F}$ = 14 Hz), 140.6, 113.9 (d, $^3J_{C\text{-}F}$ = 7 Hz), 108.9 (d, $^2J_{C\text{-}F}$ = 20 Hz), 106.2, 96.5 (d, $^3J_{C\text{-}F}$ = 4 Hz), 78.0, 42.4, 38.1, 36.0, 28.2, 7.6; LC-MS rt = 2.2 min; *m/z* 406 ([M+H]$^+$); HRMS found [M+H]$^+$ 406.1770, $C_{20}H_{25}FN_3O_5$ requires 406.1773.

1-Cyclopropyl-6-fluoro-7-(3-methyl-4-((4-(trifluoromethyl)phenyl)carbamothioyl)piperazin-1-yl)-4-oxo-1,4-dihydroquinoline-3-carboxylic acid (16). 4-(3-Carboxy-1-cyclopropyl-6-fluoro-4-oxo-1,4-dihydroquinolin-7-yl)-2-methylpiperazin-1-ium chloride (**Q3a**) was obtained from **Q2a** (300 mg, 0.67 mmol), using general procedure D, as a light yellow sold (257 mg, 100%). R_f = 0.13 (SiO_2; DCM:MeOH:NEt_3, 90:8:2). **Q3a** (150 mg, 0.359 mmol) was used without purification for the subsequent isothiocyanate coupling with 4-(trifluoromethyl)phenyl isothiocyanate (80 mg, 0.394 mmol) using general procedure A, to afford thiourea (**16**) a light yellow solid (88 mg, 45%). R_f = 0.63 (SiO_2; DCM:MeOH, 9:1); ^{1}H NMR (400 MHz, DMSO-d_6) δ 15.21 (s, 1H), 9.64 (s, 1H), 8.67 (s, 1H), 7.95 (d, $^3J_{H\text{-}F}$ = 13.1 Hz, 1H), 7.66 (d, ^3J = 8.5 Hz, 2H), 7.57 (d, ^3J = 8.5 Hz, 3H), 5.28 (s, 1H), 4.64 (bs, 1H), 3.83 (s, 1H), 3.78 (d, ^2J = 11.7 Hz, 1H), 3.71 (d, ^2J =

[II]2H of ethylene protons obscured by water peak.

12.3 Hz, 1H), 3.62 (app t, ^{2}J = 12.5 Hz, 1H), 3.29 (d, ^{2}J = 12.3 Hz, 1H), 3.17 (app t, ^{2}J = 11.7 Hz, 1H), 1.41 (d, ^{3}J = 4.0 Hz, 3H), 1.33 (d, ^{3}J = 4.0 Hz, 2H), 1.21 (d, ^{3}J = 4.0 Hz, 2H); ^{13}C NMR (101 MHz, DMSO-d_6) δ 181.5, 176.4, 165.9, 152.8 (d, $^{1}J_{C\text{-}F}$ = 249 Hz), 148.1 (d, $^{2}J_{C\text{-}F}$ = 9 Hz), 145.2, 144.9, 139.2, 125.1, 124.6 (q, $^{3}J_{C\text{-}F}$ = 4 Hz), 124.4 (q, $^{1}J_{C\text{-}F}$ = 272 Hz), 123.9 (q, $^{2}J_{C\text{-}F}$ = 32 Hz), 118.6, 111.0, 106.7, 106.3, 53.6, 51.5, 48.6, 42.9, 35.9, 15.3, 7.6; LC-MS rt = 2.8 min; *m/z* 549 ($[M+H]^+$); HRMS found $[M+H]^+$ 549.1581, $C_{26}H_{25}F_4N_4O_3S$ requires 549.1578.

1-Cyclopropyl-6-fluoro-4-oxo-7-(3-(3-(4-(trifluoromethyl)phenyl)thioureido)pyrrolidin-1-yl)-1,4-dihydroquinoline-3-carboxylic acid (17). 1-(3-Carboxy-1-cyclopropyl-6-fluoro-4-oxo-1,4-dihydroquinolin-7-yl)pyrrolidin-3-aminium chloride (**Q3b**) was obtained from **Q2b** (200 mg, 0.46 mmol), using general procedure D as a bright yellow solid (170 mg, 100%). R_f = 0.06 (SiO_2; DCM:MeOH:Et_3N, 90:8:2). **Q3b** (115 mg, 0.313 mmol) was used without purification for the subsequent isothiocyanate coupling with 4-(trifluoromethyl)phenyl isothiocyanate (64 mg, 0.313 mmol) using general procedure A, to afford thiourea (**17**) a light yellow solid (70 mg, 42%). R_f = 0.37 (SiO_2; DCM:MeOH:NEt_3, 90:8:2); ^{1}H NMR (400 MHz, DMSO-d_6) δ 15.50 (s, 1H), 9.93 (s, 1H), 8.58 (s, 1H), 8.56 (bs, 1H), 7.83 (d, $^{3}J_{H\text{-}F}$ = 14.2 Hz, 1H), 7.77 (d, ^{3}J = 8.3 Hz, 2H), 7.65 (d, ^{3}J = 8.3 Hz, 2H), 7.10 (d, $^{4}J_{H\text{-}F}$ = 7.5 Hz, 1H), 4.94 (bs, 1H), 3.98 (bs, 1H), 3.76 (bs, 3H), 3.64 (d, ^{2}J = 10.7 Hz, 1H), 2.38 – 2.30 (m, 1H), 2.16 – 2.12 (m, 1H), 1.30 (bs, 2H), 1.14 (bs, 2H); ^{13}C NMR (101 MHz, DMSO-d_6) δ 180.3, 175.9, 166.2, 150.0 (d, $^{1}J_{C\text{-}F}$ = 247 Hz), 147.5, 143.4, 141.5 (d, $^{2}J_{C\text{-}F}$ = 6 Hz), 139.8, 125.6, 124.4 (q, $^{1}J_{C\text{-}F}$ = 272 Hz), 123.4 (q, $^{2}J_{C\text{-}F}$ = 32 Hz), 121.6, 114.7, 110.8, 106.2, 100.7, 54.9, 53.3, 48.2, 35.7, 30.1, 7.5; LC-MS rt = 2.7 min; *m/z* 535 ($[M+H]^+$); HRMS found $[M+H]^+$ 535.1433, $C_{25}H_{23}F_4N_4O_3S$ requires 535.1422.

1-Cyclopropyl-6-fluoro-4-oxo-7-((2-(3-(4-(trifluoromethyl)phenyl)thioureido)ethyl)amino)-1,4-dihydroquinoline-3-carboxylic acid (18). 2-((3-Carboxy-1-cyclopropyl-6-fluoro-4-oxo-1,4-dihydroquinolin-7-yl)amino)ethan-1-aminium chloride (**Q3a**) was obtained from **Q2a** (300 mg, 0.74 mmol), using general procedure D, as a light brown solid (253 mg, 100%). R_f = 0.07 (SiO_2; DCM:MeOH, 50:50). **Q2a** (150 mg, 0.44 mmol) was used without purification for the subsequent isothiocyanate coupling with 4-(trifluoromethyl)phenyl isothiocyanate (89 mg, 0.44 mmol) to form thiourea (**18**), using general procedure A (carried out at 60 °C), as a beige

solid (140 mg, 64%). R_f = 0.39 (SiO_2; DCM:MeOH, 90:10); ^{1}H NMR (500 MHz, DMSO-d_6) δ 15.62 (s, 1H), 10.01 (s, 1H), 8.55 (s, 1H), 8.27 (bs, 1H), 7.81 (d, $^3J_{H-F}$ = 11.8 Hz, 1H), 7.64 (s, 4H), 7.33 (d, $^4J_{H-F}$ = 7.3 Hz, 1H), 7.24 (bs, 1H), 3.82 (app q, ^3J = 6.3 Hz, 2H), 3.69 (tt, ^3J = 4.0, ^3J =4.0 Hz, 1H), 3.58 (app q, ^3J = 6.3 Hz, 2H), 1.35 – 1.27 (m, 2H), 1.17 – 1.12 (m, 2H); ^{13}C NMR (126 MHz, DMSO-d_6) δ 180.7, 175.9, 166.3, 150.0 (d, $^1J_{C-F}$ = 246 Hz), 147.0, 142.9 (d, $^2J_{C-F}$ = 14 Hz) 142.7, 140.6, 125.8, 124.3 (q, $^1J_{C-F}$ = 271 Hz), 123.7 (q, $^2J_{C-F}$ = 33 Hz), 122.2, 113.9 (d, $^3J_{C-F}$ = 6 Hz), 108.8 (d, $^2J_{C-F}$ = 19 Hz), 106.2, 96.5 (d, $^3J_{C-F}$ = 4 Hz), 42.4, 41.2, 36.0, 7.8; LC-MS rt = 2.5 min; *m/z* 509 ([M+H]$^+$); HRMS found [M+H]$^+$ 509.1275, $C_{23}H_{21}F_4N_4O_3S$ requires 509.1265.

***tert*-Butyl(2-(1-cyclopropyl-6-fluoro-4-oxo-7-(4-((4-(trifluoromethyl)phenyl)carbamothioyl)piperazin-1-yl)-1,4-dihydroquinoline-3-carboxamido)ethyl)carbamate (19).** To **10** (50 mg, 0.094 mmol) in DMF (2 mL) was added *N,N*-diisopropylethylamine (DIPEA) (49 µL, 0.28 mmol), followed by (benzotriazol-1-yloxy)tripyrrolidinophosphonium hexa- fluorophosphate (PyBOP) (49 mg, 0.094 mmol). The mixture was stirred at RT for 10 min before addition of *N*-Boc-ethylenediamine (16 µL, 0.10 mmol). The resulting mixture was then stirred at RT overnight. Afterward, EtOAc (30 mL) was added, and the organic layer was washed with 5% w/v LiCl(aq.), water, and brine, then dried over $MgSO_4$. The solvent was removed by vacuum and the residue was purified by column chromatography (EtOAc:Acetone:MeOH, 7:2:1) to provide the product as a white solid (51 mg, 81%). R_f = 0.63 (SiO_2; EtOAc:Acetone:MeOH, 7:2:1); ^{1}H NMR (400 MHz, DMSO-d_6) δ 9.94 (t, ^3J = 6.0 Hz, 1H), 9.75 (s, 1H), 8.63 (s, 1H), 7.88 (d, $^3J_{H-F}$ = 13.3 Hz, 1H), 7.66 (d, ^3J = 8.6 Hz, 2H), 7.58 (d, ^3J = 8.6 Hz, 2H), 7.53 (d, $^4J_{H-F}$ = 7.5 Hz, 1H), 6.95 (t, ^3J = 5.9 Hz, 1H), 4.16 (t, ^3J = 6.0 Hz, 4H), 3.74 (tt, ^3J = 4.0, 4.0 Hz, 1H), 3.42 (t, ^3J = 6.0 Hz, 4H), 3.36 (app q, ^3J = 6.0, ^3J = 6.0 Hz, 2H), 3.07 (app q, ^3J = 6.0, ^3J = 6.0 Hz, 2H), 1.37 (s, 9H), 1.34 – 1.28 (m, 2H), 1.15 – 1.07 (m, 2H); ^{13}C NMR (101 MHz, DMSO-d_6) δ 181.3, 174.2, 164.2, 155.7, 152.5 (d, $^1J_{C-F}$ = 247 Hz), 146.8, 144.8, 143.6 (d, $^2J_{C-F}$ = 11 Hz), 138.4, 125.2 (q, $^3J_{C-F}$ = 4 Hz), 124,4 (q, $^1J_{C-F}$ = 273 Hz), 124.2, 123.8 (d, $^2J_{C-F}$ = 35 Hz), 120.9, 111.2 (d, $^2J_{C-F}$ = 35 Hz), 110.2, 106.2, 77.6, 49.1, 47.9, 38.3, 35.0, 28.2, 7.6[III]; LC-MS rt = 2.8 min; *m/z* 677 ([M+H]$^+$); HRMS found [M+H]$^+$ 677.2540, $C_{32}H_{37}F_4N_6O_4S$ requires 677.2528.

[III]C35 obscured by residual DMSO peak in the C^{13} NMR spectra

***N*-(2a-Aminoethyl)-1-cyclopropyl-6-fluoro-4-oxo-7-(4-((4-(trifluoromethyl)phenyl)carbamothioyl)piperazin-1-yl)-1,4-dihydroquinoline-3-carboxamide (20).** 2-(1-Cyclopropyl-6-fluoro-4-oxo-7-(4-((4-(trifluoromethyl)phenyl)carbamothioyl)piperazin-1-yl)-1,4-dihydroquinoline-3-carboxamido)ethan-1-aminium chloride was obtained from **19**, using general procedure D. The crude product was purified using SCX resin, and the title compound (**20**) was eluted with ammonia (3.5 M) in 1:1 v/v MeOH:DCM and isolated as a white solid (29 mg, 76%). R_f = 0.37 (SiO_2; EtOAc:Acetone:MeOH (3.5 M NH_3), 7:2:1); ^{1}H NMR (400 MHz, DMSO-d_6) δ 9.96 (t, ^3J = 5.9 Hz, 1H), 8.63 (s, 1H), 7.89 (d, $^3J_{H-F}$ = 13.4 Hz, 1H), 7.64 (d, ^3J = 8.6 Hz, 2H), 7.56 (d, ^3J = 8.6 Hz, 2H), 7.52 (d, $^4J_{H-F}$ = 7.4 Hz, 1H), 4.16 (t, ^3J = 5.1 Hz, 4H), 3.73 (tt, ^3J = 3.7, 3.7 Hz, 1H), 3.42 (t, ^3J = 5.1 Hz, 4H), 3.32 (app q, ^3J = 6.1, ^3J = 5.9 Hz, 2H), 2.70 (t, ^3J = 6.1 Hz, 2H), 2.42 (bs, 2H), 1.33 – 1.28 (m, 2H), 1.13 – 1.09 (m, 2H)[IV]; ^{13}C NMR (101 MHz, DMSO-d_6) δ 181.0, 174.2, 164.0, 152.5 (d, $^1J_{C-F}$ = 248 Hz), 146.7, 145.5, 143.7 (d, $^2J_{C-F}$ = 18 Hz), 138.4, 125.1 (app d, $^3J_{C-F}$ = 4 Hz), 124.4 (app d, $^1J_{C-F}$ = 273 Hz), 124.2, 123.5 (app d, $^2J_{C-F}$ = 30 Hz), 120.9 (d, $^3J_{C-F}$ = 7 Hz), 111.2 (d, $^2J_{C-F}$ = 23 Hz), 110.3, 106.2, 49.1, 47.8, 41.6, 41.4, 35.0, 7.6. LC-MS rt = 2.0 min; *m/z* 577 ($[M+H]^+$); HRMS found $[M+H]^+$ 577.2002, $C_{27}H_{29}F_4N_6O_2S$ requires 577.2003.

1-Cyclopropyl-6-fluoro-4-oxo-7-(4-(phenylcarbamoyl)piperazin-1-yl)-1,4-dihydroquinoline-3-carboxylic acid (21). The urea (**21**) was obtained from CFX (371 mg, 1.12 mmol) and phenyl isocyanate (113 mg, 1.12 mmol), using general procedure A, as a white solid (195 mg, 39%), R_f = 0.47 (SiO_2; DCM:MeOH, 97.5:2.5); ^{1}H NMR (400 MHz, DMSO-d_6) δ 15.17 (s, 1H), 8.66 (m, 2H), 7.91 (d, $^3J_{H-F}$ = 13.2 Hz, 1H), 7.60 (d, $^4J_{H-F}$ = 7.5 Hz, 1H), 7.48 (dd, ^3J = 8.6, 1.1 Hz, 2H), 7.29-7.20 (m, 2H), 6.99 – 6.91 (m, 1H), 3.83 (tt, ^3J = 7.2, 4.0 Hz, 1H), 3.70 (t, ^3J = 5.0 Hz, 4H), 3.37 (t, ^3J = 5.0 Hz, 4H), 1.37 – 1.29 (m, 2H), 1.24 – 1.17 (m, 2H). ^{13}C NMR (101 MHz, DMSO-d_6) δ 176.8, 166.4, 155.4, 148.5, 140.9, 139.6, 128.80, 128.80, 122.3, 120.1, 111.6, 107.3, 107.1, 49.85, 49.85, 49.8, 43.99, 43.99, 36.36, 8.07; LC-MS rt = 2.1 min; *m/z* 451 ($[M+H]^+$); HRMS found $[M+H]^+$ 451.1801, $C_{25}H_{24}N_4O_4F$ requires 451.1782.

[IV] Thioamide proton not observed in the proton NMR spectra

***tert*-Butyl(2-(1-cyclopropyl-6-fluoro-4-oxo-7-(4-(phenylcarbamoyl)piperazin-1-yl)-1,4-dihydroquinoline-3-carboxamido)ethyl)carbamate (22)**. To **21** (100 mg, 0.22 mmol) in DMF (15 mL) was added DIPEA (116 µL, 0.66 mmol), PyBOP (114 mg, 0.22 mmol) and the mixture stirred at RT for 15 min. *tert*-Butyl (2-aminoethyl)carbamate (39 mg, 0.25 mmol) was added and the resultant mixture stirred overnight at RT. Afterward, EtOAc (30 mL) was added, and the organic layer was washed with 5% w/v LiCl(aq.), water, and brine, then dried over $MgSO_4$. The solvent was removed by vacuum and the residue was purified by column chromatography (EtOAc:Acetone:MeOH, 7:2:1) to provide the product as a white solid (44 mg, 33%), R_f = 0.84 (SiO^2; EtOAc:Acetone:MeOH, 70:20:10); ^{1}H NMR (400 MHz, DMSO-d_6) δ 9.93 (t, ^3J = 5.9 Hz, 1H), 8.66 (s, 1H), 8.62 (s, 1H), 7.86 (d, $^3J_{H-F}$ = 13.3 Hz, 1H), 7.53 (d, $^2J_{H-F}$ = 7.4 Hz, 1H), 7.48 (d, ^3J = 7.8 Hz, 2H), 7.24 (t, ^3J = 7.9 Hz, 2H), 6.94 (app q, ^3J = 6.4, 5.4 Hz, 2H), 3.74 (tt, ^3J = 7.0, 3.2 Hz, 1H), 3.69 (t, 4H), 3.37 (app q, 2H), 3.31 (t, 3H), 3.08 (app q, 2H), 1.37 (s, 9H), 1.33 – 1.27 (m, 2H), 1.14 – 1.07 (m, 2H); ^{13}C NMR (101 MHz, DMSO-d_6) δ 174.2, 164.2, 155.7, 154.9, 146.7, 144.1, 140.4, 138.4, 128.3, 121.8, 121.0, 119.6, 111.1, 110.1, 106.4, 77.6, 49.5, 46.6, 43.6, 38.3, 35.0, 28.2, 7.6[V]; LC-MS rt = 2.7 min; *m/z* 591 ($[M-H]^-$); HRMS found $[M-H]^-$ 591.2728, $C_{31}H_{36}N_6O_5F$ requires 591.2731

***N*-(2-Aminoethyl)-1-cyclopropyl-6-fluoro-4-oxo-7-(4-(phenylcarbamoyl)piperazin-1-yl)-1,4-dihydroquinoline-3-carboxamide (23).** The amine (**23**) was obtained from deprotection of **22** (34 mg, 0.057 mmol) with 4 M HCl in dioxane using general procedure D, as a white solid (19 mg, 68%), R_f = 0.38 (SiO_2, EtOAc:Acetone:MeOH (3.5 M NH_3)), 70:20:10); ^{1}H NMR (400 MHz, DMSO-d_6) δ 9.95 (t, ^3J = 5.7 Hz, 1H), 8.66 (s, 1H), 8.62 (s, 1H), 7.85 (d, $^3J_{H-F}$ = 13.3 Hz, 1H), 7.52 (d, $^2J_{H-F}$ = 7.5 Hz, 1H), 7.48 (d, ^3J = 7.6 Hz, 2H), 7.24 (t, ^3J = 7.9 Hz, 2H), 6.95 (t, ^3J = 7.3 Hz, 1H), 3.73 (m, 1H), 3.71-3.64 (m, 4H), 3.32-329 (m, 6H), 2.69 (app t, ^3J = 6.3 Hz, 2H), 1.34-1.26 (m, 2H), 1.14 – 1.07 (m, 2H); ^{13}C NMR (101 MHz, DMSO-d_6) δ 174.2, 164.0, 154.9, 152.6 (d, $^1J_{C-F}$ = 247 Hz), 146.7, 144.1 (d, $^2J_{C-F}$ = 10 Hz), 140.4, 138.3, 128.3, 121.8, 121.0 ($^3J_{C-F}$ = 7 Hz), 119.6, 111.3 (d, $^2J_{C-F}$ = 23 Hz), 110.3, 106.4, 49.5, 43.6, 41.8, 41.5, 35.0, 7.6; LC-MS rt = 1.9 min; *m/z* 493 ($[M+H]^+$); HRMS found $[M+H]^+$ 493.2371, $C_{26}H_{30}N_6O_3F$ requires 493.2363

[V] –**C**F not observed in the C^{13} NMR spectra

2.5. NMR spectra of final compounds

2.5.1. 8-Ethyl-5-oxo-2-(4-((3-(trifluoromethyl)phenyl)carbamothioyl)piperazin-1-yl)-5,8-dihydropyrido[2,3-*d*]pyrimidine-6-carboxylic acid (1).

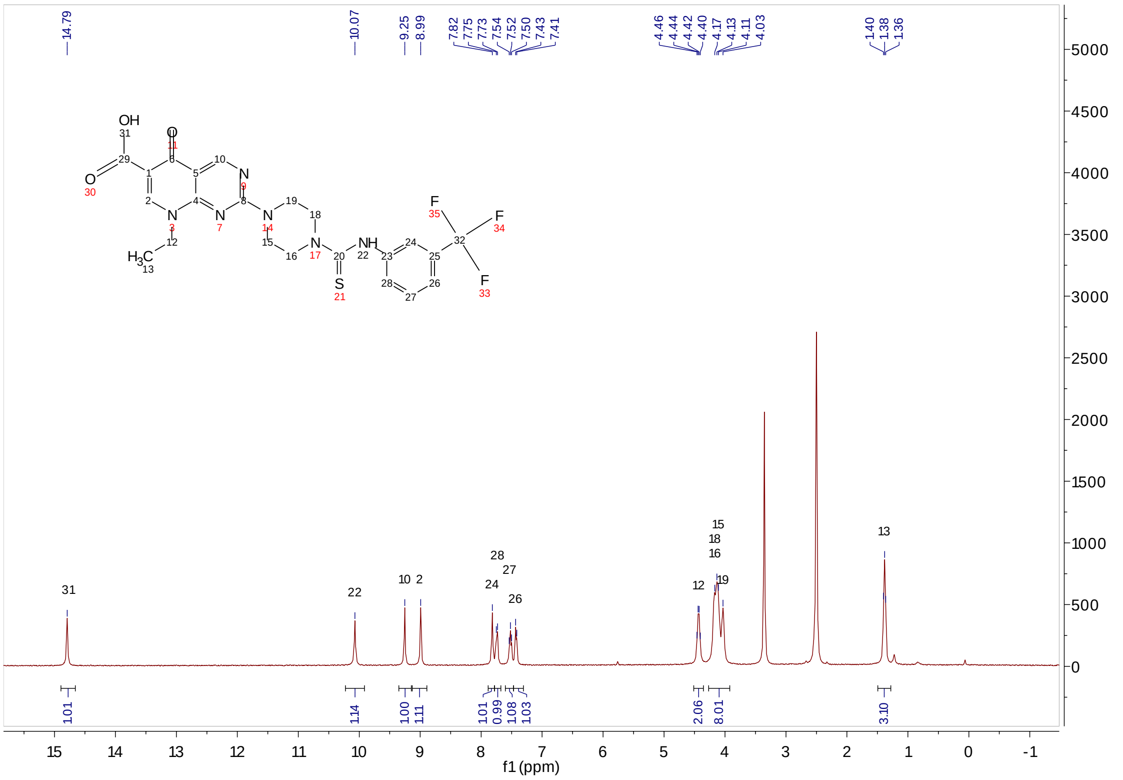

Figure S4. ^{1}H NMR (400 MHz, DMSO-d6) at 298 K of 1.

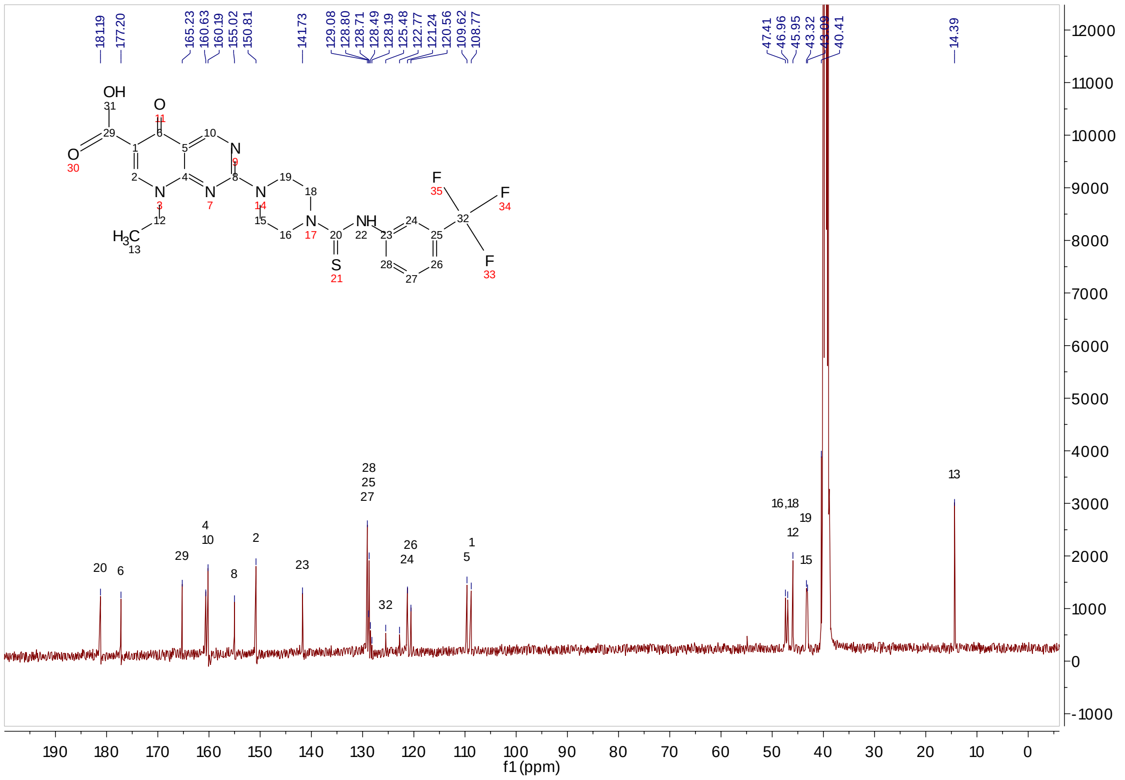

Figure S5. ^{13}C NMR (400 MHz, DMSO-d_6) at 298 K of 1.

2.5.2. 8-Ethyl-2-(4-((3-fluorophenyl)carbamothioyl)piperazin-1-yl)-5-oxo-5,8-dihydropyrido[2,3-*d*]pyrimidine-6-carboxylic acid (2).

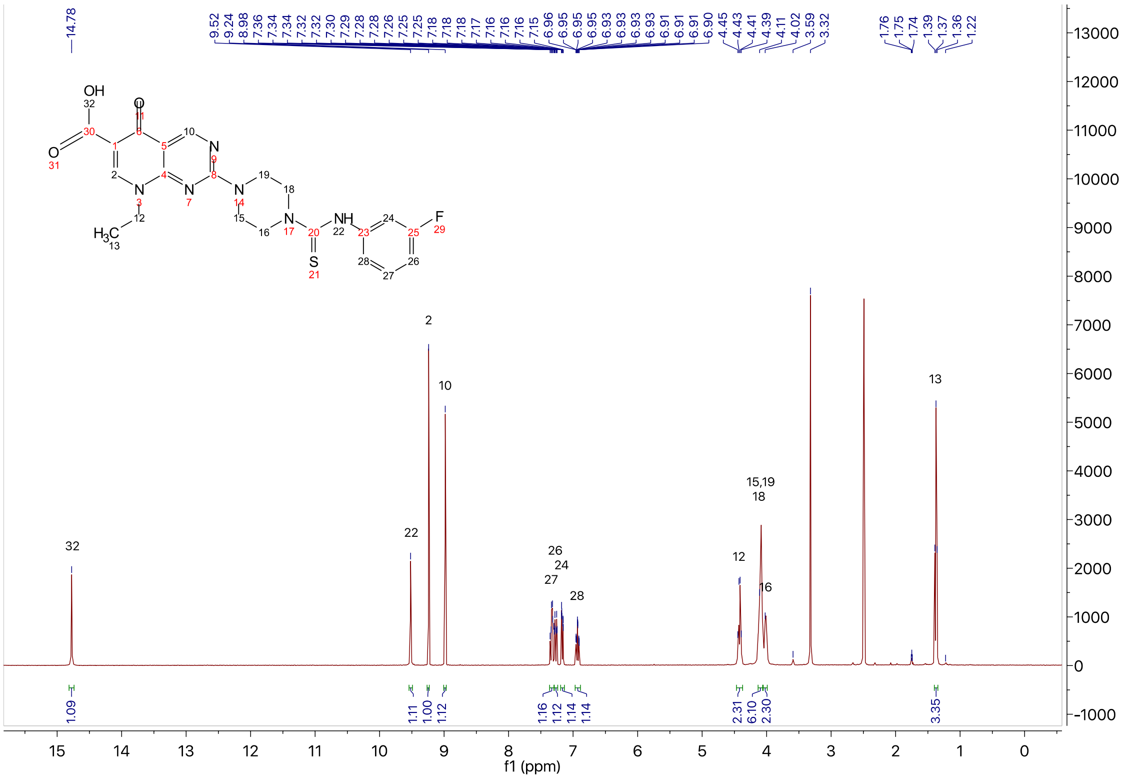

Figure S6. ^{1}H NMR (400 MHz, DMSO-d_6) at 298 K of 2.

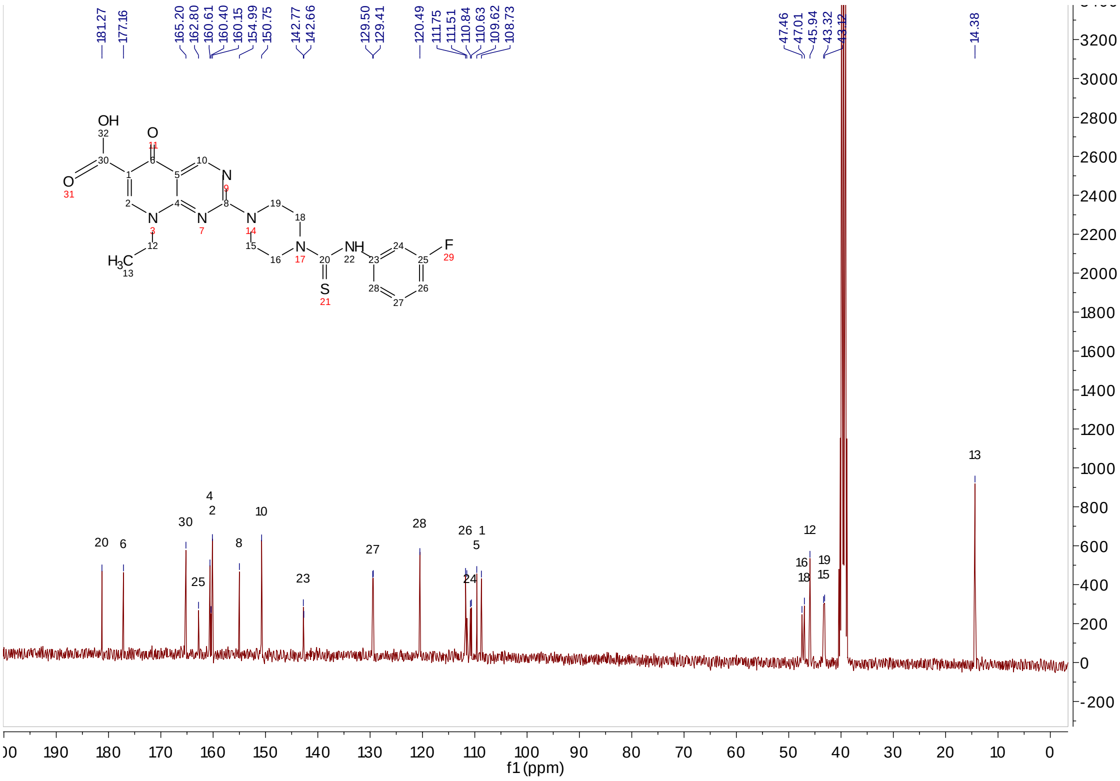

Figure S7. ^{13}C NMR (400 MHz, DMSO-d_6) at 298 K of 2.

2.5.3. 2-(4-((3,5-Bis(trifluoromethyl)phenyl)carbamothioyl)piperazin-1-yl)-8-ethyl-5-oxo-5,8-dihydropyrido[2,3-*d*]pyrimidine-6-carboxylic acid (3).

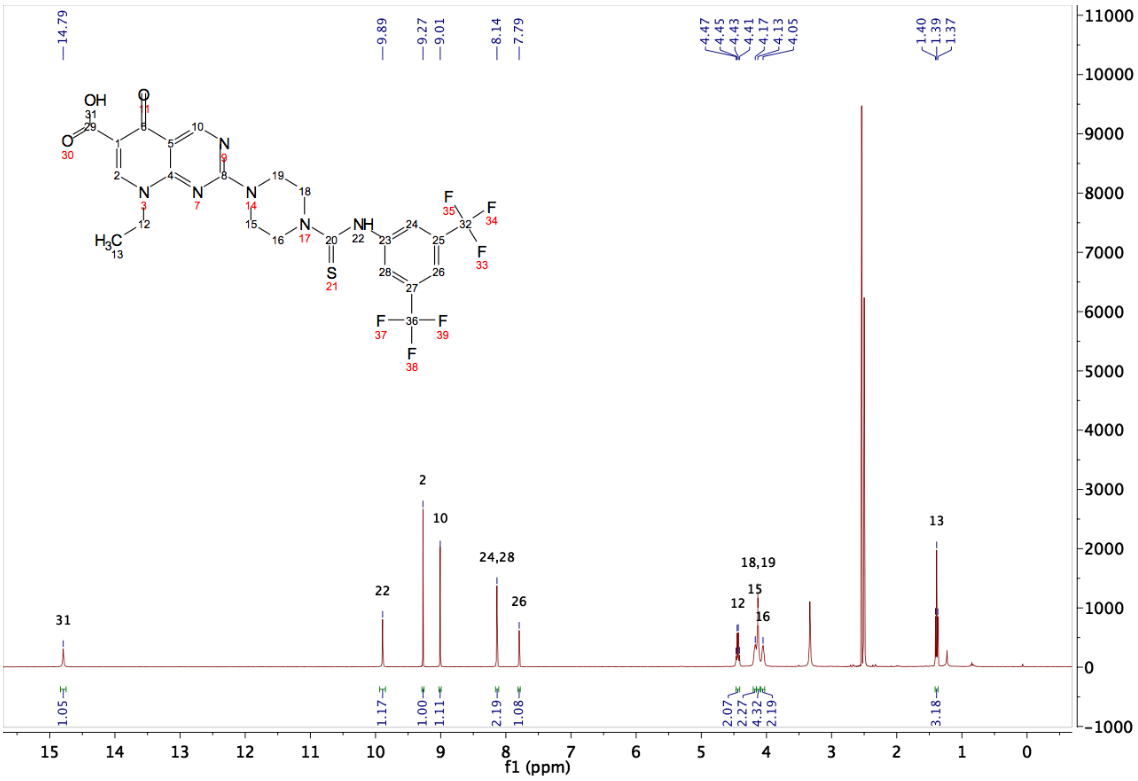

Figure S8. **^{1}H NMR (400 MHz, DMSO-*d*$_6$) at 298 K of 3.**

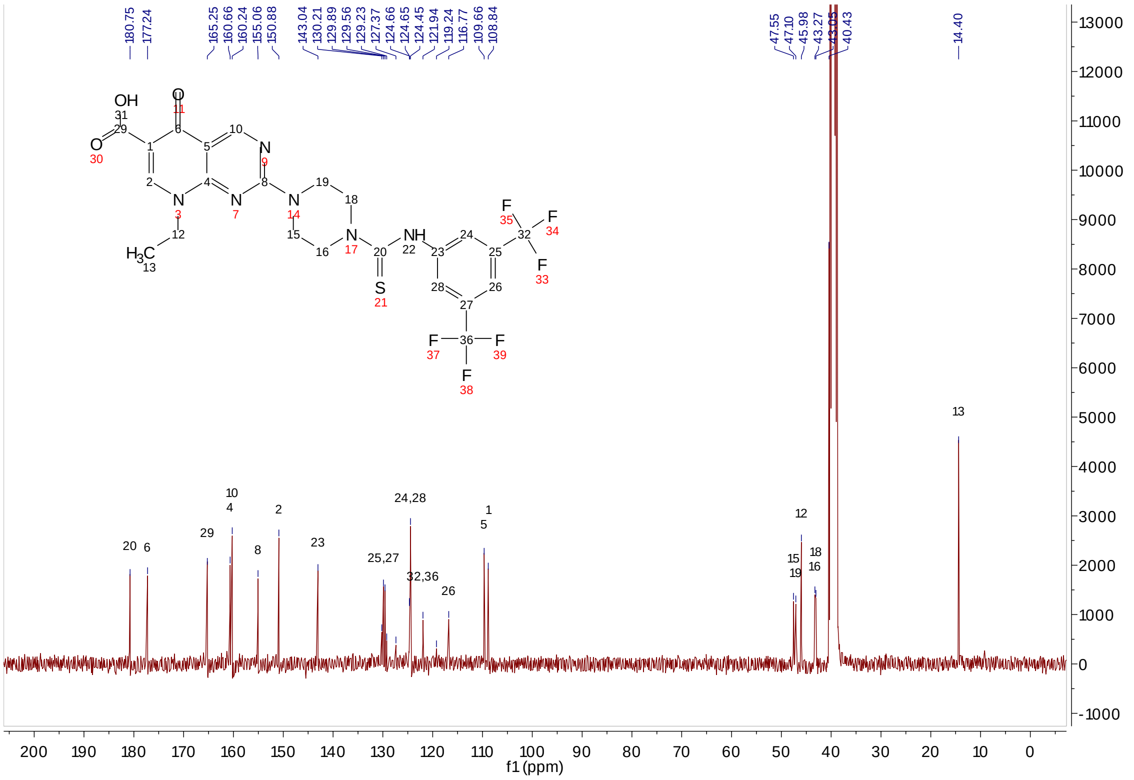

Figure S9. **^{13}C NMR (400 MHz, DMSO-*d*$_6$) at 298 K of 3.**

2.5.4. 8-Ethyl-5-oxo-2-(4-((4-(trifluoromethyl)phenyl)carbamothioyl)piperazin-1-yl)-5,8-dihydropyrido[2,3-*d*]pyrimidine-6-carboxylic acid (4).

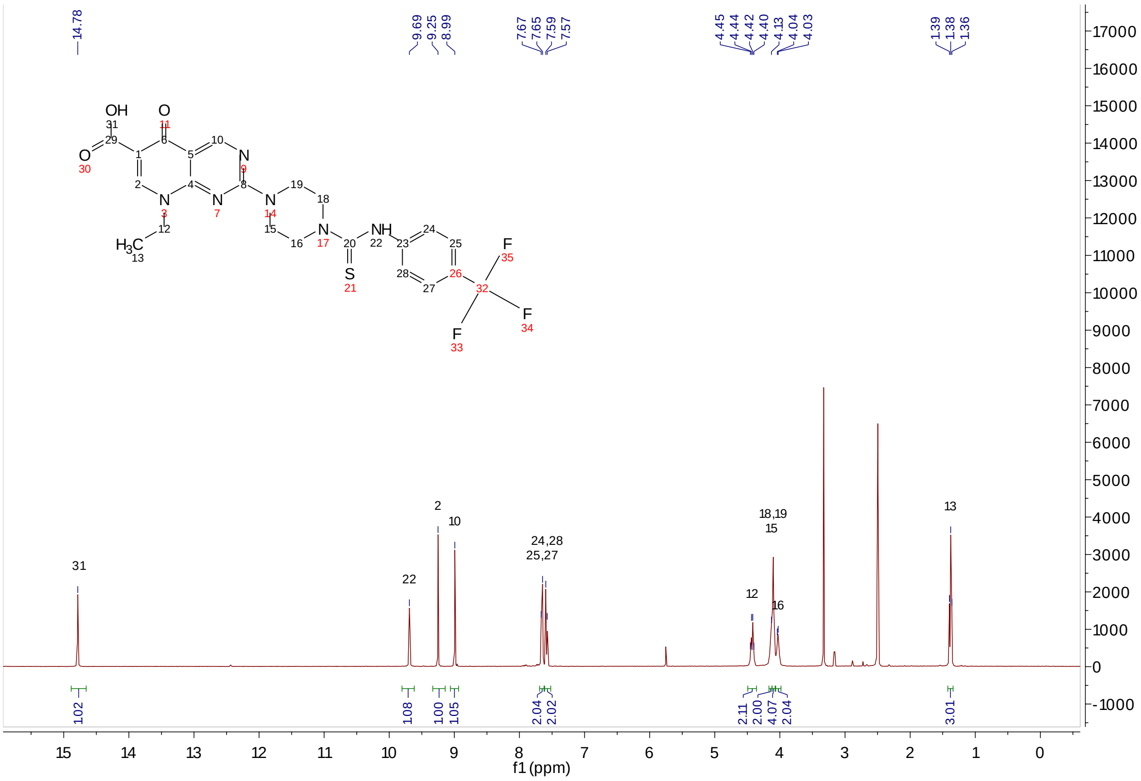

Figure S10. ^{1}**H NMR (400 MHz, DMSO-*d*$_6$) at 298 K of 4.**

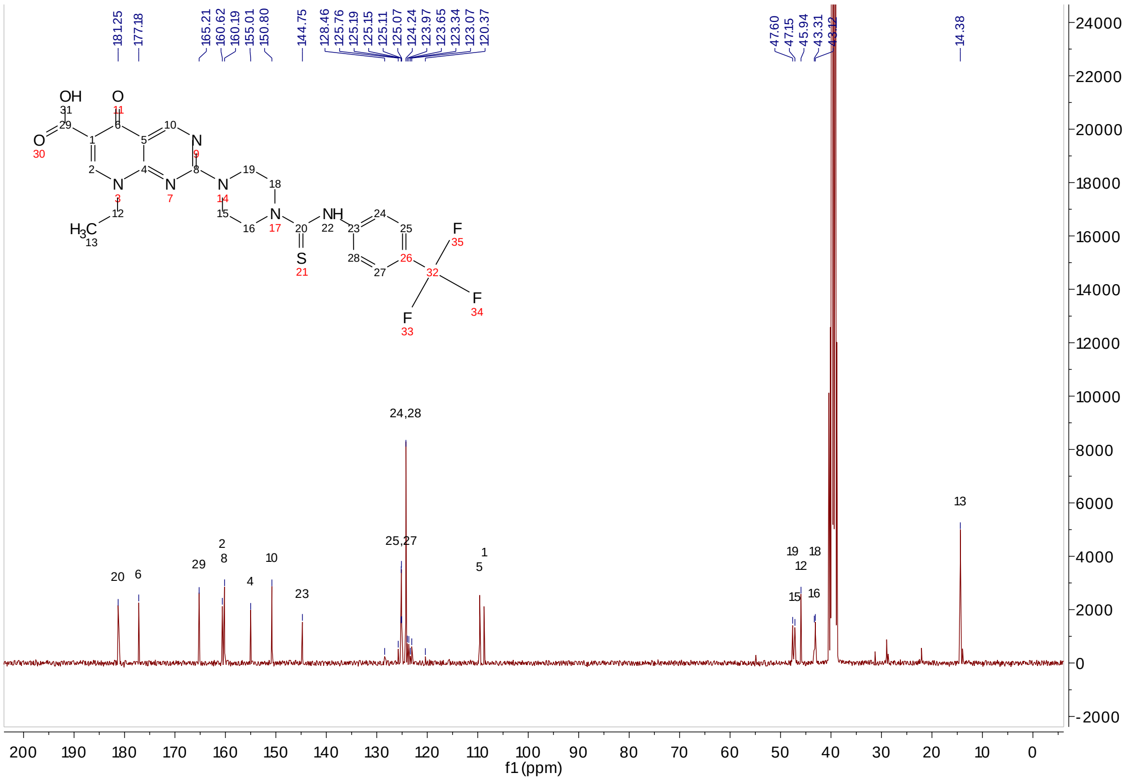

Figure S11. ^{13}**C NMR (400 MHz, DMSO-*d*$_6$) at 298 K of 4.**

2.5.5. 2-(4-((3-Chlorophenyl)carbamothioyl)piperazin-1-yl)-8-ethyl-5-oxo-5,8-dihydropyrido[2,3-*d*]pyrimidine-6-carboxylic acid (5).

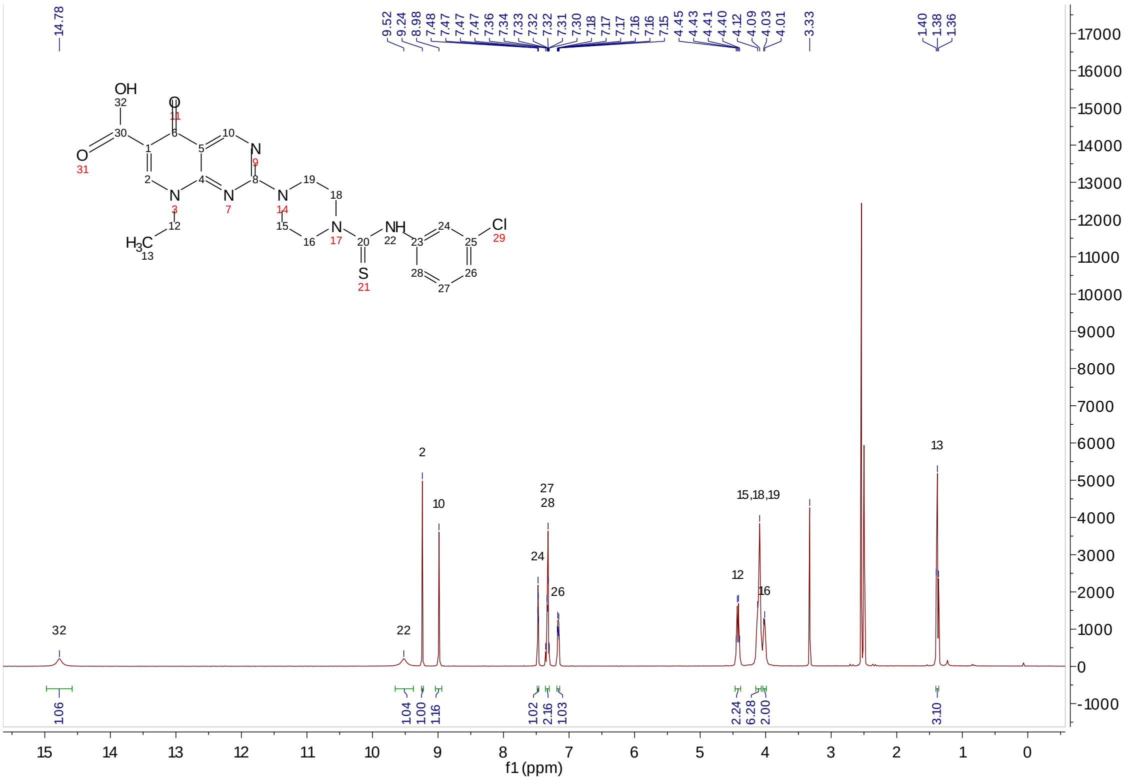

Figure S12. ^{1}H **NMR (400 MHz, DMSO-*d*$_6$) at 298 K of 5.**

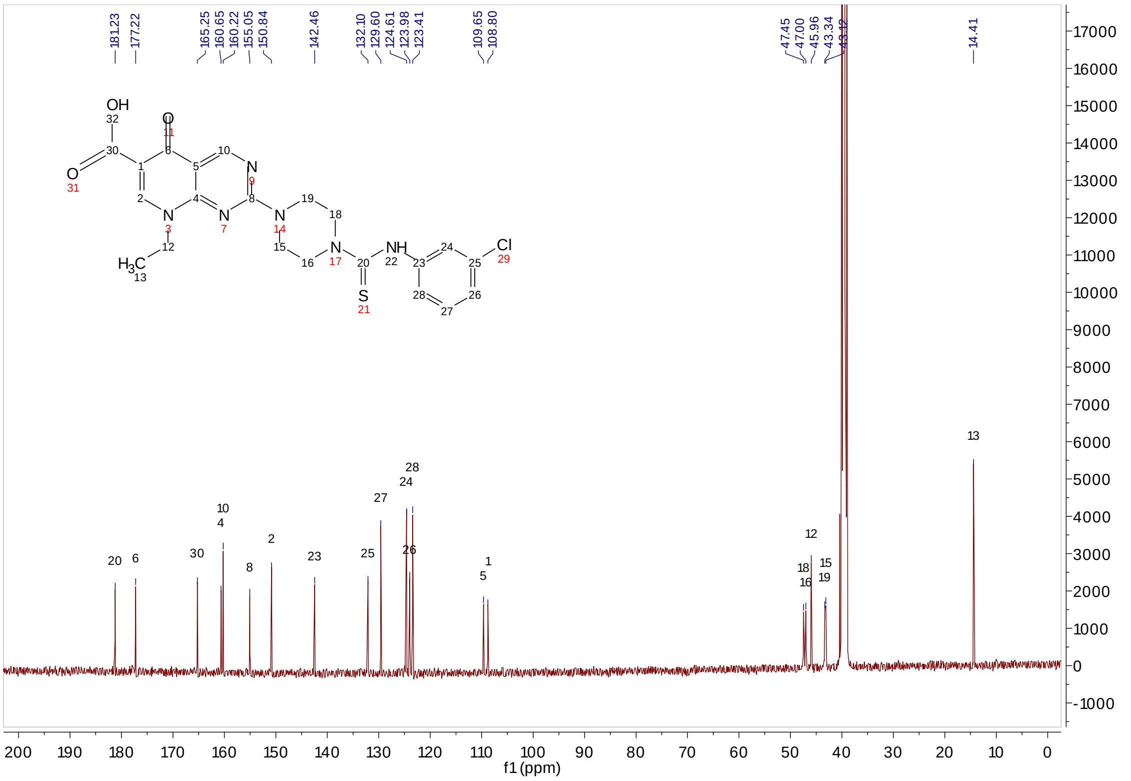

Figure S13. ^{13}C **NMR (500 MHz, DMSO-*d*$_6$) at 298 K of 5.**

2.5.6. 2-(4-((3,5-Dichlorophenyl)carbamothioyl)piperazin-1-yl)-8-ethyl-5-oxo-5,8-dihydropyrido[2,3-*d*]pyrimidine-6-carboxylic acid (6).

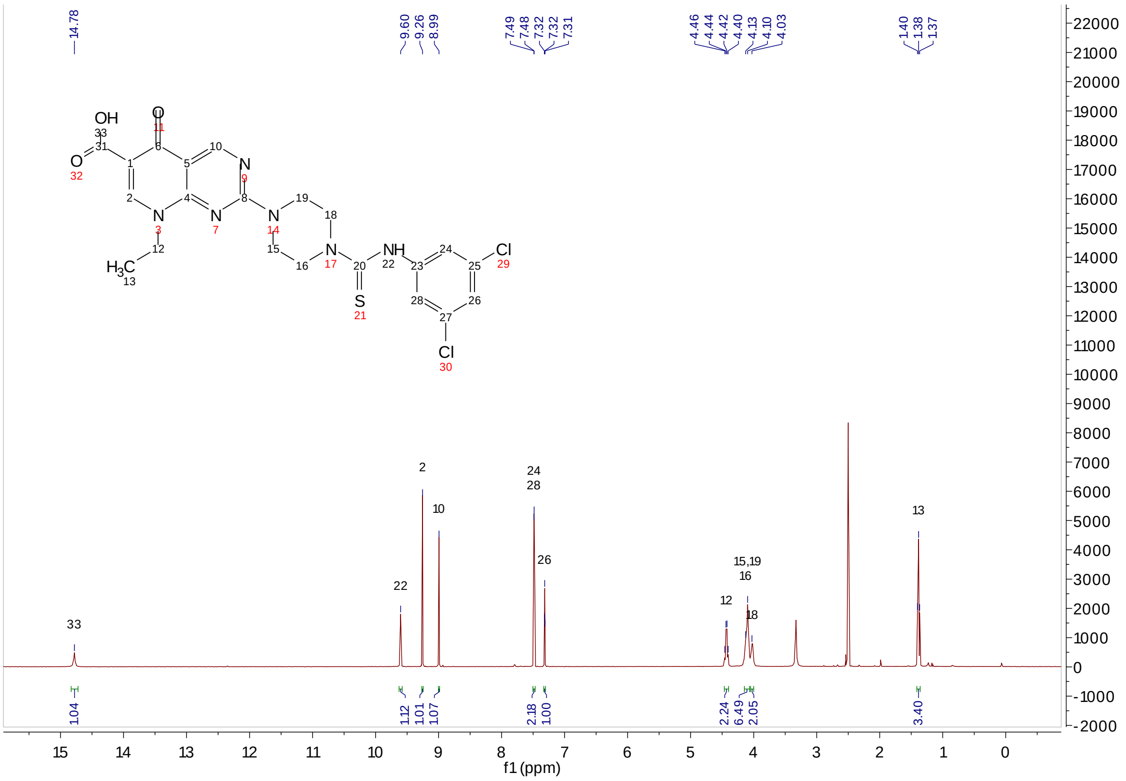

Figure S14. ^{1}**H NMR (400 MHz, DMSO-*d*$_6$) at 298 K of 6.**

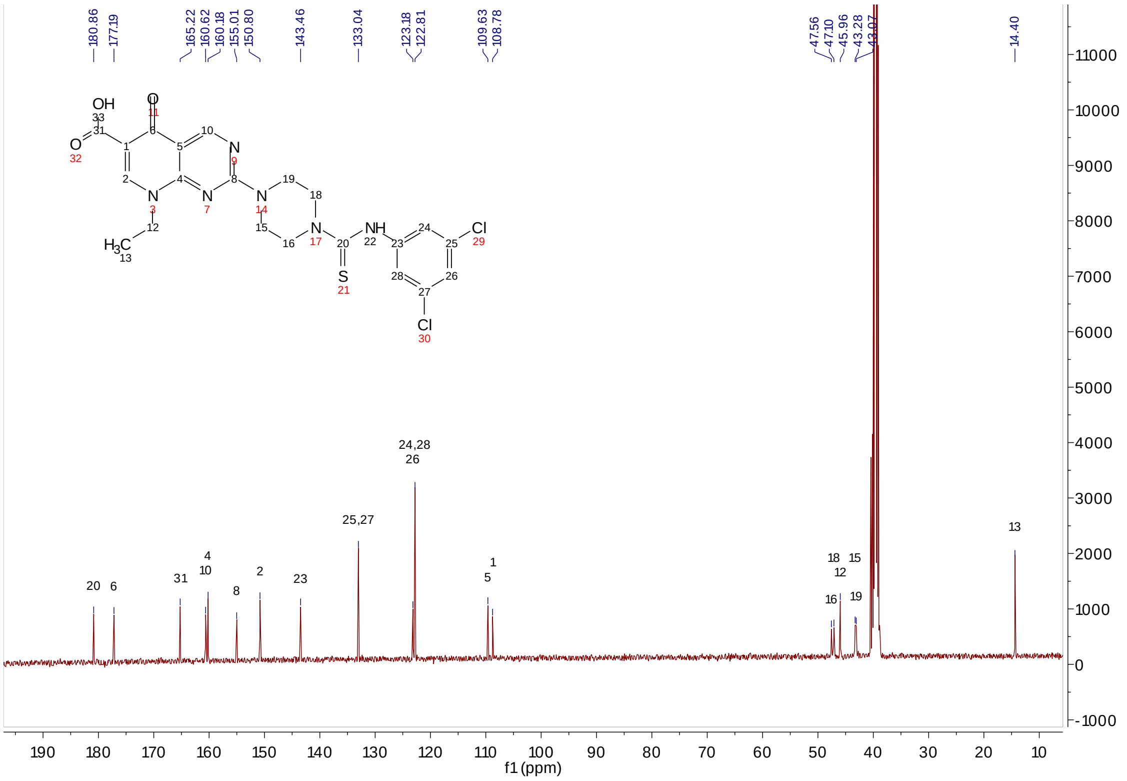

Figure S15. ^{13}**C NMR (400 MHz, DMSO-*d*$_6$) at 298 K of 6.**

2.5.7. 1-Cyclopropyl-6-fluoro-4-oxo-7-(4-((3-(trifluoromethyl)phenyl)carbamothioyl)piperazin-1-yl)-1,4-dihydroquinoline-3-carboxylic acid (7).

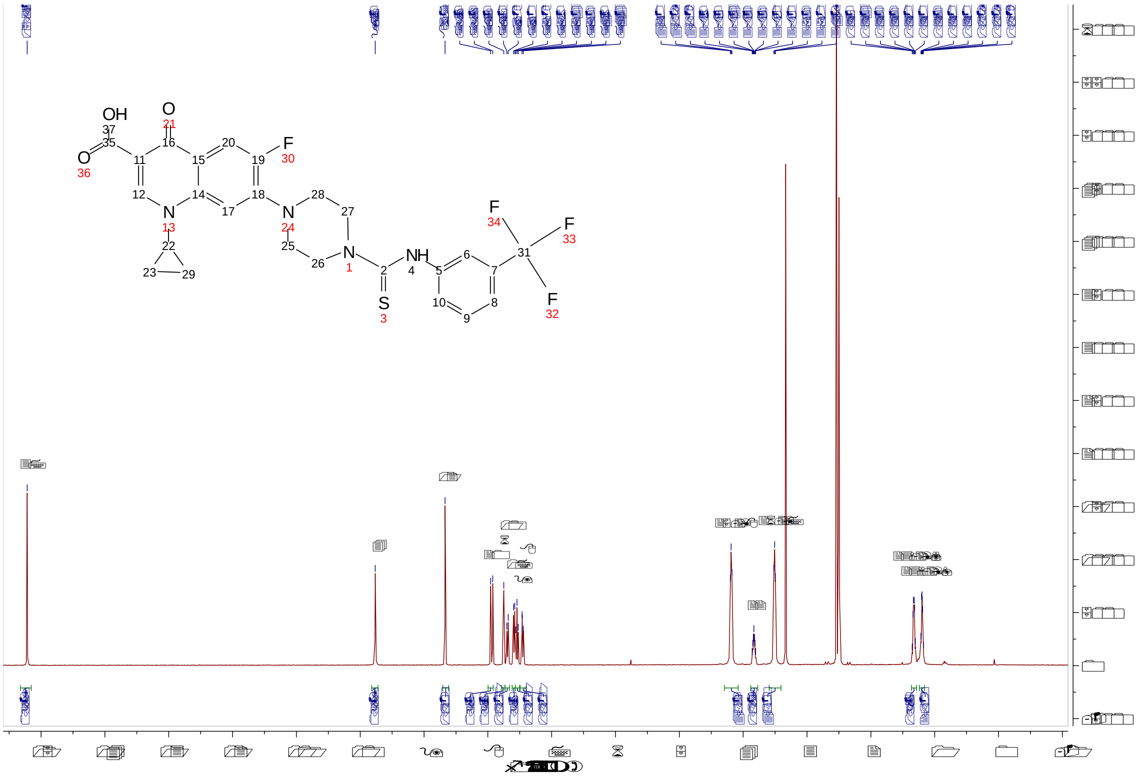

Figure S16. **^{1}H NMR (400 MHz, DMSO-*d*6) at 298 K of 7.**

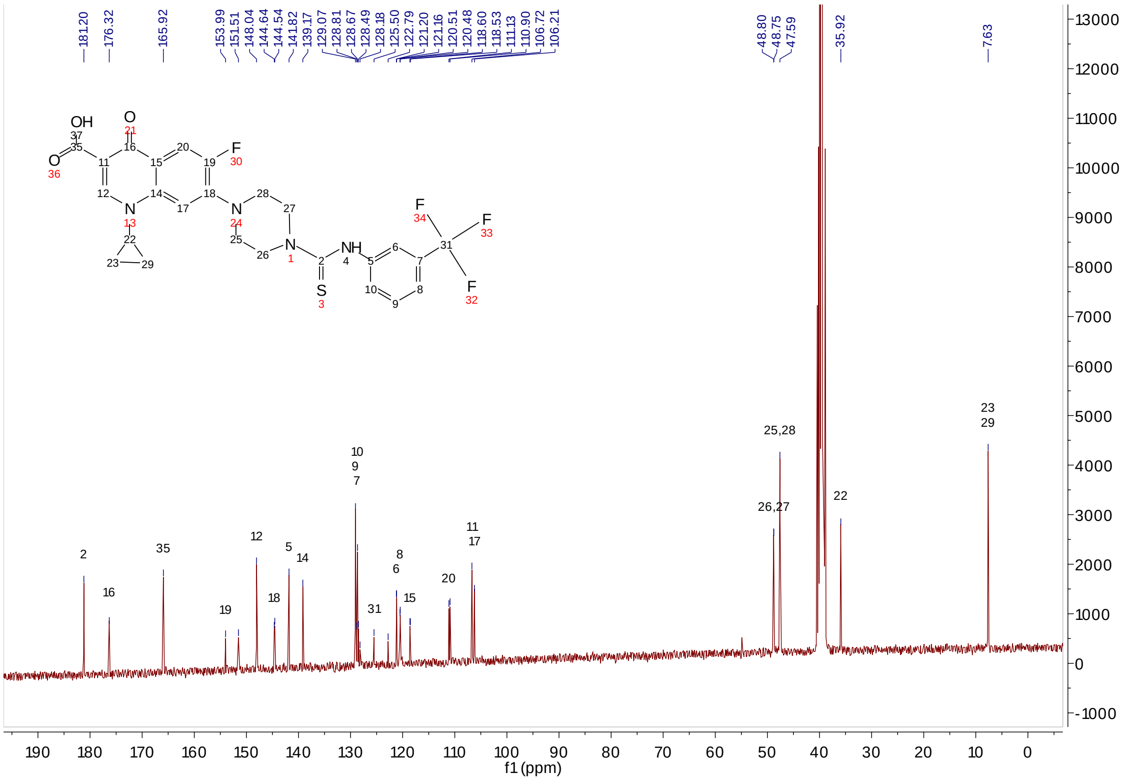

Figure S17. **^{13}C NMR (400 MHz, DMSO-*d*6) at 298 K of 7.**

2.5.8. 1-Cyclopropyl-6-fluoro-7-(4-((3-fluorophenyl)carbamothioyl)piperazin-1-yl)-4-oxo-1,4-dihydroquinoline-3-carboxylic acid (8).

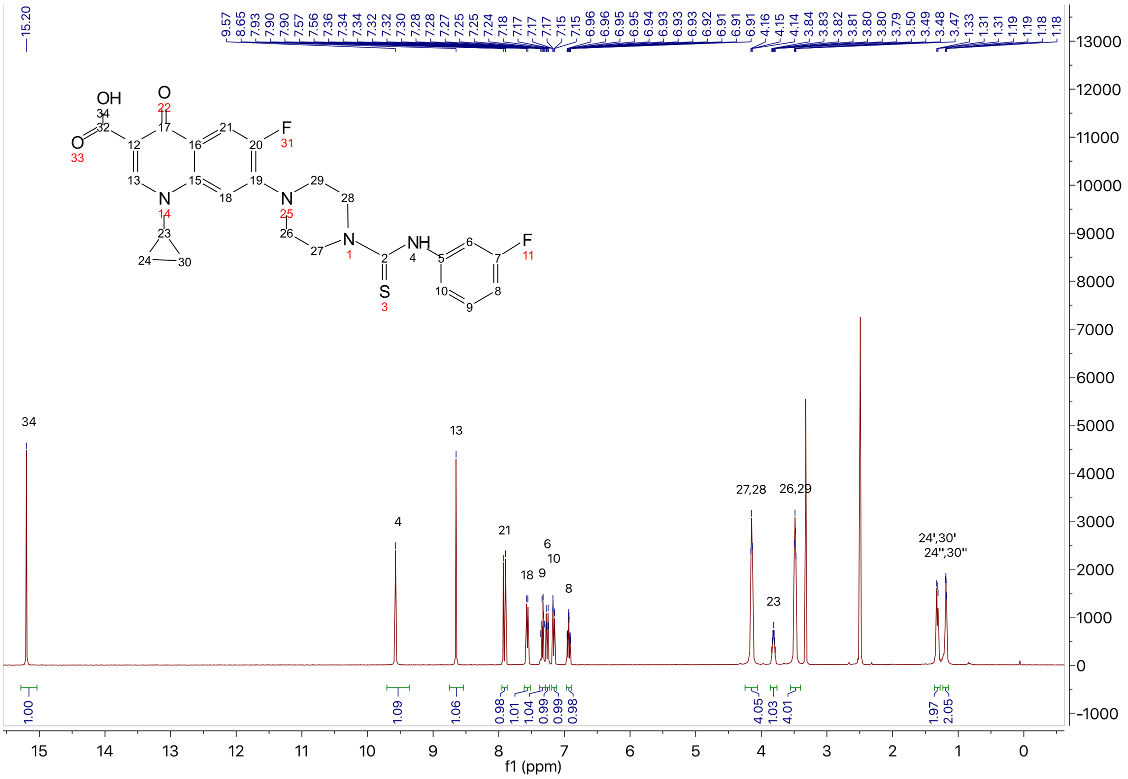

Figure S18. ^{1}H NMR (400 MHz, DMSO-d_6) at 298 K of 8.

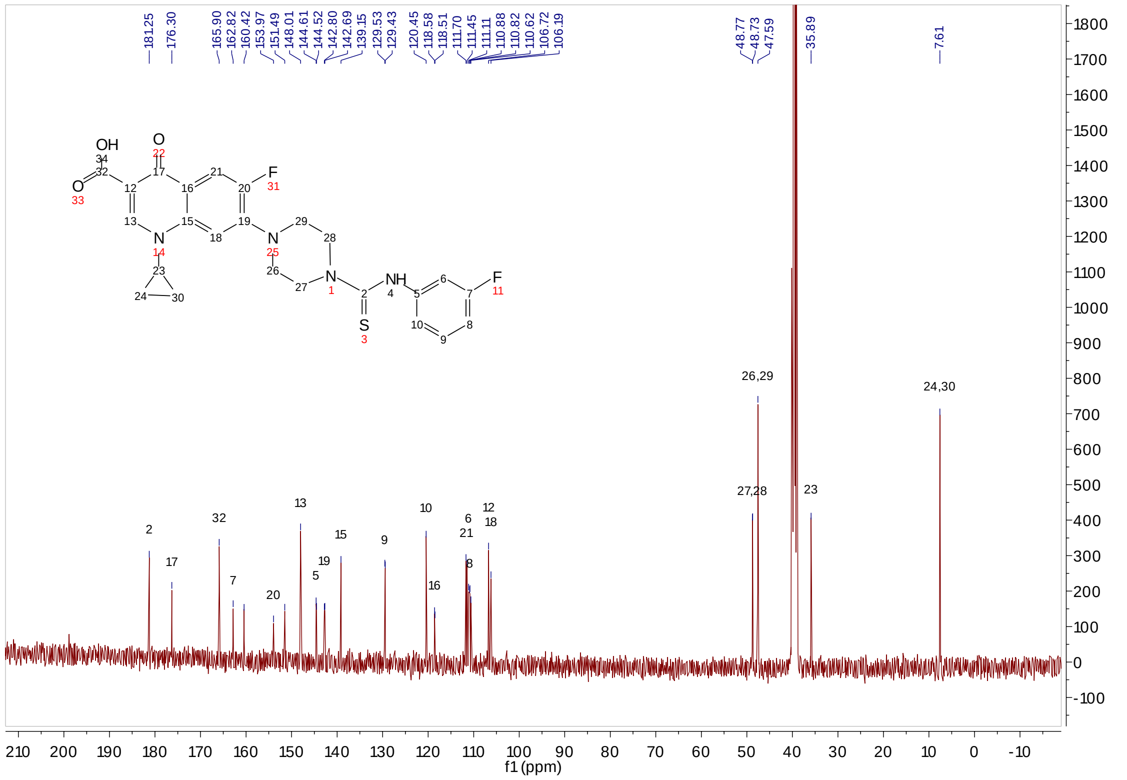

Figure S19. ^{13}C NMR (400 MHz, DMSO-d_6) at 298 K of 8.

2.5.9. 7-(4-((3,5-Bis(trifluoromethyl)phenyl)carbamothioyl)piperazin-1-yl)-1-cyclopropyl-6-fluoro-4-oxo-1,4-dihydroquinoline-3-carboxylic acid (9).

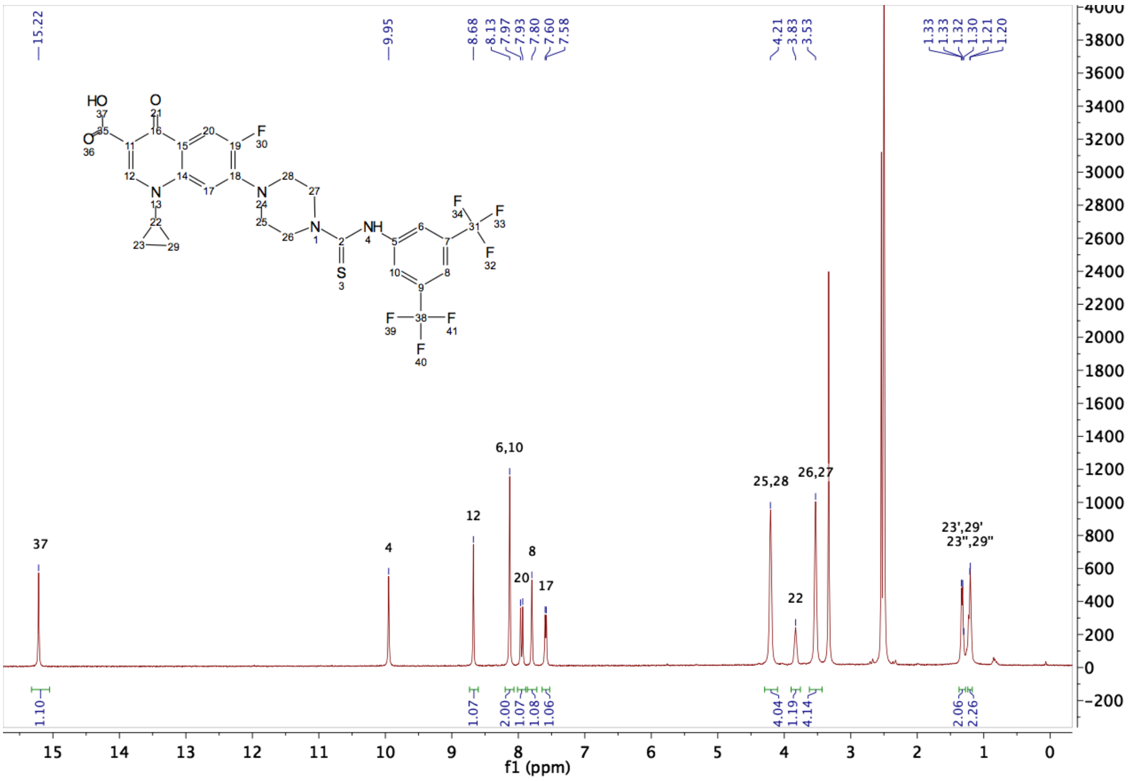

Figure S20. ^{1}H NMR (400 MHz, DMSO-d_6) at 298 K of 9.

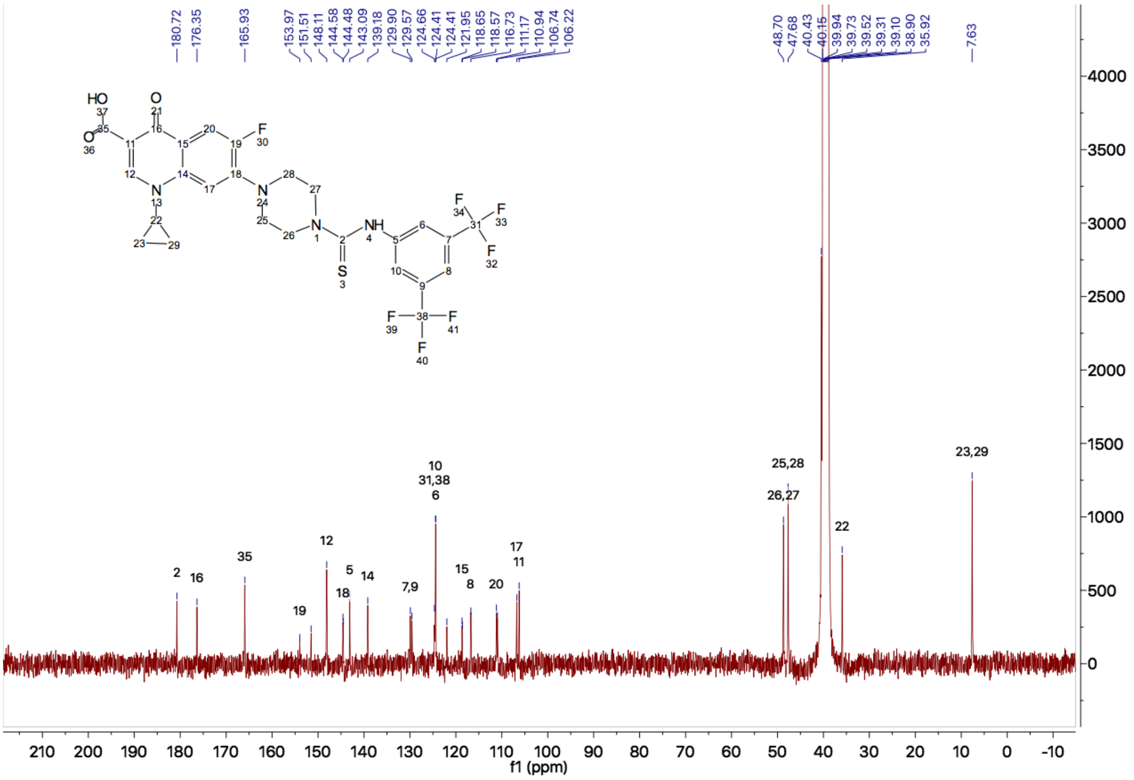

Figure S21. ^{13}C NMR (400 MHz, DMSO-d_6) at 298 K of 9.

2.5.10. 1-Cyclopropyl-6-fluoro-4-oxo-7-(4-((4-(trifluoromethyl)phenyl)carbamothioyl)piperazin-1-yl)-1,4-dihydroquinoline-3-carboxylic acid (10).

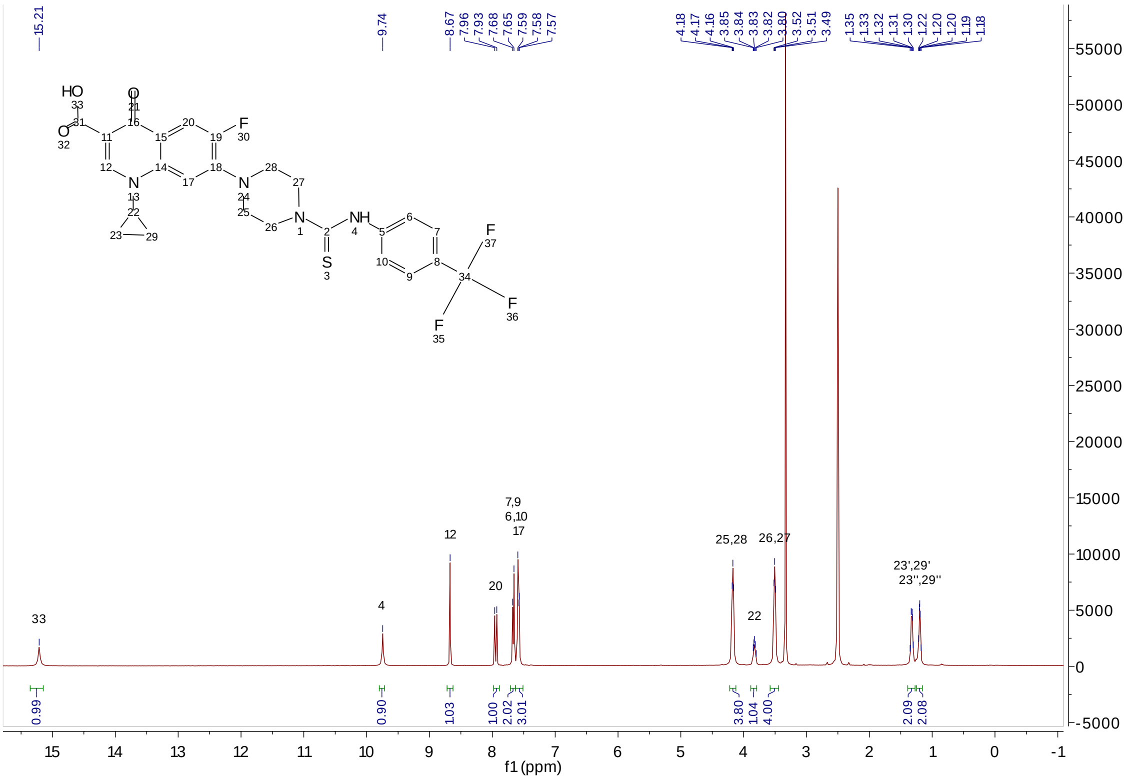

Figure S22. ^{1}H NMR (400 MHz, DMSO-d_6) at 298 K of 10.

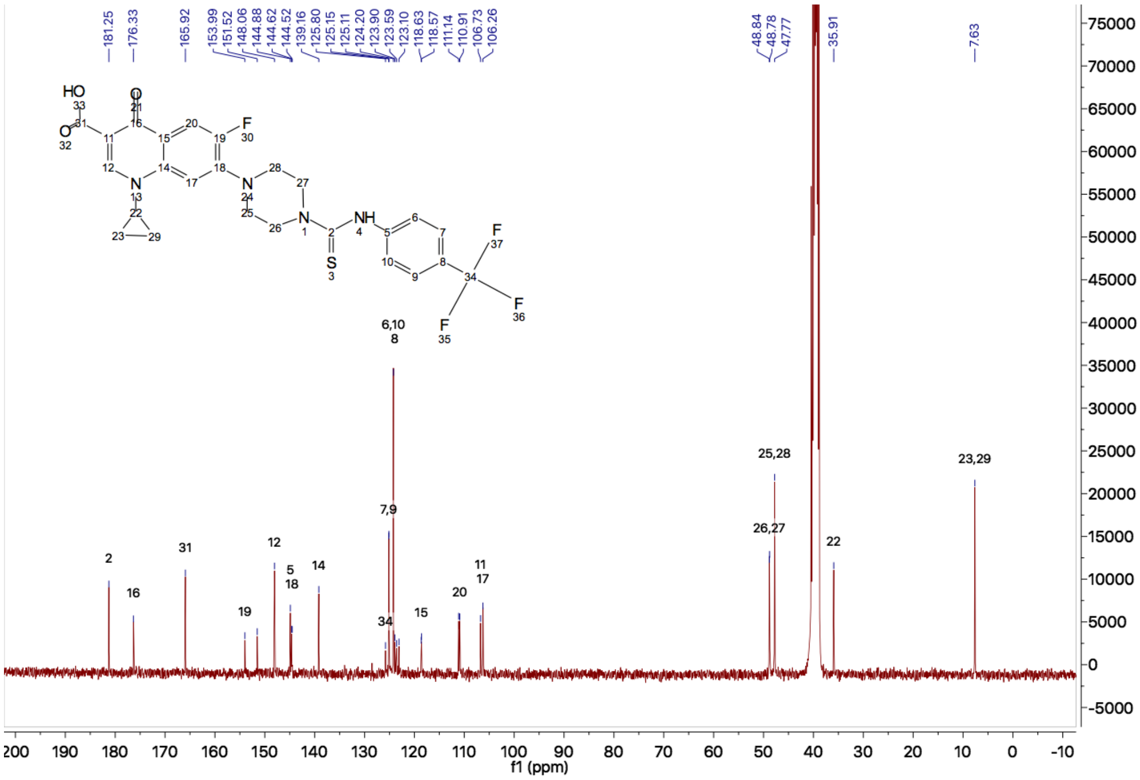

Figure S23. ^{13}C NMR (400 MHz, DMSO-d_6) at 298 K of 10.

2.5.11. 7-(4-((3-Chlorophenyl)carbamothioyl)piperazin-1-yl)-1-cyclopropyl-6-fluoro-4-oxo-1,4-dihydroquinoline-3-carboxylic acid (11).

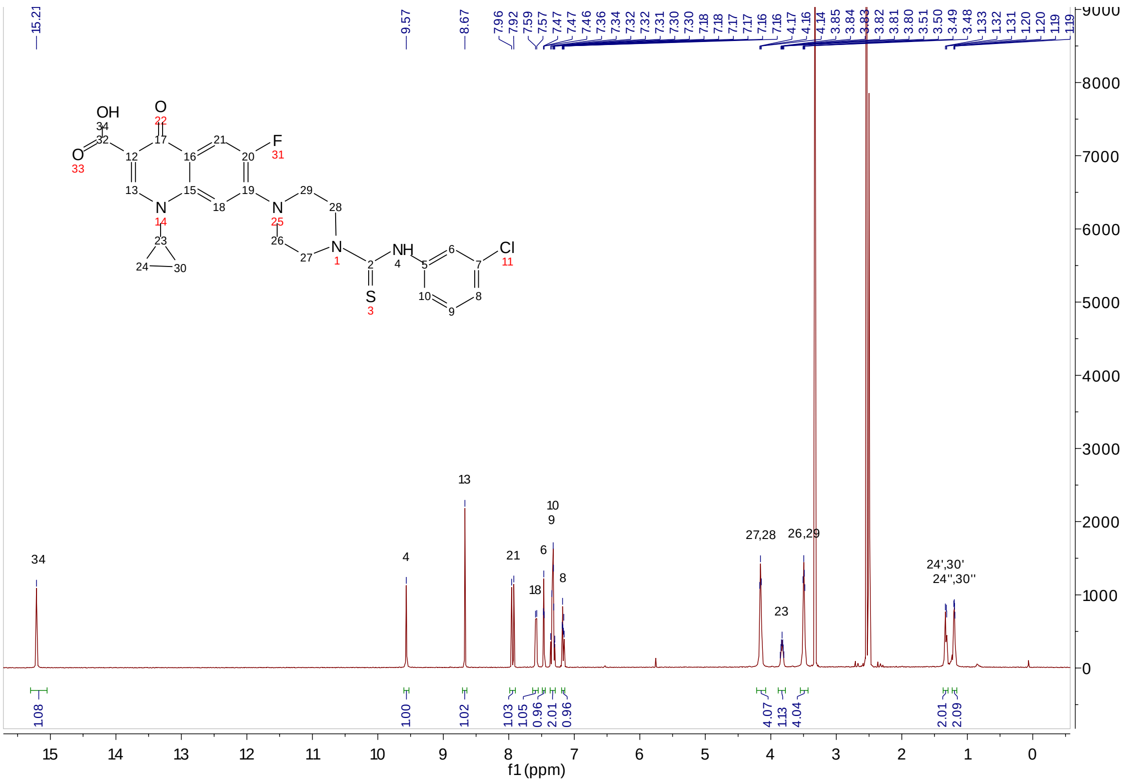

Figure S24. **^{1}H NMR (400 MHz, DMSO-*d*6) at 298 K of 11.**

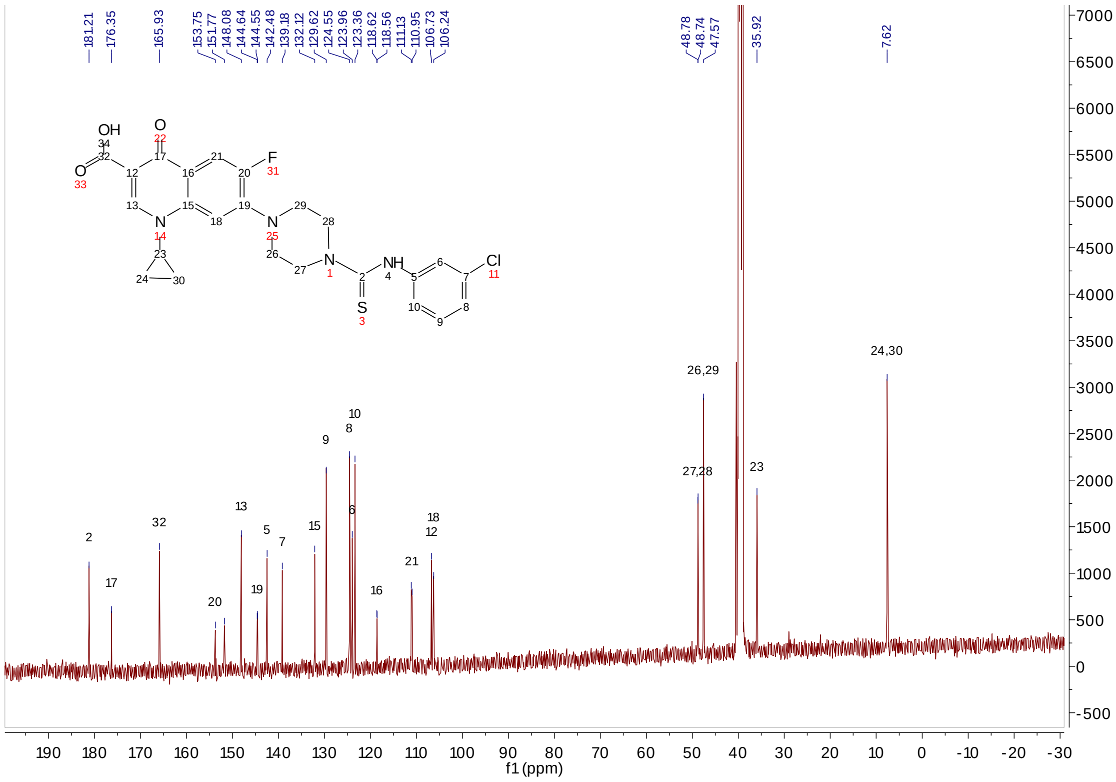

Figure S25. **^{13}C NMR (500 MHz, DMSO-*d*6) at 298 K of 11.**

2.5.12. 1-Cyclopropyl-7-(4-((3,5-dichlorophenyl)carbamothioyl)piperazin-1-yl)-6-fluoro-4-oxo-1,4-dihydroquinoline-3-carboxylic acid (12).

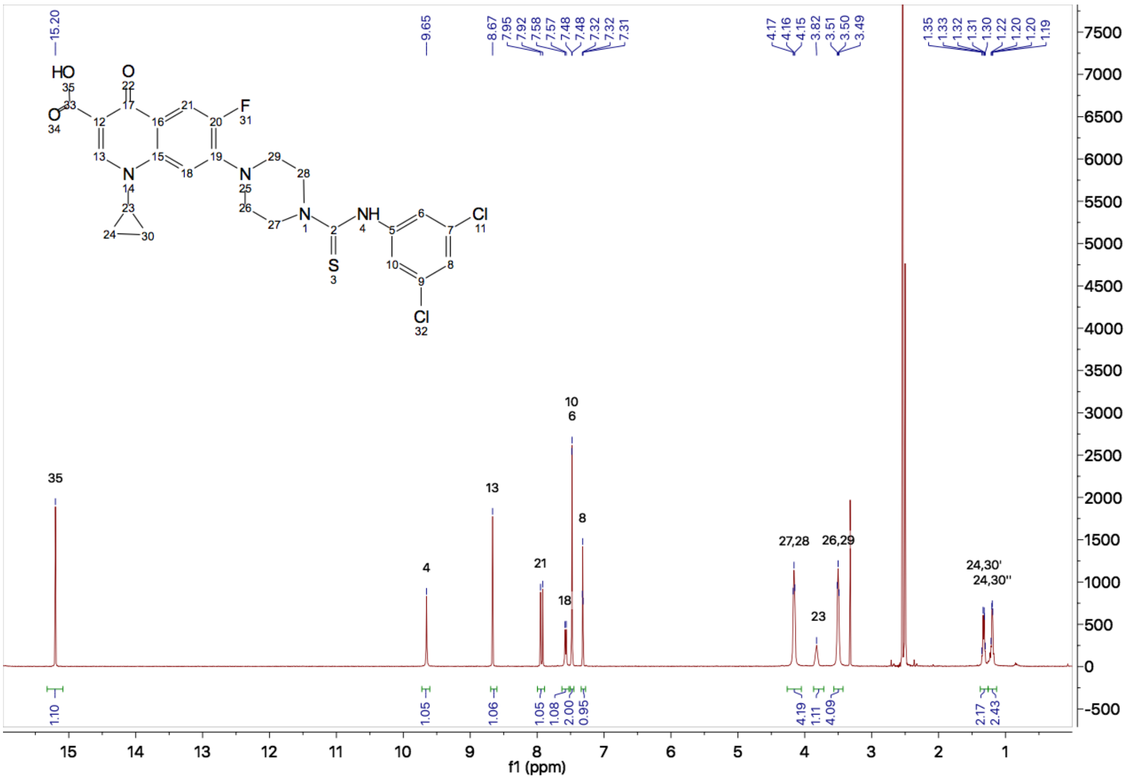

Figure S26. **^{1}H NMR (400 MHz, DMSO-*d*$_6$) at 298 K of 12.**

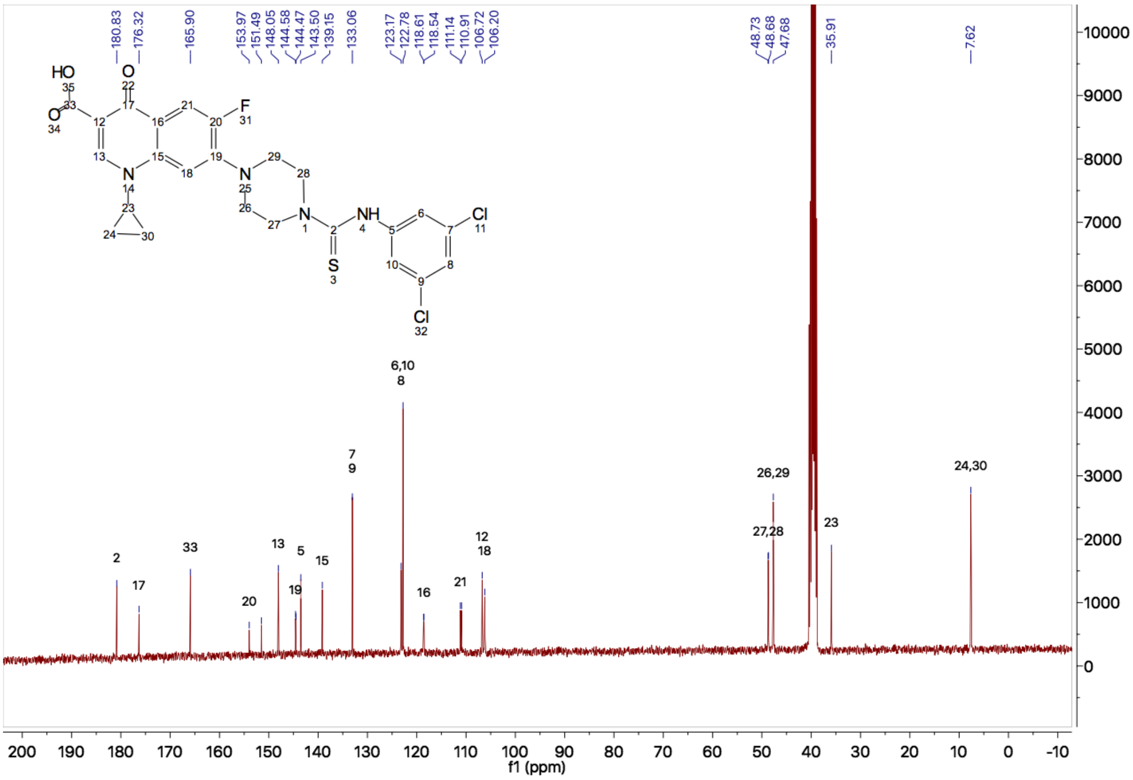

Figure S27. **^{13}C NMR (400 MHz, DMSO-*d*$_6$) at 298 K of 12.**

2.5.13. 1-Cyclopropyl-6-fluoro-7-(4-((3-nitrophenyl)carbamothioyl)piperazin-1-yl)-4-oxo-1,4-dihydroquinoline-3-carboxylic acid (13).

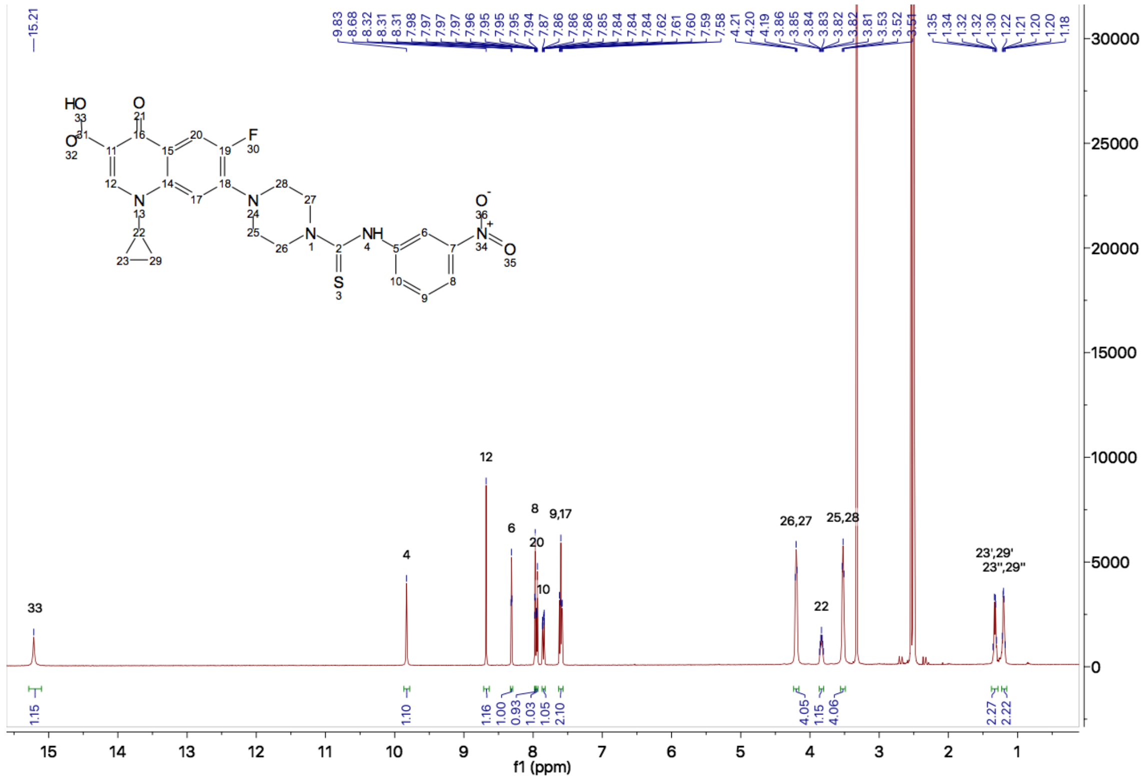

Figure S28. ^{1}H NMR (400 MHz, DMSO-d_6) at 298 K of 13.

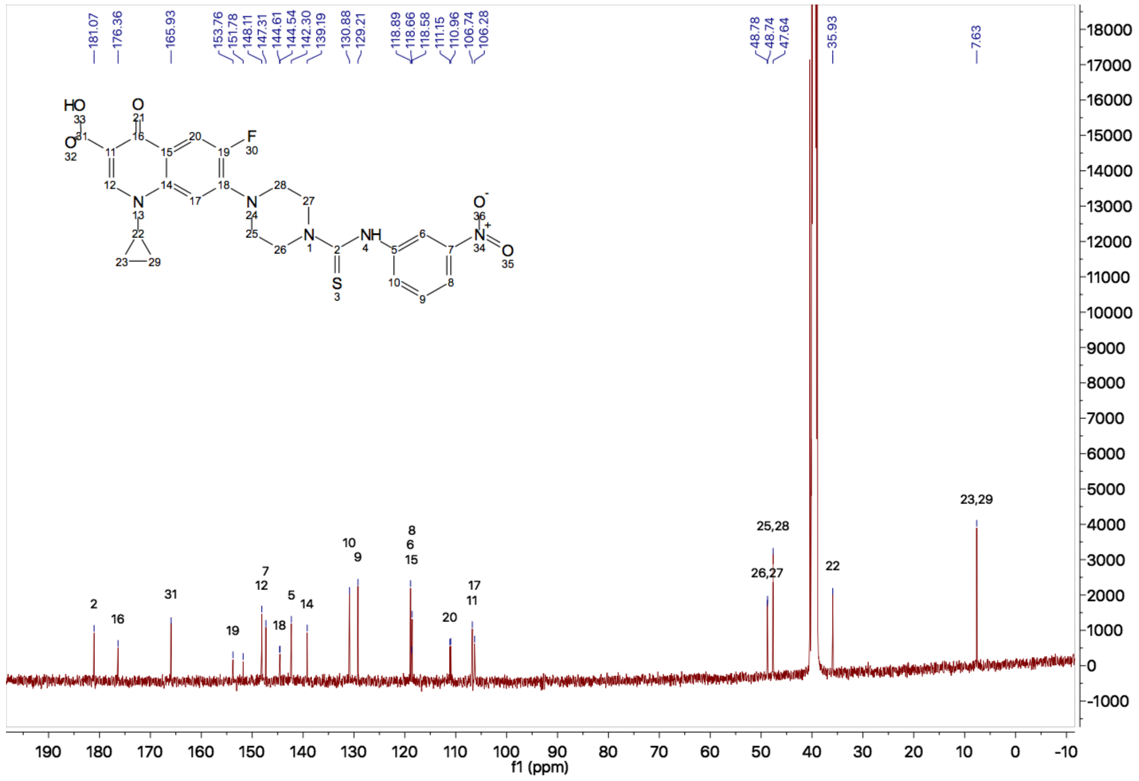

Figure S29. ^{13}C NMR (500 MHz, DMSO-d_6) at 298 K of 13.

2.5.14. 1-Cyclopropyl-6-fluoro-7-(4-((4-nitrophenyl)carbamothioyl)piperazin-1-yl)-4-oxo-1,4-dihydroquinoline-3-carboxylic acid (14).

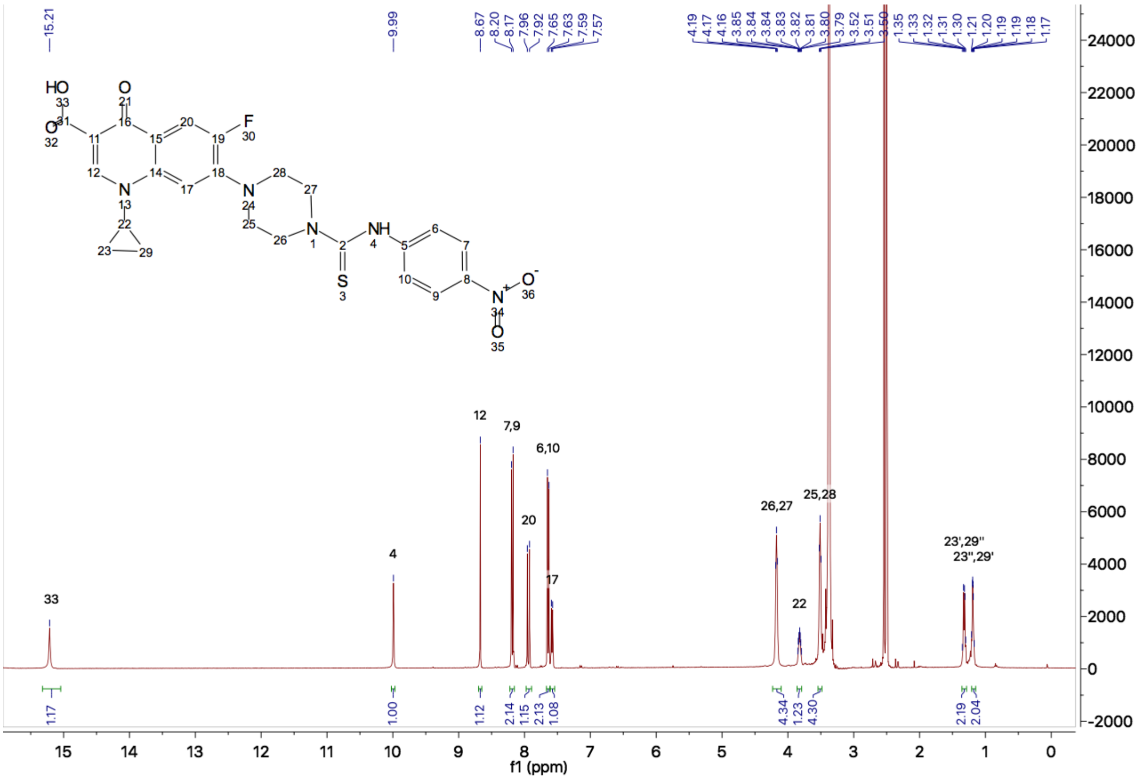

Figure S30. ^{1}H NMR (400 MHz, DMSO-d_6) at 298 K of 14.

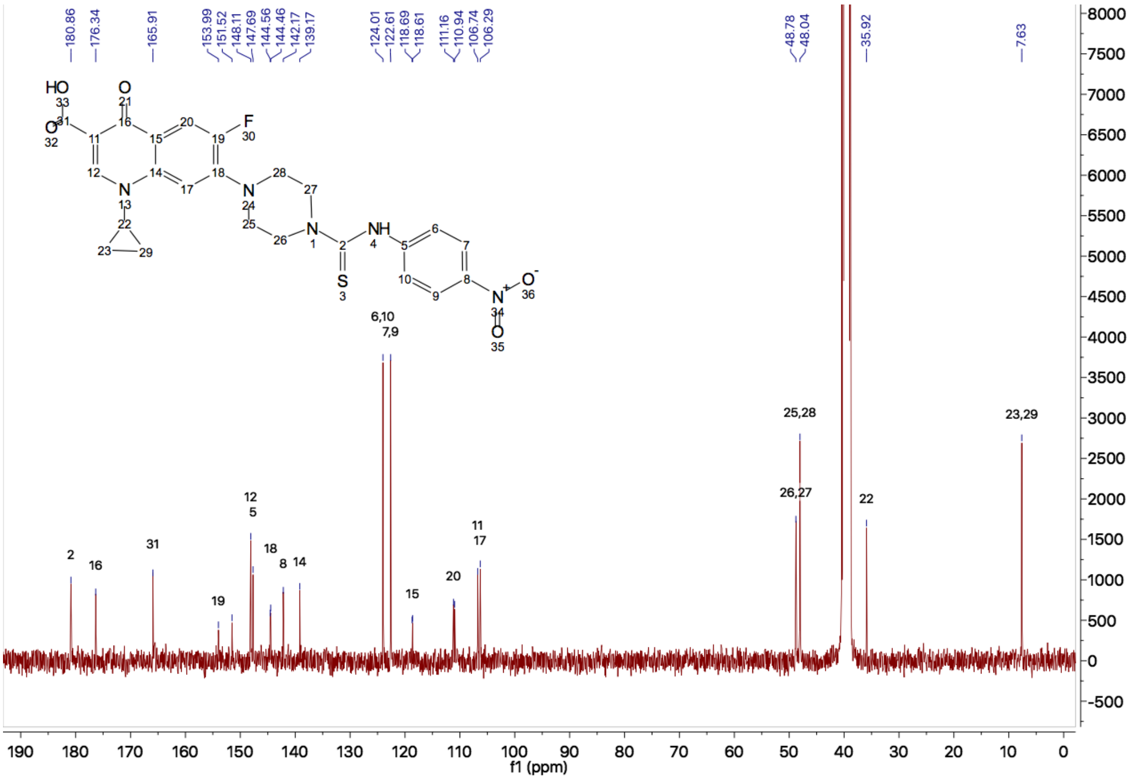

Figure S31. ^{13}C NMR (400 MHz, DMSO-d_6) at 298 K of 14.

2.5.15. 8-Ethyl-5-oxo-2-(4-(phenylcarbamoyl)piperazin-1-yl)-5,8-dihydropyrido[2,3-*d*]pyrimidine-6-carboxylic acid (15).

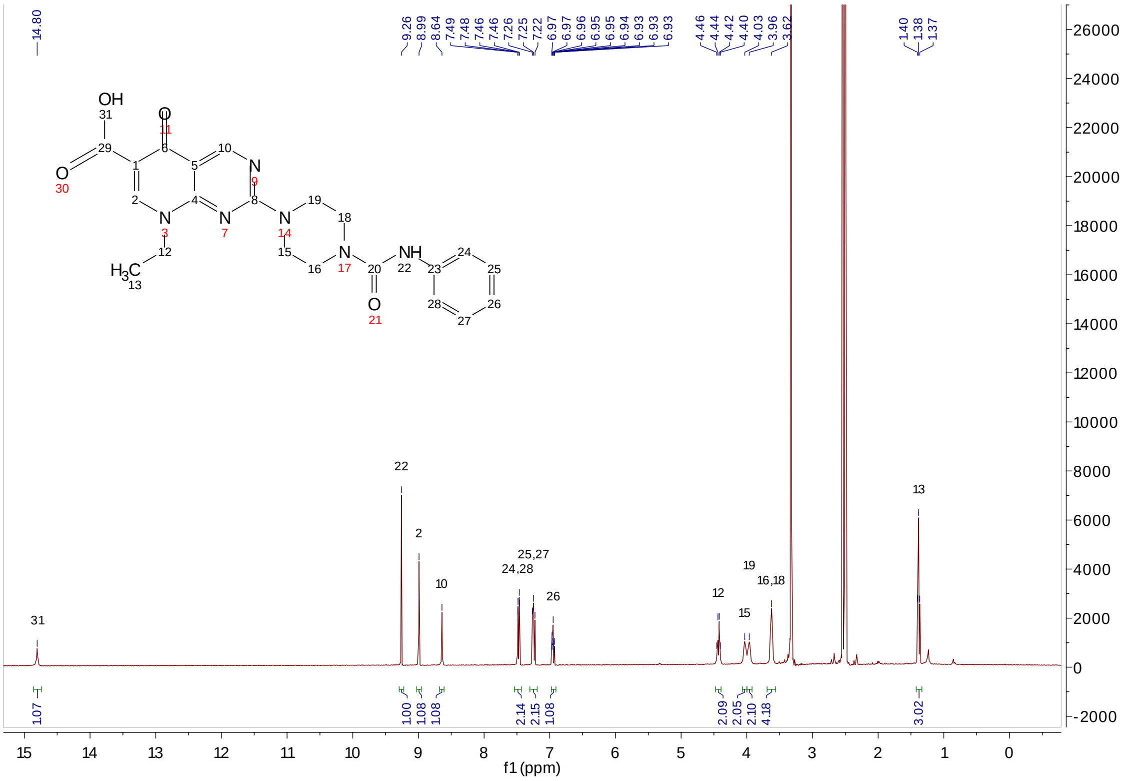

Figure S32. ^{1}**H NMR (400 MHz, DMSO-*d*$_6$) at 298 K of 15.**

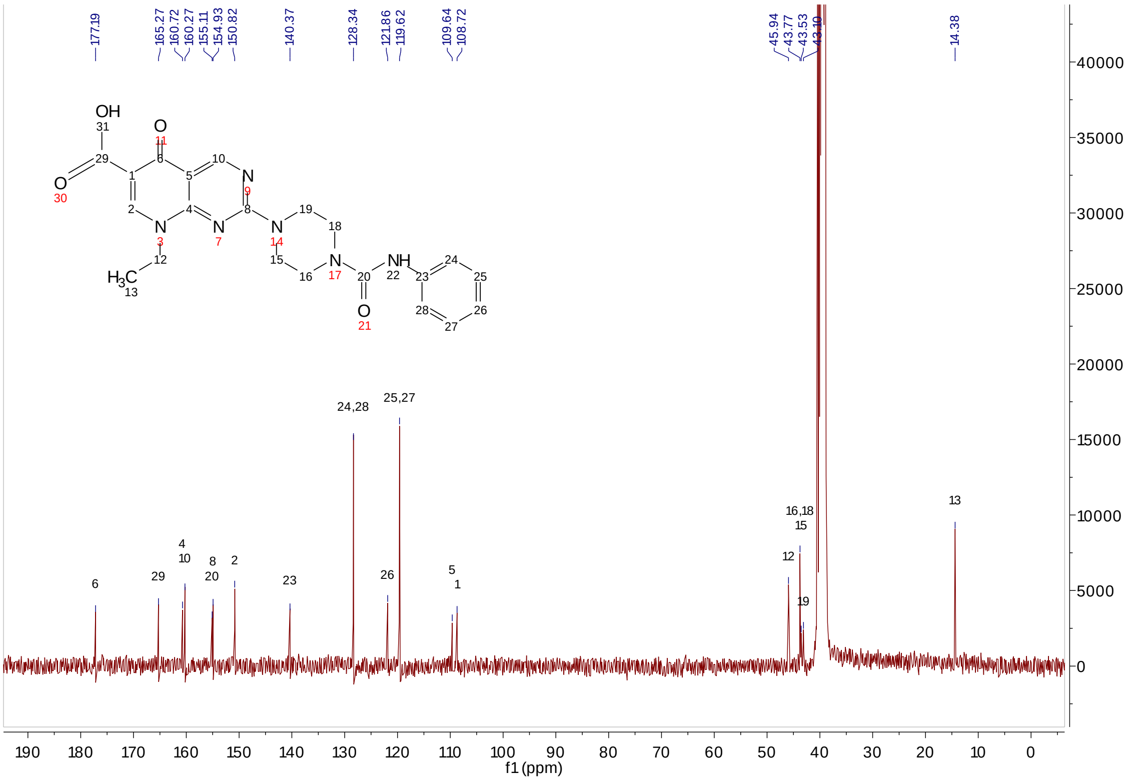

Figure S33. ^{13}**C NMR (400 MHz, DMSO-*d*$_6$) at 298 K of 15.**

2.5.16. 1-Cyclopropyl-6-fluoro-7-(3-methyl-4-((4-(trifluoromethyl)phenyl)carbamothioyl)piperazin-1-yl)-4-oxo-1,4-dihydroquinoline-3-carboxylic acid (16).

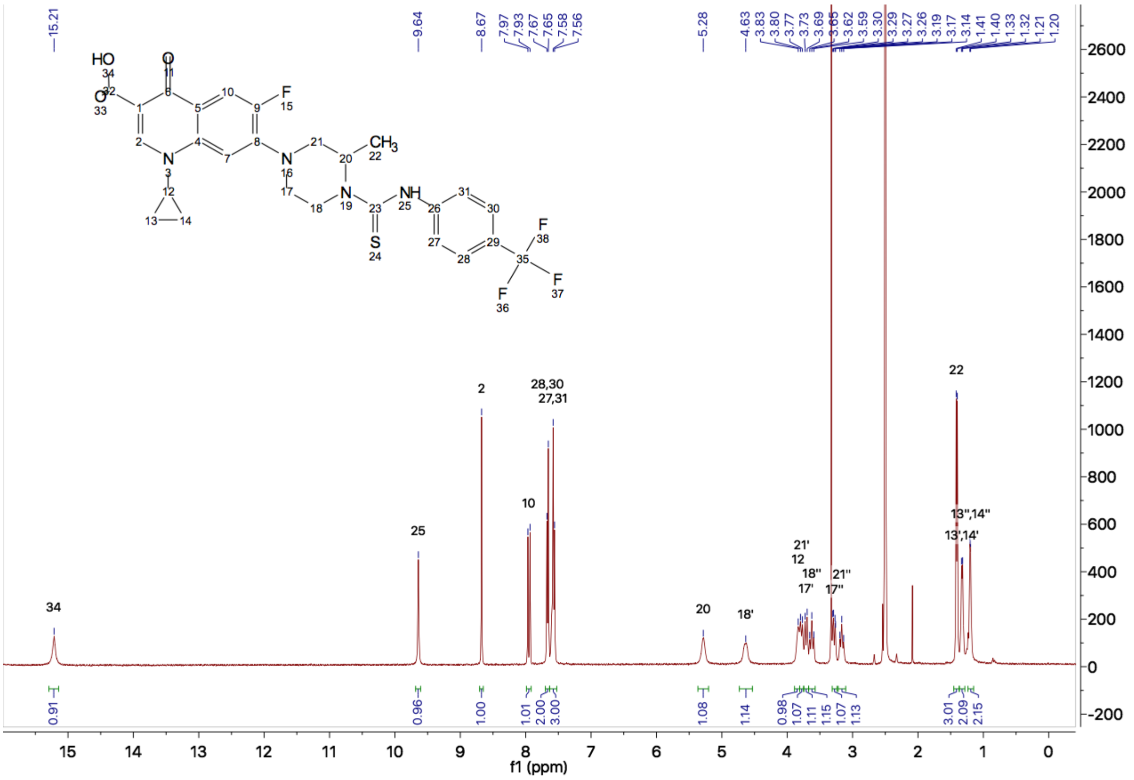

Figure S34. **^{13}H NMR (400 MHz, DMSO-d_6) at 298 K of 16.**

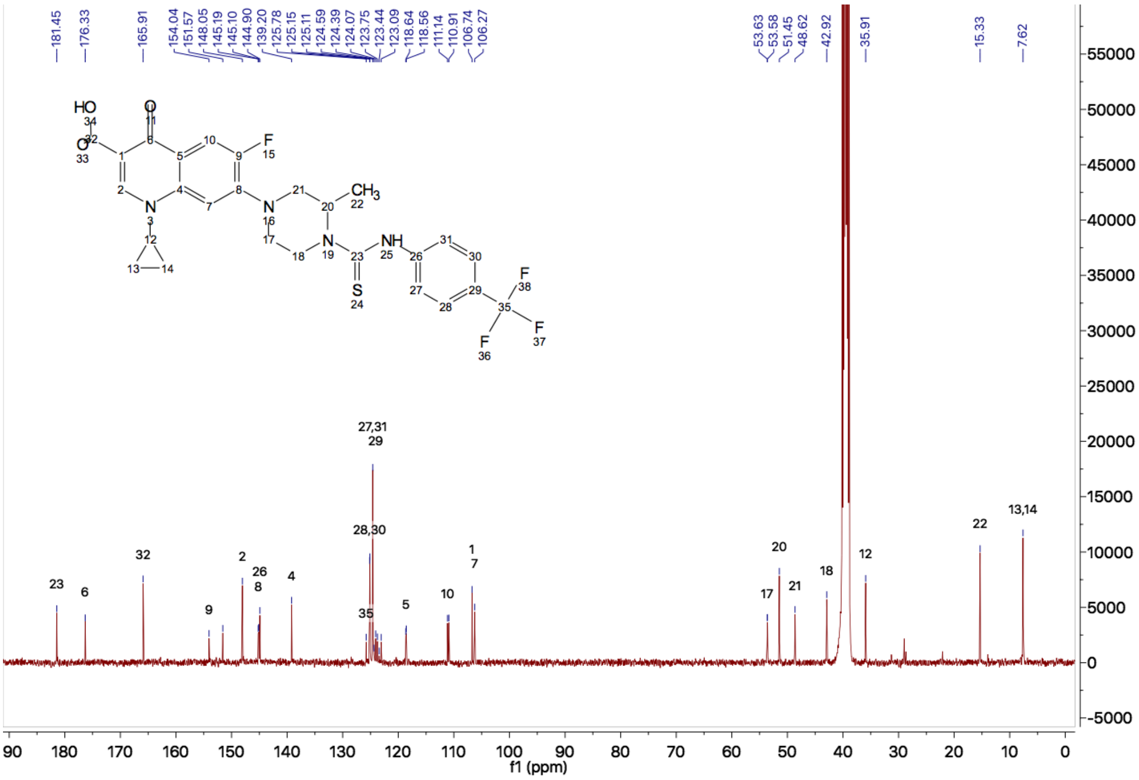

Figure S35. **^{13}C NMR (400 MHz, DMSO-d_6) at 298 K of 16.**

2.5.17. 1-Cyclopropyl-6-fluoro-4-oxo-7-(3-(3-(4-(trifluoromethyl)phenyl)thioureido)pyrrolidin-1-yl)-1,4-dihydroquinoline-3-carboxylic acid (17).

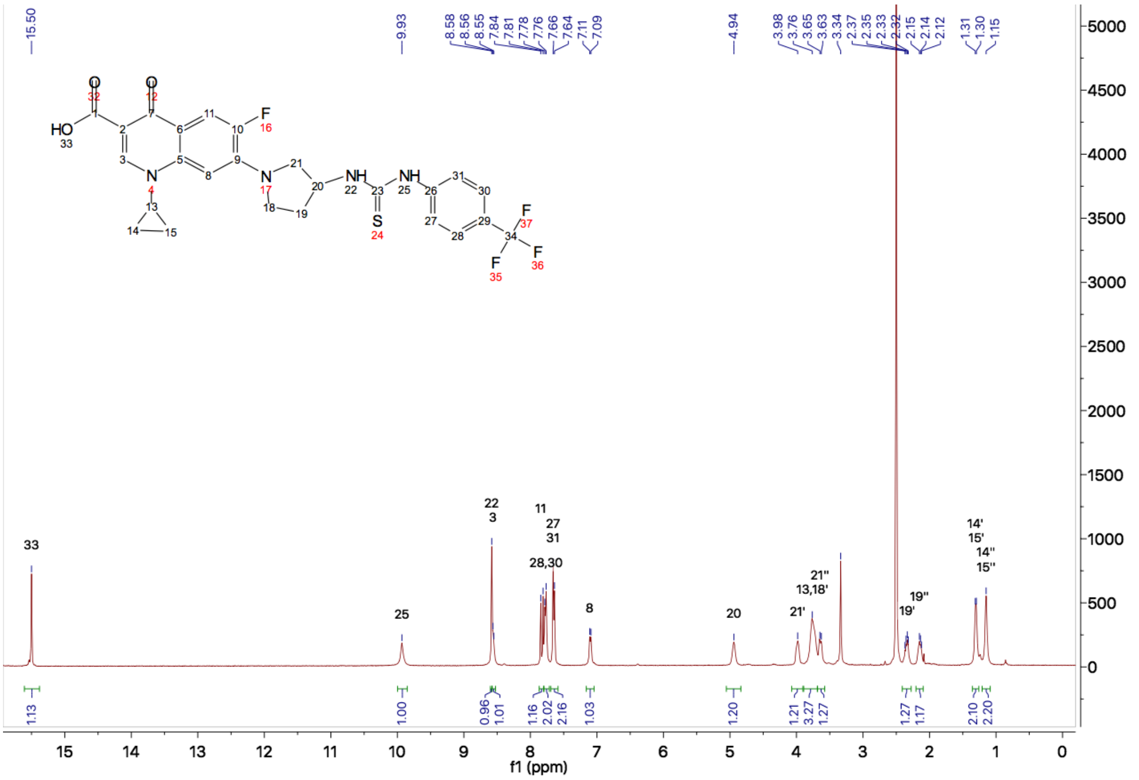

Figure S36. **^{1}H NMR (400 MHz, DMSO-*d*$_6$) at 298 K of 17.**

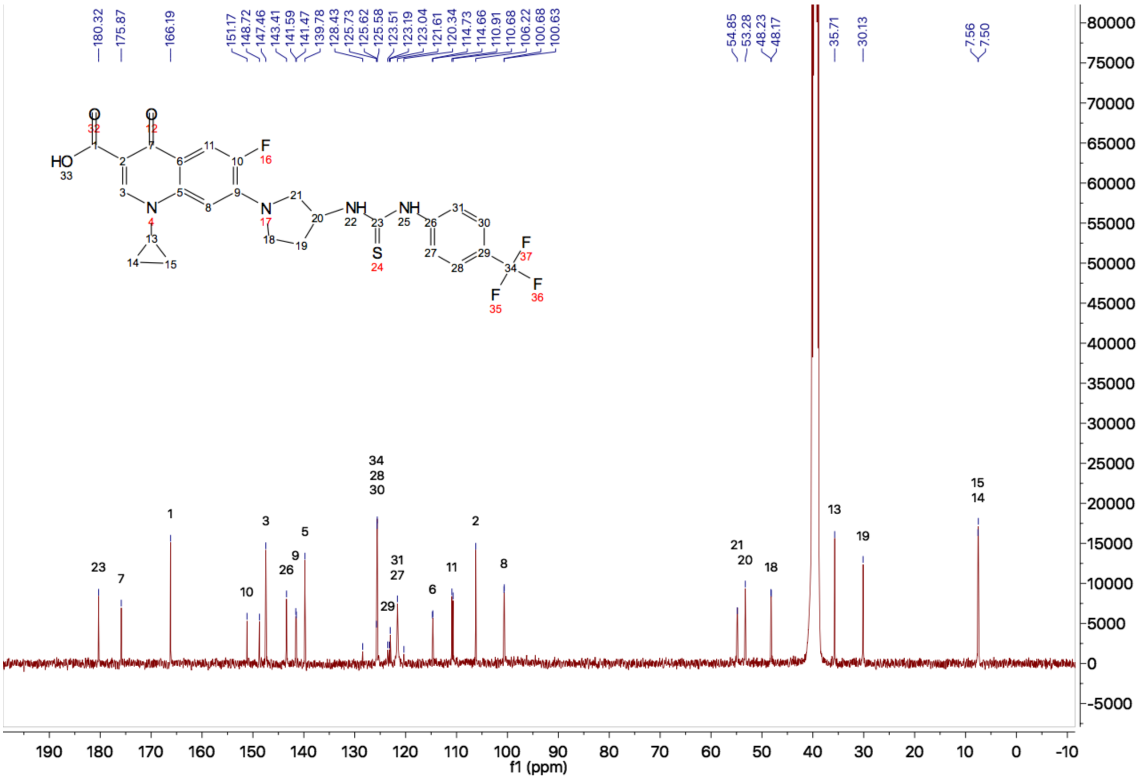

Figure S37. **^{13}C NMR (400 MHz, DMSO-*d*$_6$) at 298 K of 17.**

2.5.18. 1-Cyclopropyl-6-fluoro-4-oxo-7-((2-(3-(4-(trifluoromethyl)phenyl)thioureido)ethyl)amino)-1,4-dihydroquinoline-3-carboxylic acid (18).

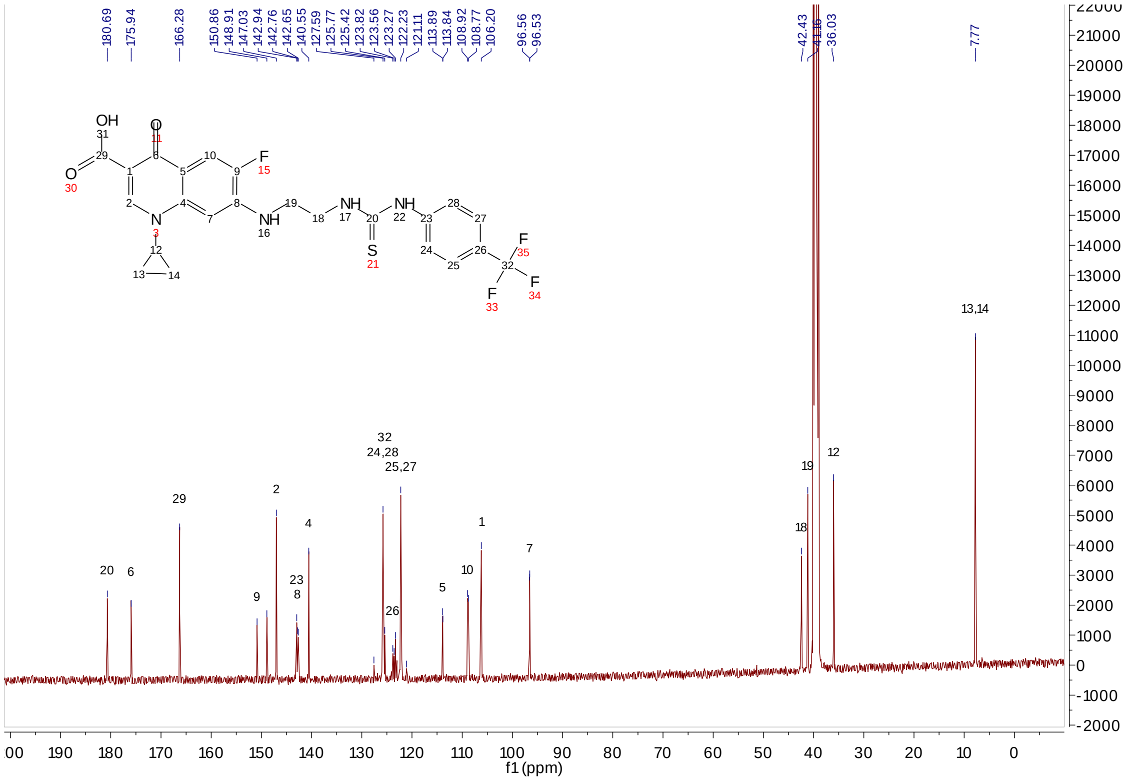

Figure S38. ^{1}H NMR (500 MHz, DMSO-d_6) at 298 K of 18.

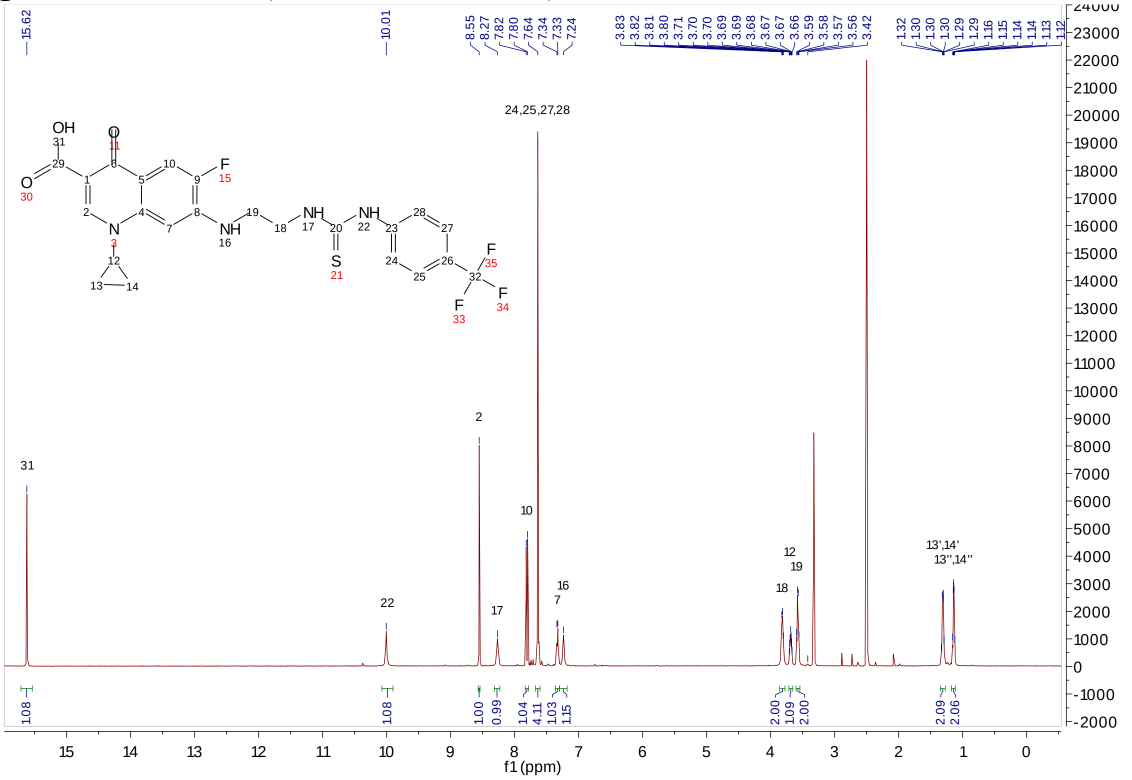

Figure S39. ^{13}C NMR (500 MHz, DMSO-d_6) at 298 K of 18.

2.5.19. *tert*-Butyl (2-(1-cyclopropyl-6-fluoro-4-oxo-7-(4-((4-(trifluoromethyl)phenyl)carbamothioyl)piperazin-1-yl)-1,4-dihydroquinoline-3-carboxamido)ethyl)carbamate (19).

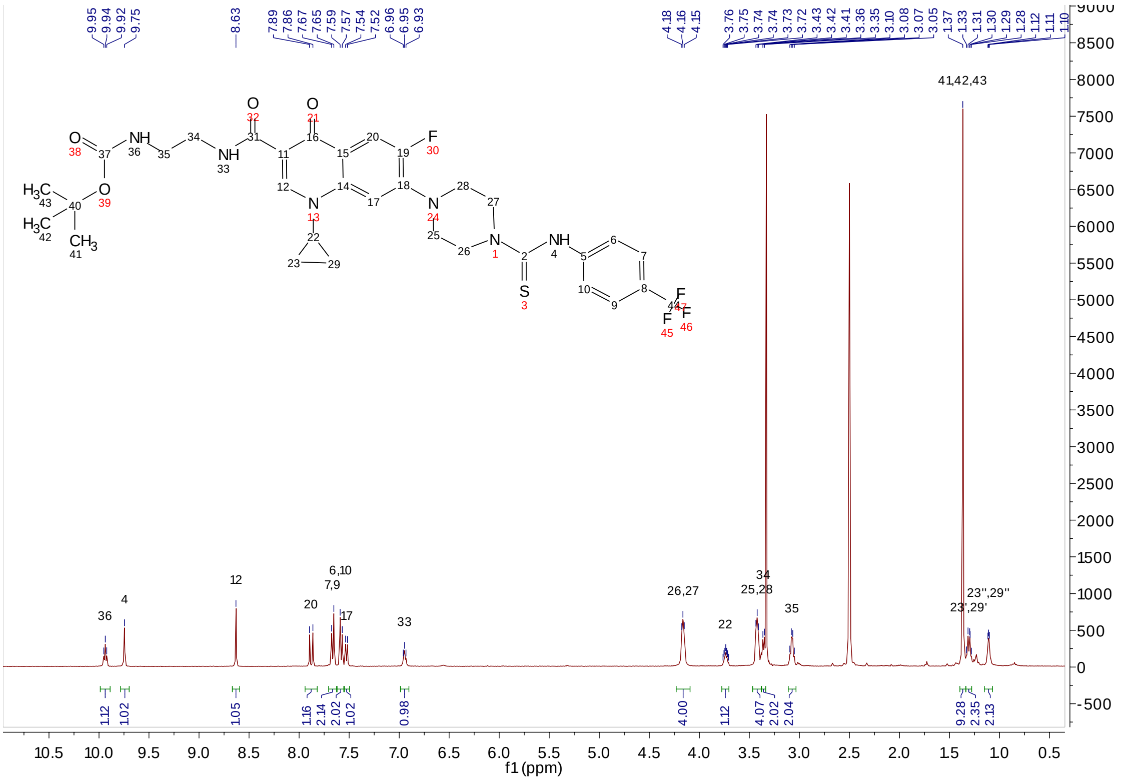

Figure S40. ^{1}**H NMR (400 MHz, DMSO-*d*$_6$) at 298 K of 19.**

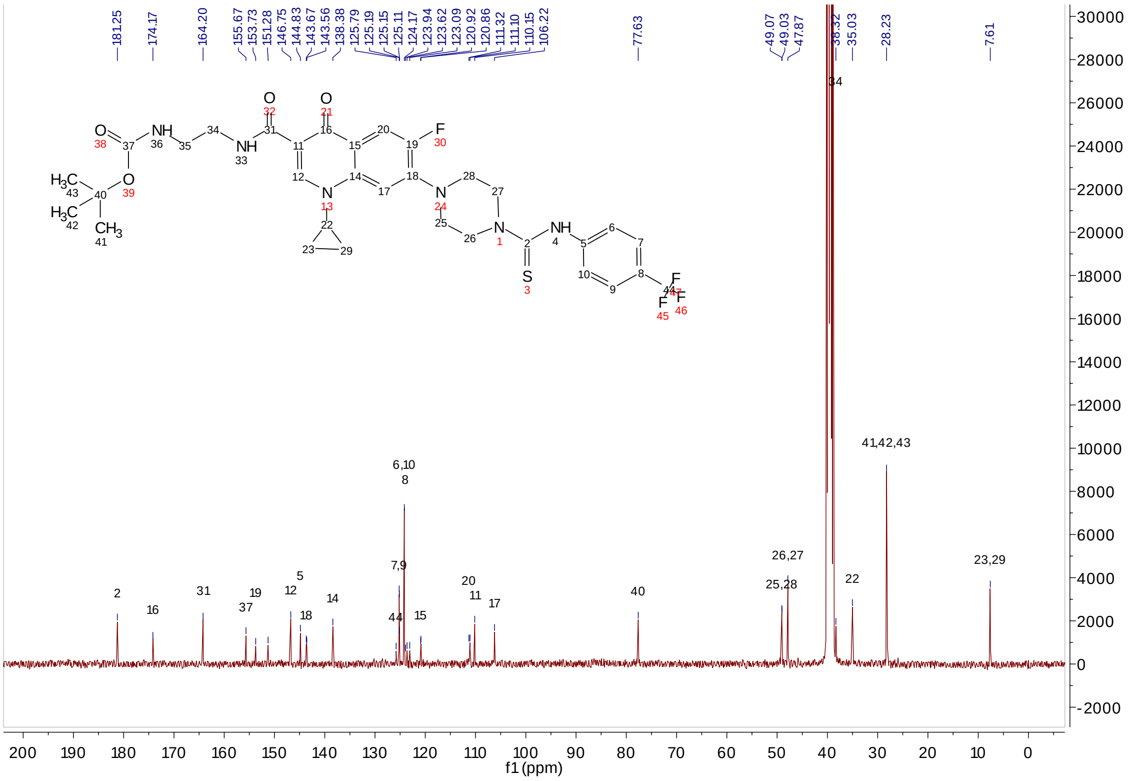

Figure S41. ^{13}**C NMR (400 MHz, DMSO-*d*$_6$) at 298 K of 19.**

2.5.20. ***N*-(2a-Aminoethyl)-1-cyclopropyl-6-fluoro-4-oxo-7-(4-((4-(trifluoromethyl)phenyl)carbamothioyl)piperazin-1-yl)-1,4-dihydroquinoline-3-carboxamide (20).**

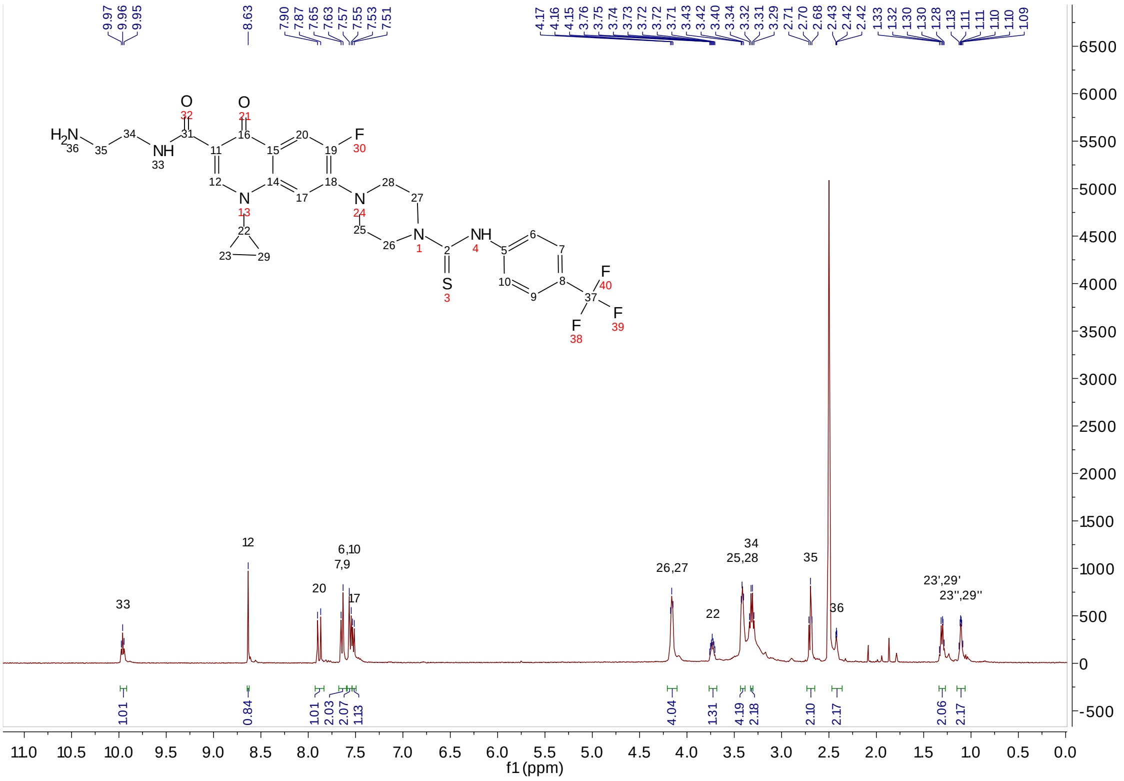

Figure S42. ^{1}**H NMR (400 MHz, DMSO-*d*$_6$) at 298 K of 20.**

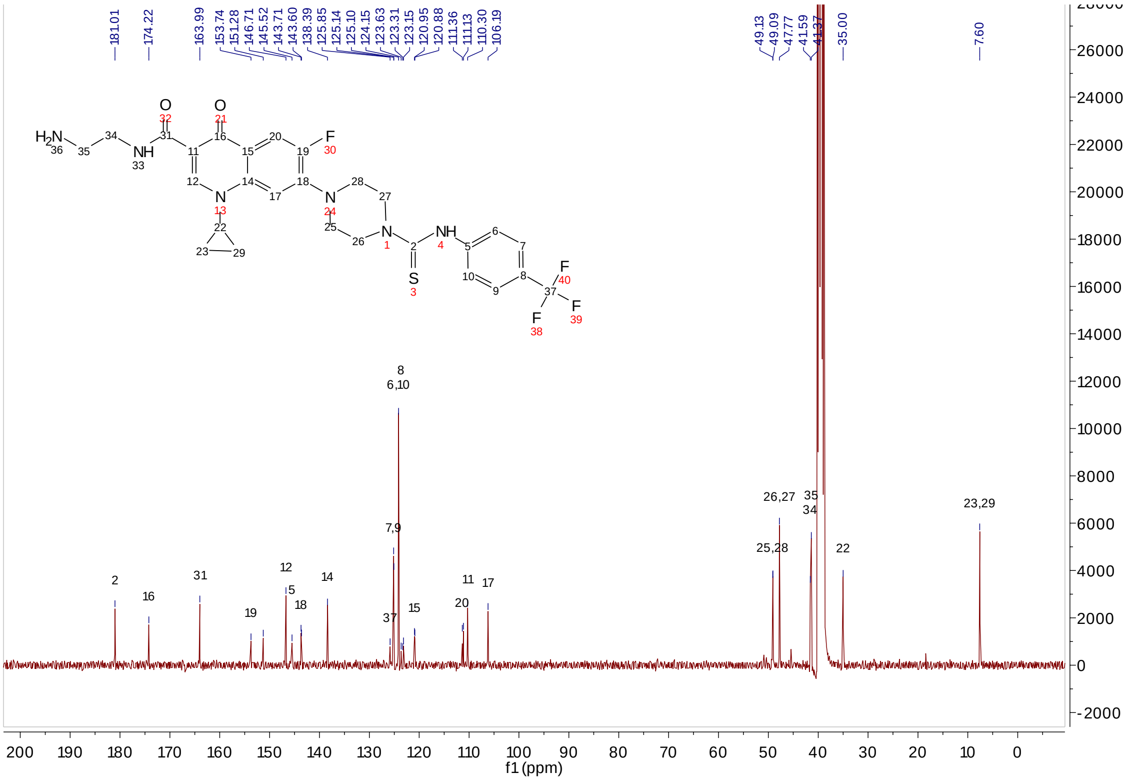

Figure S43. ^{13}**C NMR (400 MHz, DMSO-*d*$_6$) at 298 K of 20.**

1-Cyclopropyl-6-fluoro-4-oxo-7-(4-(phenylcarbamoyl)piperazin-1-yl)-1,4-dihydroquinoline-3-carboxylic acid (21)

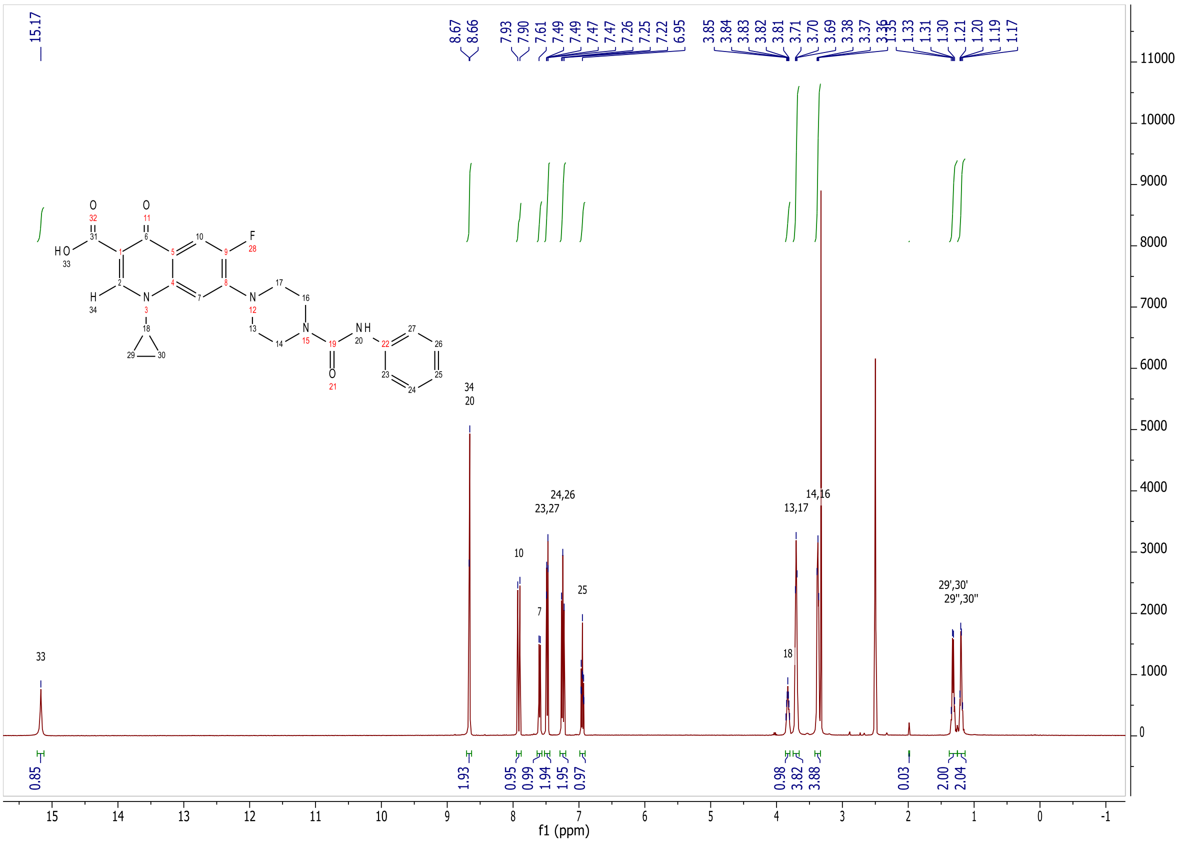

Figure S44. ^{1}H NMR (400 MHz, DMSO-d_6) at 298 K of 21.

***tert*-Butyl (2-(1-cyclopropyl-6-fluoro-4-oxo-7-(4-(phenylcarbamoyl)piperazin-1-yl)-1,4-dihydroquinoline-3-carboxamido)ethyl)carbamate (22)**

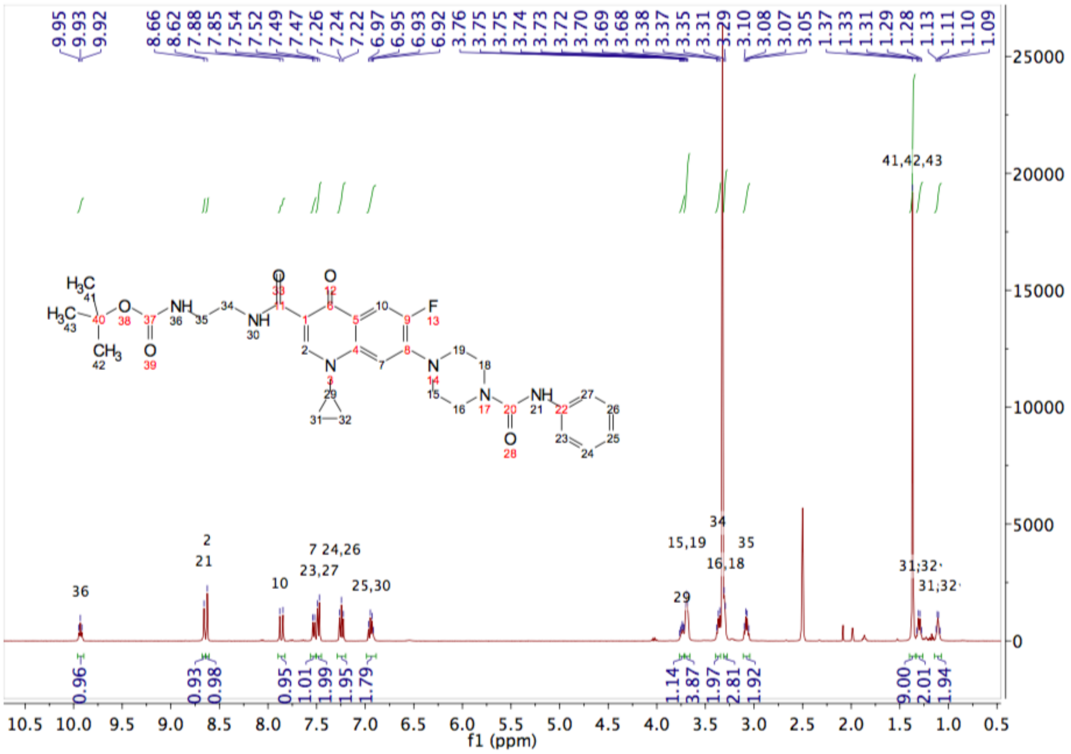

Figure S45. ^{1}H NMR (400 MHz, DMSO-d_6) at 298 K of 22.

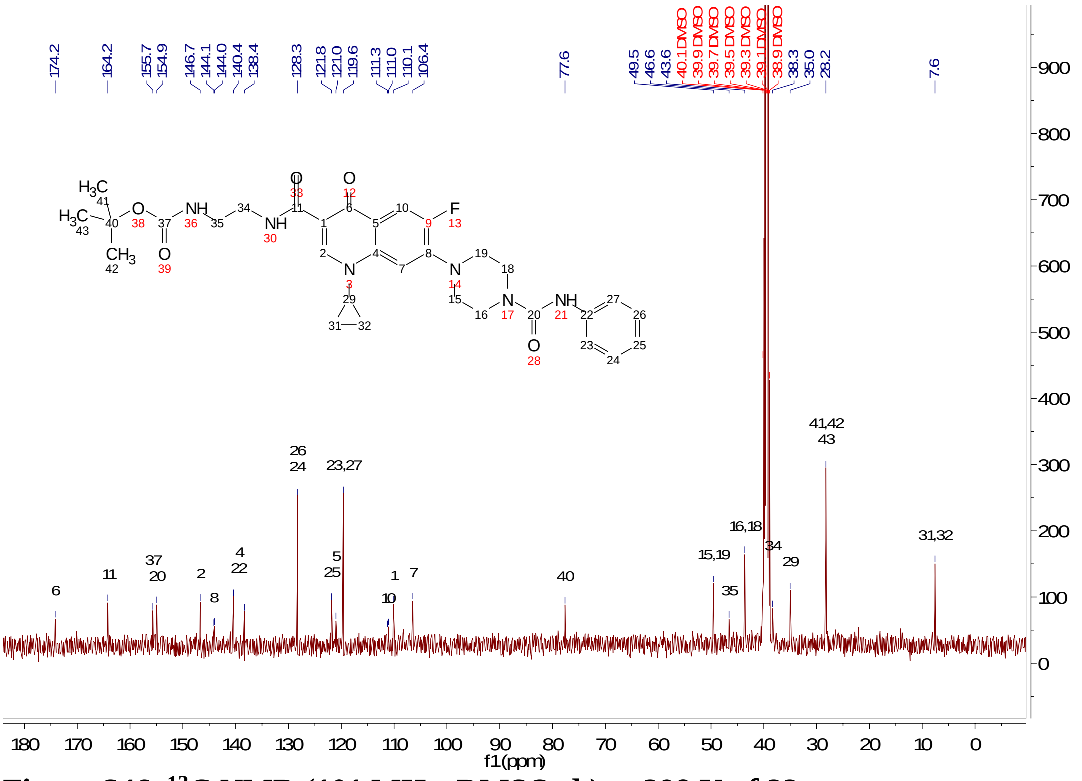

Figure S46. ^{13}C NMR (101 MHz, DMSO-d_6) at 298 K of 22.

***N*-(2-Aminoethyl)-1-cyclopropyl-6-fluoro-4-oxo-7-(4-(phenylcarbamoyl)piperazin-1-yl)-1,4-dihydroquinoline-3-carboxamide (23)**

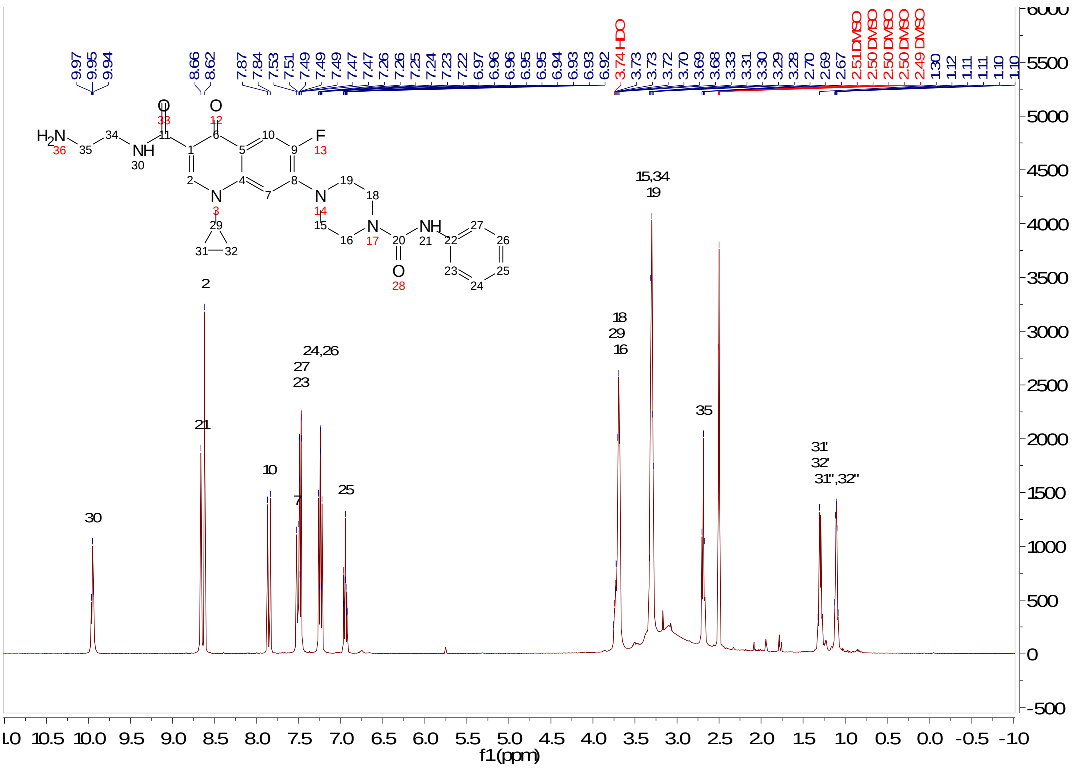

Figure S47. ^{1}H NMR (400 MHz, DMSO-d_6) at 298 K of 23.

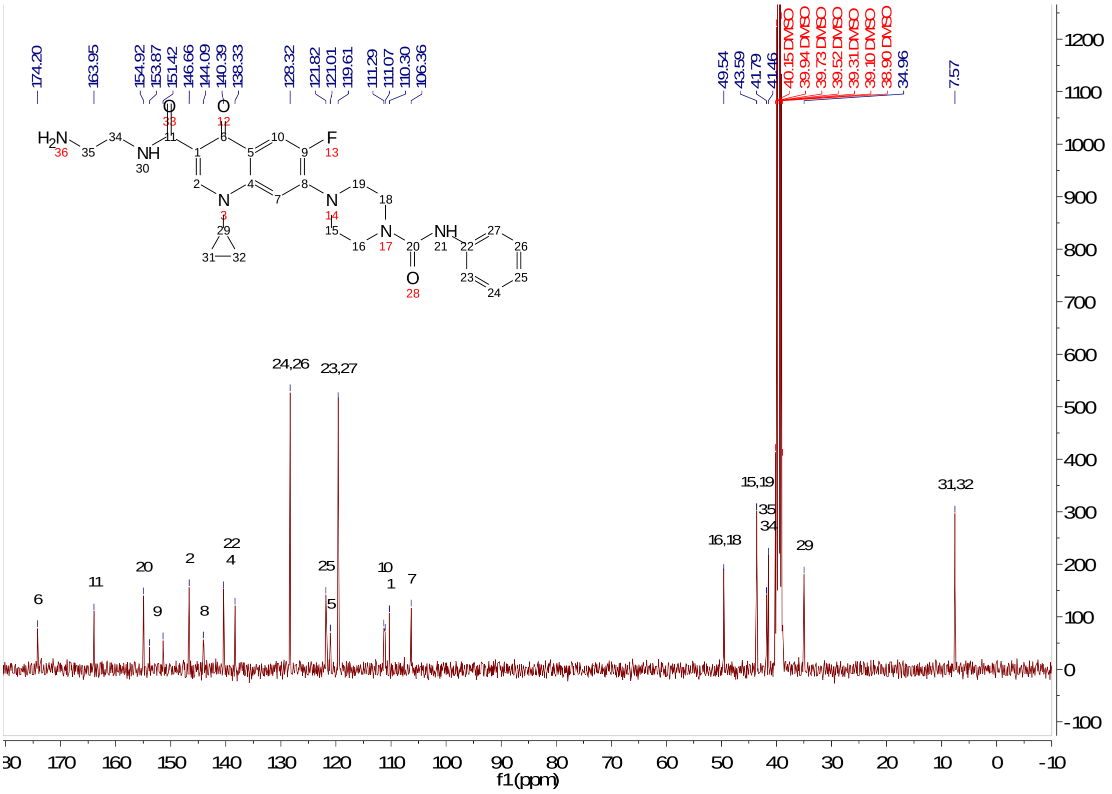

Figure S48. ^{13}C NMR (101 MHz, DMSO-d_6) at 298 K of 23.

2.6. NMR spectra of intermediate compounds

2.6.1. 7-(4-(*tert*-Butoxycarbonyl)-3-methylpiperazin-1-yl)-1-cyclopropyl-6-fluoro-4-oxo-1,4-dihydroquinoline-3-carboxylic acid (Q2a).

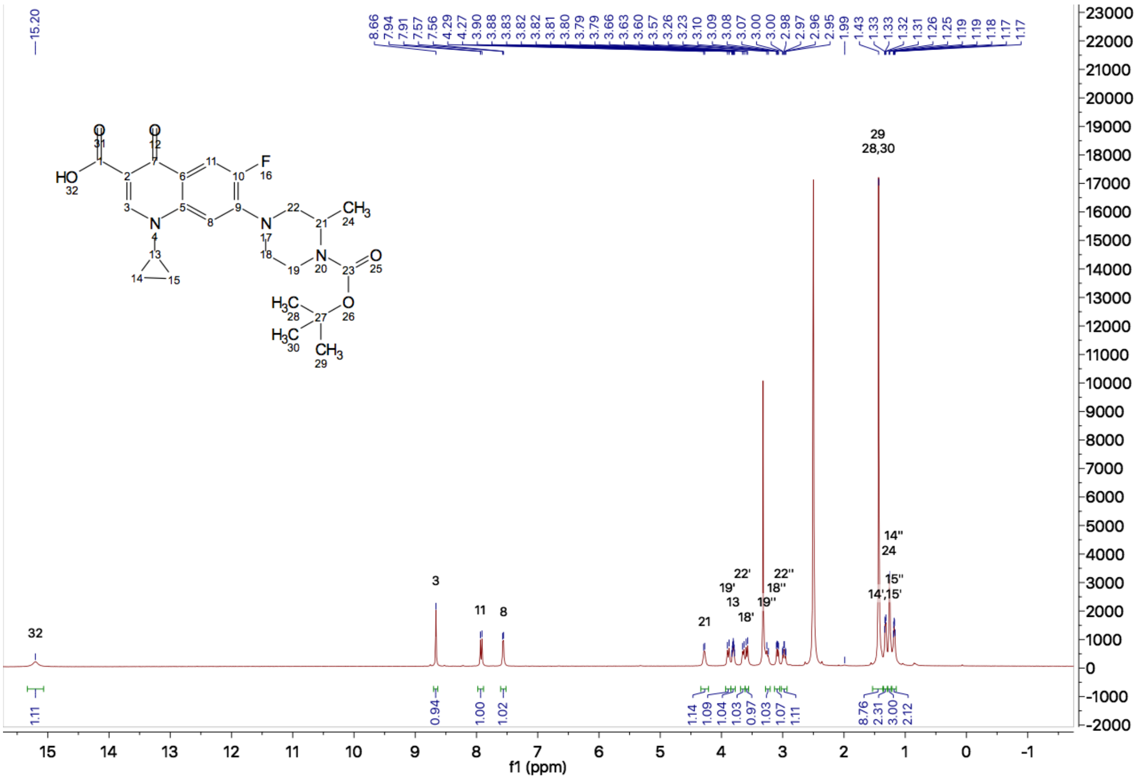

Figure S44. **^{1}H NMR (500 MHz, DMSO-d_6) at 298 K of Q2a.**

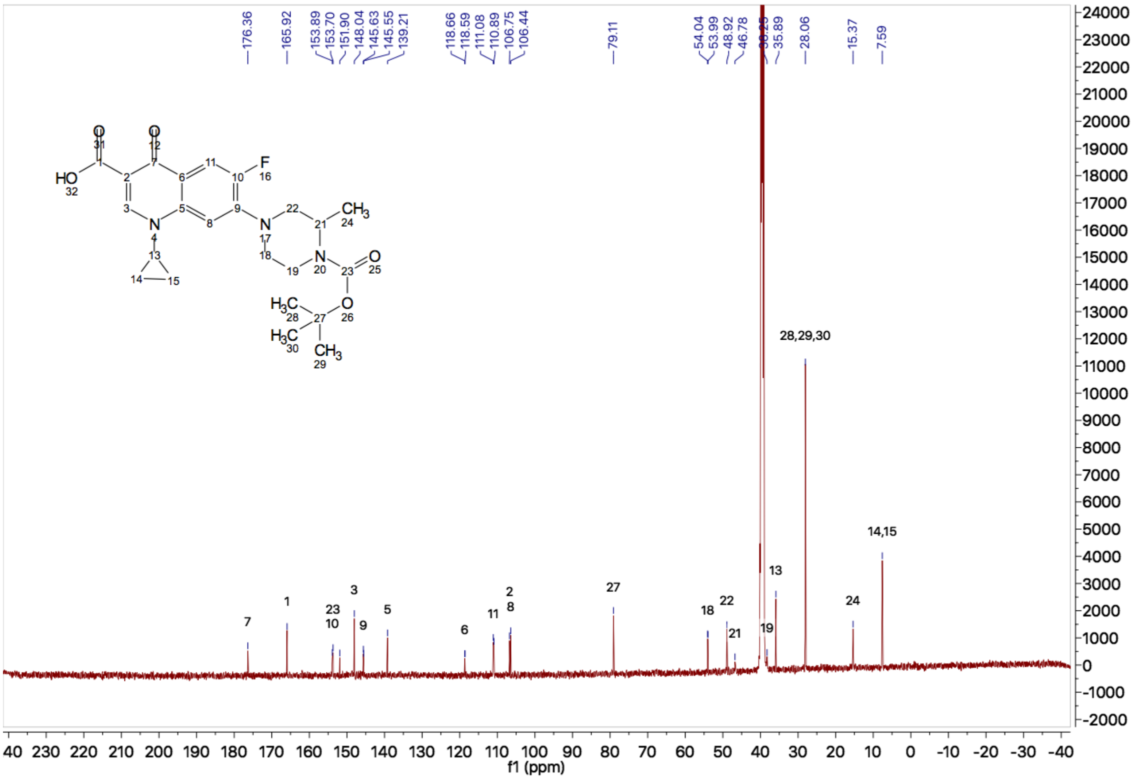

Figure S45. **^{13}C NMR (500 MHz, DMSO-d_6) at 298 K of Q3.**

2.6.2. 7-(3-((*tert*-Butoxycarbonyl)amino)pyrrolidin-1-yl)-1-cyclopropyl-6-fluoro-4-oxo-1,4-dihydroquinoline-3-carboxylic acid (Q2b).

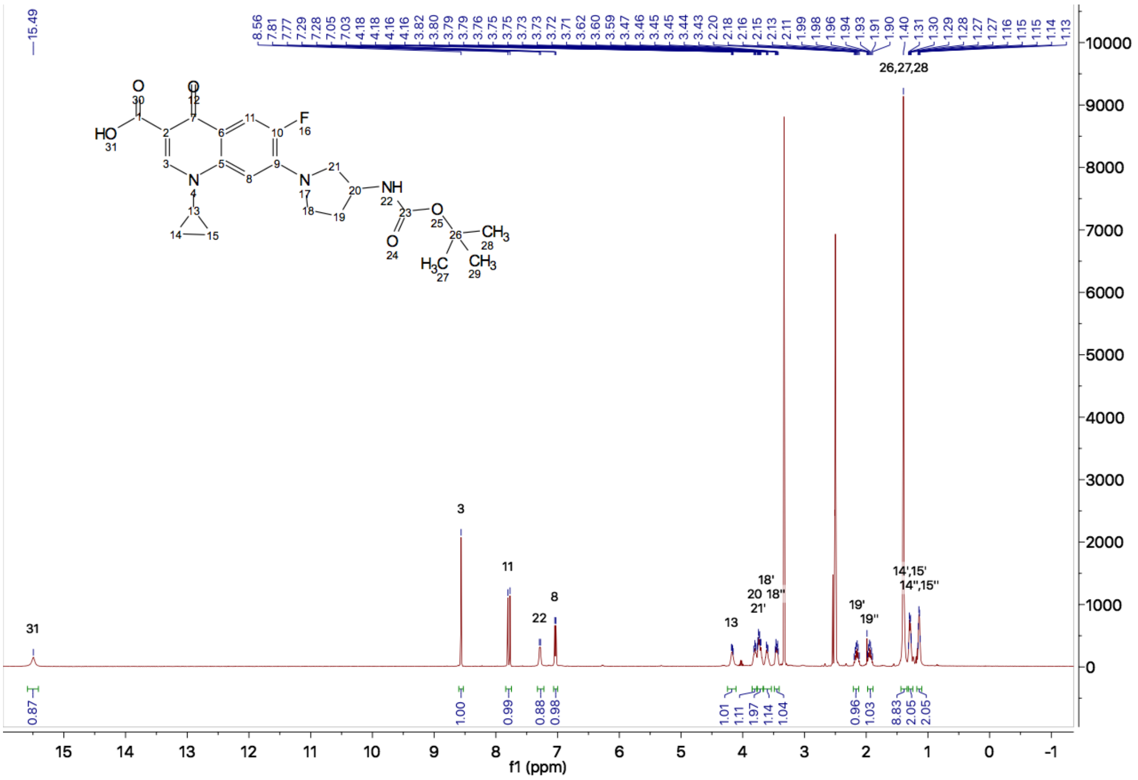

Figure S46. ^{1}H **NMR (500 MHz, DMSO-d_6) at 298 K of Q2b.**

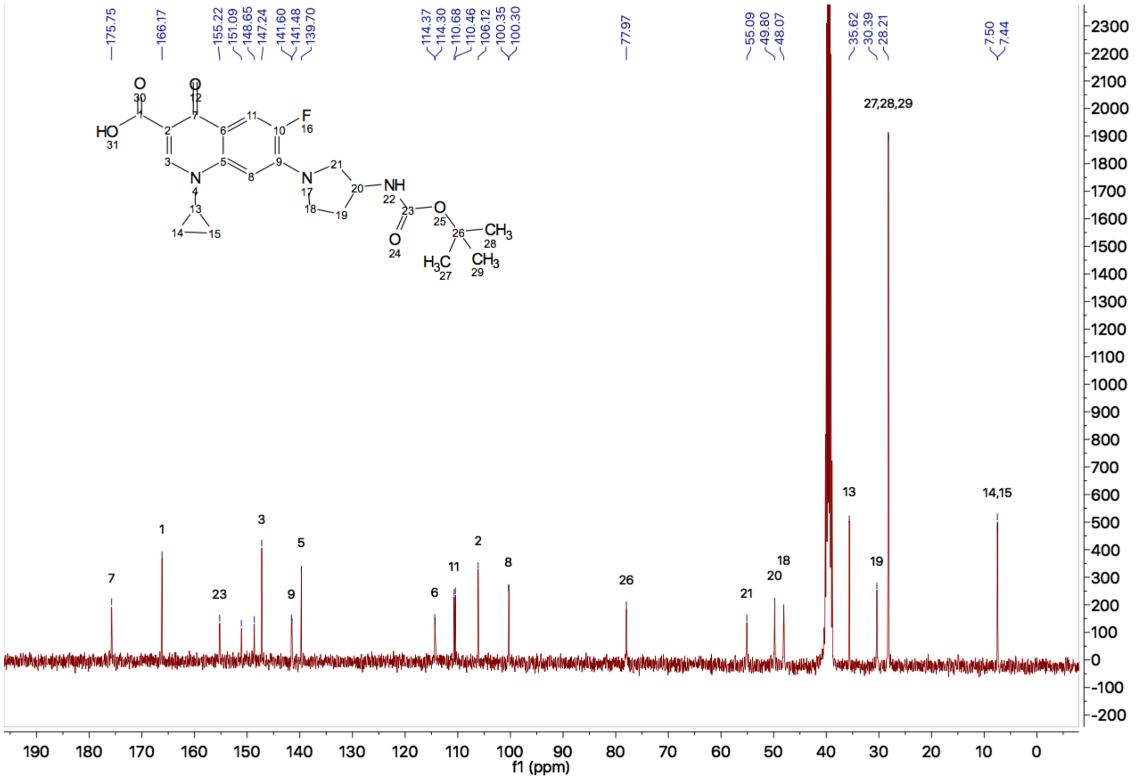

Figure S47. ^{13}C **NMR (400 MHz, DMSO-d_6) at 298 K of Q2b.**

2.6.3. 7-((2-((*tert*-Butoxycarbonyl)amino)ethyl)amino)-1-cyclopropyl-6-fluoro-4-oxo-1,4-dihydroquinoline-3-carboxylic acid (Q2c).

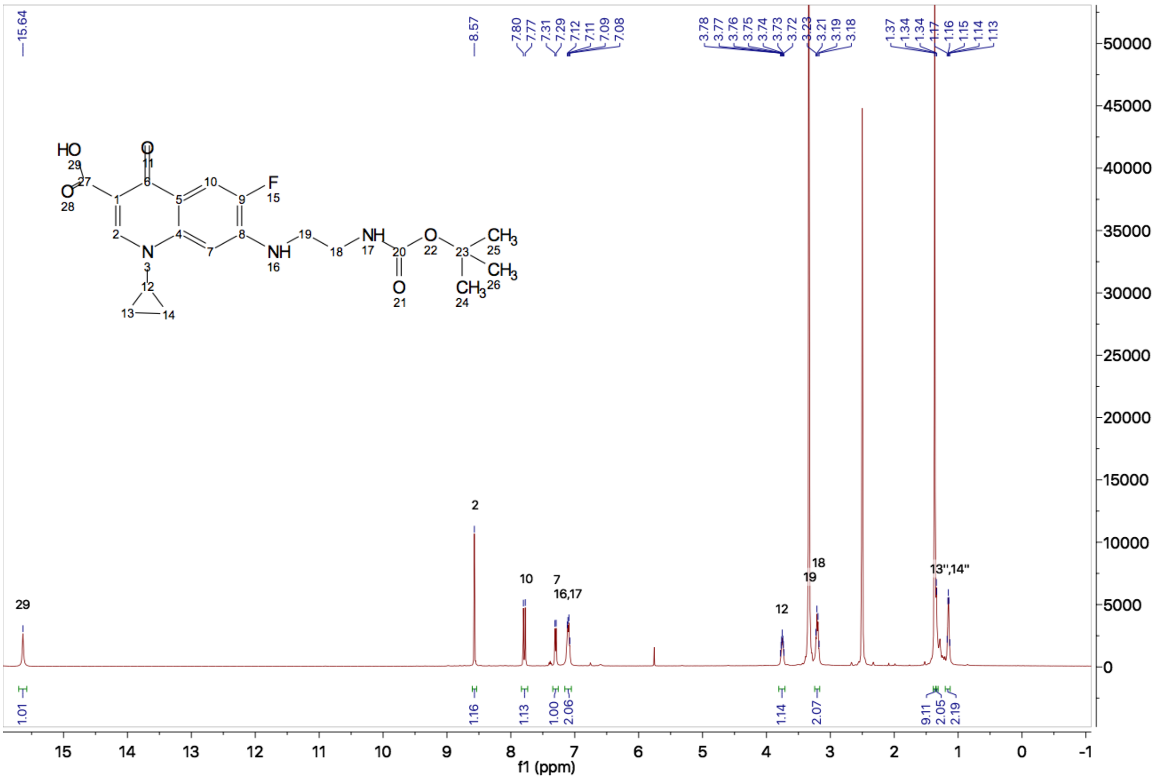

Figure S48. ^{1}H NMR (400 MHz, DMSO-d_6) at 298 K of Q2c.

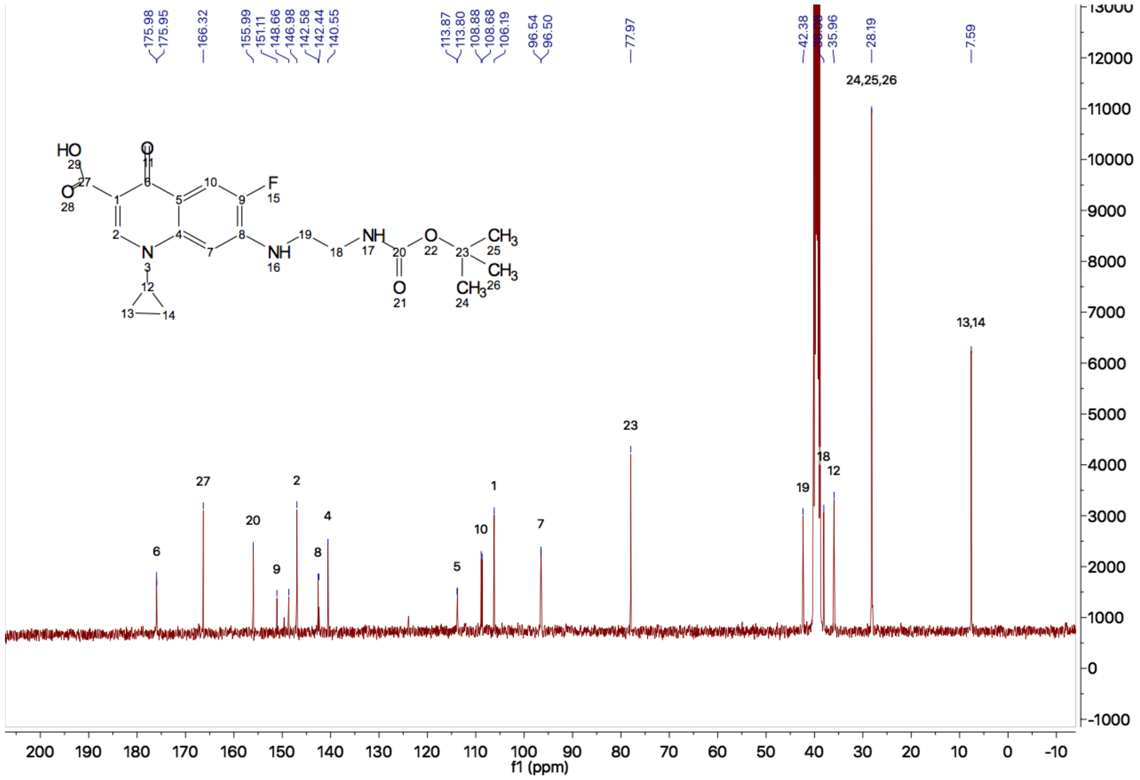

Figure S49. ^{13}C NMR (400 MHz, DMSO-d_6) at 298 K of Q2c.

3. References

1. Baba T, Ara T, Hasegawa M, et al. Construction of *Escherichia coli* K-12 in-frame, single-gene knockout mutants: the Keio collection. *Mol Syst Biol*. 2006;2(1):2006.0008. doi:10.1038/msb4100050

2. Horsburgh MJ, Aish JL, White IJ, Shaw L, Lithgow JK, Foster SJ. σB Modulates Virulence Determinant Expression and Stress Resistance: Characterization of a Functional rsbU Strain Derived from Staphylococcus aureus 8325-4. *J Bacteriol*. 2002;184(19):5457-5467. doi:10.1128/JB.184.19.5457-5467.2002

3. Diep BA, Gill SR, Chang RF, et al. Complete genome sequence of USA300, an epidemic clone of community-acquired meticillin-resistant Staphylococcus aureus. *The Lancet*. 2006;367(9512):731-739. doi:10.1016/S0140-6736(06)68231-7

4. Wiegand I, Hilpert K, Hancock REW. Agar and broth dilution methods to determine the minimal inhibitory concentration (MIC) of antimicrobial substances. *Nat Protoc*. 2008;3(2):163-175. doi:10.1038/nprot.2007.521

5. Bannister TD, Nair R, Spicer T, et al. ML328: A Novel Dual Inhibitor of Bacterial AddAB and RecBCD Helicase-nuclease DNA Repair Enzymes. In: *Probe Reports from the NIH Molecular Libraries Program*. Bethesda (MD): National Center for Biotechnology Information (US); 2010. http://www.ncbi.nlm.nih.gov/books/NBK148492/. Accessed November 29, 2016.

6. Lippur K, Tiirik T, Kudrjashova M, Järving I, Lopp M, Kanger T. Amination of quinolones with morpholine derivatives. *Tetrahedron*. 2012;68(47):9550-9555. doi:10.1016/j.tet.2012.09.082
